# A restatement of the natural science evidence base concerning the health effects of low-level ionizing radiation

**DOI:** 10.1098/rspb.2017.1070

**Published:** 2017-09-13

**Authors:** Angela R. McLean, Ella K. Adlen, Elisabeth Cardis, Alex Elliott, Dudley T. Goodhead, Mats Harms-Ringdahl, Jolyon H. Hendry, Peter Hoskin, Penny A. Jeggo, David J. C. Mackay, Colin R. Muirhead, John Shepherd, Roy E. Shore, Geraldine A. Thomas, Richard Wakeford, H. Charles J. Godfray

**Affiliations:** 1 Department of Zoology, University of Oxford, South Parks Road, Oxford OX1 3PS, UK; 2 Oxford Martin School, University of Oxford, 34 Broad Street, Oxford OX1 3BD, UK; 3 Barcelona Institute for Global Health (ISGlobal), Center for Research in Environmental Epidemiology (CREAL), Barcelona, Spain; 4 College of Medical, Veterinary and Life Sciences, Wellcome Surgical Institute, University of Glasgow, Garscube Estate, Glasgow G61 1QH, UK; 5 Medical Research Council, Harwell, Didcot OX11 0RD, UK; 6 Center for Radiation Protection Research, Department of Molecular Biosciences, The Wenner-Gren Institute, Stockholm University, Stockholm, Sweden; 7 Christie Medical Physics and Engineering, Christie Hospital and University of Manchester, Manchester, UK; 8 Mount Vernon Cancer Centre, Northwood HA6 2RN, UK; 9 Genome Damage and Stability Centre, University of Sussex, Science Park Road, Falmer, Brighton BN1 9RQ, UK; 10 Department of Engineering, University of Cambridge, Trumpington Street, Cambridge CB2 1PZ, UK; 11 Institute of Health and Society, Newcastle University, Baddiley-Clark Building, Richardson Road, Newcastle upon Tyne NE2 4AX, UK; 12 Ocean and Earth Science, University of Southampton, Southampton SO14 3ZH, UK; 13 Radiation Effects Research Foundation, Hiroshima, Japan; 14 Department of Surgery and Cancer, Imperial College London, Room 11L04, Charing Cross Hospital, Fulham Palace Road, London W6 8RF, UK; 15 Centre for Occupational and Environmental Health, Institute of Population Health, Faculty of Medical and Human Sciences, University of Manchester, Ellen Wilkinson Building, Oxford Road, Manchester M13 9PL, UK

**Keywords:** radiation, epidemiology, cancer, radon, nuclear, evidence for policy

## Abstract

Exposure to ionizing radiation is ubiquitous, and it is well established that moderate and high doses cause ill-health and can be lethal. The health effects of low doses or low dose-rates of ionizing radiation are not so clear. This paper describes a project which sets out to summarize, as a restatement, the natural science evidence base concerning the human health effects of exposure to low-level ionizing radiation. A novel feature, compared to other reviews, is that a series of statements are listed and categorized according to the nature and strength of the evidence that underpins them. The purpose of this restatement is to provide a concise entrée into this vibrant field, pointing the interested reader deeper into the literature when more detail is needed. It is not our purpose to reach conclusions on whether the legal limits on radiation exposures are too high, too low or just right. Our aim is to provide an introduction so that non-specialist individuals in this area (be they policy-makers, disputers of policy, health professionals or students) have a straightforward place to start. The summary restatement of the evidence and an extensively annotated bibliography are provided as appendices in the electronic supplementary material.

## 1. Introduction

**Figure 1.**
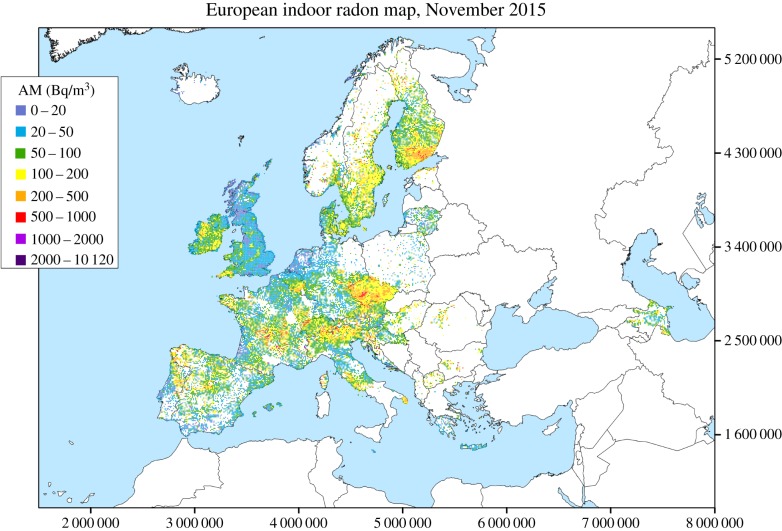
European indoor radon map, November 2015. The map shows arithmetic means (AM) over 10×10 km cells of long-term radon concentration in ground floor rooms. The cell mean is neither an estimate of the population exposure, nor of the risk. The data are from the European Commission Joint Research Centre (JRC), Institute for Transuranium Elements (ITU), REM project. Reproduced with permission from Hoffman *et al.* [1]. (Online version in colour.)

Ionizing radiation is radiation that carries enough energy that it can ionize atoms or molecules (i.e. strip electrons from them) as it passes through matter. Life on the Earth has always been exposed to ionizing radiation from natural sources, for example radon gas (figure 1). During the past 120 years the development of medical, military and industrial uses of radiation has created additional exposure from man-made sources. Large doses of radiation are known to be detrimental to the health of organisms including humans, but the health effects of low doses and doses delivered at low dose-rates are not completely clear. A ‘low dose’ of radiation has been defined by several organizations as 100 milligray (mGy) or less of sparsely ionizing radiation (e.g. electrons), and a ‘low dose-rate’ as less than 0.1 mGy min^−1^ of sparsely ionizing radiation when averaged over about 1 h. The sievert (Sv) is a non-physical derived unit used in the context of radiological protection, which weights the amount of energy deposited in tissue (the absorbed dose measured in gray (Gy), where 1 Gy = 1 J kg^−1^) by different types of radiation (giving equivalent dose, in Sv), and the relative sensitivity of tissues (giving effective dose, in Sv) to probabilistic (stochastic) effects such as cancer induction by low doses or low dose-rates. The weights used in calculating effective dose are based upon an expert consensus grounded in scientific evidence, but with elements of subjectivity [2,3]. Effective dose is a measure of detriment devised for the purposes of protection, so that doses from different sorts of radiation and dose distributions can be combined appropriately. In the UK, the average annual effective dose from natural background radiation is 2.3 mSv [4], so an accumulated dose of 100 mSv would commonly arise over 40–50 years of exposure at a low dose-rate. Any medical exposure (or other man-made exposure) would be additional to that.

There is an international system of radiological protection which considers situations of planned, existing and emergency exposures and, specifically for planned exposures, recommends annual limits on the amount of additional effective dose that should not be exceeded. Those annual limits are 1 mSv for the public and 20 mSv for radiation workers (these limits exclude natural background radiation and radiation doses received in medical procedures) [5]. Most countries use these recommendations in their legislation, but some do not. For example in the USA, the annual limit for workers is 50 mSv [6]. These limits, and the radiological protection system in general, are based upon the ‘linear, no threshold’ (LNT) dose–response model, which assumes that the excess risk from low-level exposure is directly proportional to dose and that there is no dose so small that it has no effect. On this basis, it is recommended that all relevant doses should be summed to ensure that individuals do not exceed the annual limits.

Both the LNT model and the dose limits are widely debated [7,8]. Some believe that they are too strict and impose unreasonable costs on the use of radiation. Others believe that they are not strict enough and allow too much risk from radiation exposure. A widely accepted illustration of the approximate magnitude of the risk from a low dose is that if 100 individuals were each exposed to 100 mSv then, over a lifetime, approximately one of them would be expected to develop a radiation-induced cancer, whereas around 42 of them would be expected to develop cancer from other causes [9]. Although the potential risks considered here may be small for any individual, very large populations may be exposed, so the magnitude of these health effects should not be dismissed as unimportant [10].

Within a year of Röentgen's 1895 discovery of X-rays [11], dermatitis caused by high-dose X-ray radiation had been described [12] and protective measures to reduce exposure were already advised [13]. In the ensuing 120 years, a large literature has established a detailed, quantitative description of the health risks of radiation. Both sides of the debate about risks from low-level exposure cite this underlying natural science evidence base in support of their arguments. The aim of the project described here is to provide a ‘restatement’ of that evidence base in a succinct and accessible manner to a non-technical reader.

## 2. Methods

A preliminary draft review of the literature on health risks of low-level radiation was constructed. At a 1-day workshop, most authors met to discuss the different evidence components. A second draft was then made and each piece of evidence was assigned a descriptor. Because of the very extensive nature of the underlying evidence base, we devised a set of categories that are broadly speaking a ranked score of the strength and consistency of the supporting evidence. In these descriptors, a ‘well-powered study’ means a study that has high probability of detecting an effect of a given size when that relationship genuinely exists. Statements are considered to be supported by:

[*C*_ons_] data support a consensus based upon a single well-powered study, or one or more pooled analyses with consistent results, or several lower powered studies with consistent results;
[*E*_mco_] data support an emerging consensus based upon a single, well-powered study (which may be an individual study or a pooled analysis), but in a context where other studies report disparate results or repeat analyses have not yet been performed;
[*N*_oco_] there is no consensus interpretation because the data are insufficient in quantity or too variable; and
[*P*_rojn_] projections based on available evidence but with substantial uncertainties.

The second draft was sent to 16 scientists who are experts in low-level radiation including representatives from academia, government and non-governmental organizations.

The project was funded by the Oxford Martin School (part of the University of Oxford) and though many groups were consulted, the project was conducted completely independently of any stakeholder.

This is not a systematic review and the categorization of the evidence statements represents the opinion of the authors arrived at through debate, but not through other formal consensus procedures. Systematic reviews of the literature on the health effects of ionizing radiation exist elsewhere and are hundreds of pages long (e.g. [5,9,14]).

## 3. Results

The full summary of the natural science evidence base is given as a restatement of the evidence in appendix A with an annotated bibliography as appendix B, both in the electronic supplementary material. Each section of the restatement ends with a short paragraph summarizing the evidence. Those ‘summaries of summaries’ are presented here.

### (a) Definitions and units

The absorbed dose of radiation is quantified in gray (Gy) and is the amount of energy deposited in joules per kilogram. Equivalent dose and effective dose use weightings of absorbed dose and are described in sievert (Sv). For the purposes of radiological protection at low-level exposure, recommendations regarding stochastic effects are issued using effective dose in sievert. Ill-effects of radiation are divided into two broad types: ‘harmful tissue reactions’ at higher doses and ‘stochastic effects’ (such as cancer) across all doses including lower doses.

### (b) Background exposure and uncertainties at low dose

**Figure 2.**
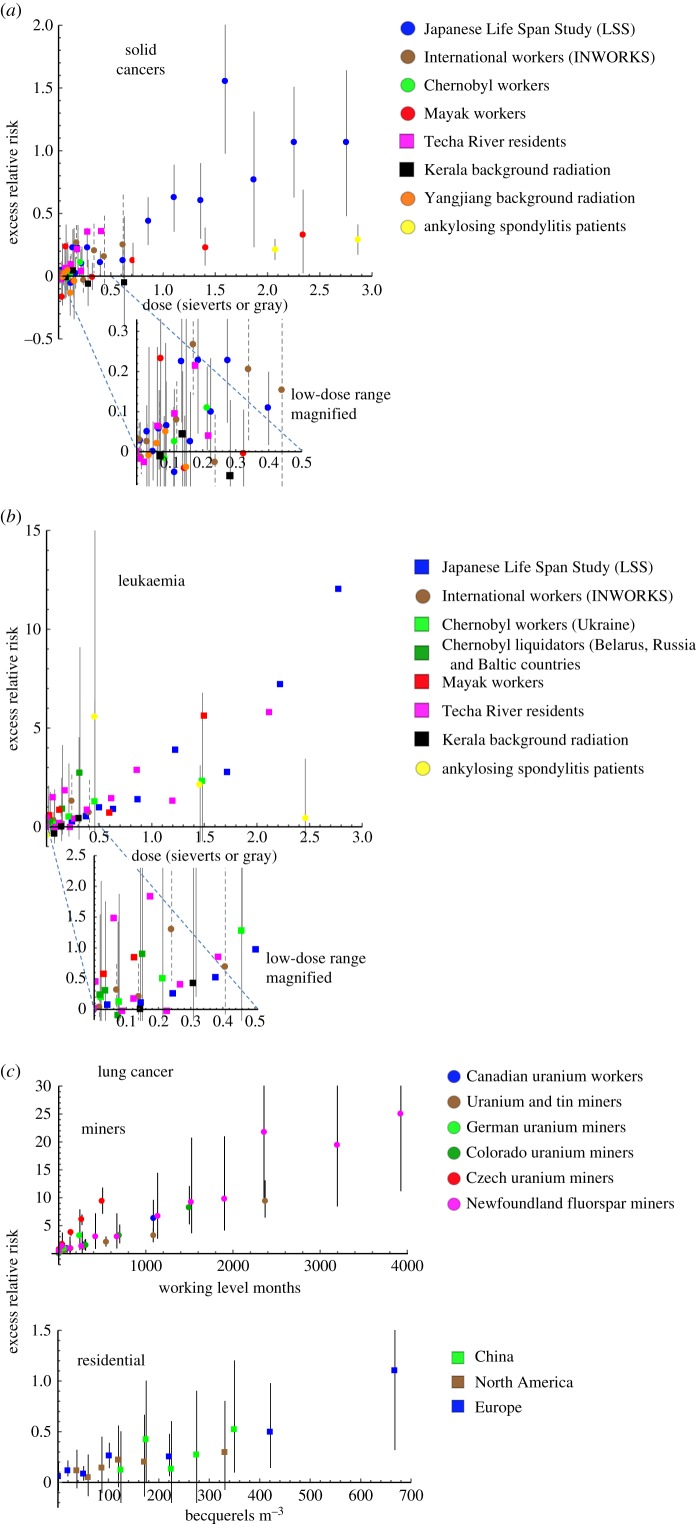
Estimates of excess relative risk of cancers from large epidemiological studies. The cohorts include a variety of exposure types including via nuclear weapons, occupational exposure in mines or nuclear facilities, environmental contamination from nuclear facilities, naturally high background radiation, medical therapy and radon. Outcomes are mortality (round data points) or incidence (square data points). Confidence intervals have been added where they are available. Dashed lines denote 90% CIs and solid lines denote 95% CIs. Some confidence intervals exceed the range of the *y*-axis. Table 8 in the electronic supplementary material, at paragraph 23 annotated bibliography (appendix B) contains further detail on these datasets, and see paragraph 37 for explanations of epidemiological association measures used. (*a*) Solid cancers. The Japanese Life Span Study (LSS) data are for solid cancer mortality in the cohort of survivors of the Japanese atomic bombings. The international workers data are for mortality from all cancers excluding leukaemia in a cohort of French, US and British nuclear workers (INWORKS). The Chernobyl workers data are for solid cancer mortality in a cohort of Russian Federation clean-up workers. The Mayak workers data are for mortality from solid cancers excluding bone, lung and liver cancer in workers at the Mayak weapons plant in Russia. The Techa River residents data are for solid cancer incidence in the cohort of individuals living downstream from the Mayak plant. The Kerala background radiation data are for cancer incidence excluding leukaemia in a cohort of residents of a high background radiation area in India. The Yangjiang background radiation data are for solid cancer mortality in a cohort of residents of a high background radiation area in China. The ankylosing spondylitis data are for solid cancer mortality among UK patients with ankylosing spondylitis treated with X-rays. (*b*) Leukaemia, excluding CLL. The Japanese Life Span Study (LSS) data are for leukaemia incidence in the cohort of survivors of the Japanese atomic bombings, excluding both CLL and ATL. The international workers data are for mortality from leukaemia excluding CLL in a cohort of French, USA and British nuclear workers (INWORKS). The Chernobyl workers data are for leukaemia incidence excluding CLL in a cohort of Ukrainian clean-up workers, and the Chernobyl liquidators data are for leukaemia incidence excluding CLL in a cohort of workers from Belarus, Russia and Baltic countries. The Mayak workers data are for incidence of leukaemia excluding CLL in workers at the Mayak weapons plant in Russia. The Techa River residents data are for leukaemia incidence excluding CLL in a cohort of individuals living downstream from the Mayak weapons plant in Russia. The Kerala background radiation data are for leukaemia incidence excluding CLL in a cohort of residents of a high background radiation area in India. The ankylosing spondylitis data are for leukaemia excluding CLL mortality among UK patients with ankylosing spondylitis treated with X-rays. (*c*) Lung cancer following radon exposure. Because of the difference in magnitude in exposures to radon between mining and residential contexts, studies have been split into two charts. The top chart denotes six studies of lung cancer mortality in miners of uranium, tin or fluorspar in relation to cumulative exposure (in ‘working level months’). The uranium and tin miners study consists of 11 pooled international cohorts (including the Newfoundland and Czech cohort). The Newfoundland and Czech single cohort studies have been more recently updated for results and have therefore also been drawn separately. The bottom chart shows residential studies in relation to radon concentration (in Bq m^−3^). The Chinese residential data are for lung cancer incidence across China; the North America residential data are for lung cancer incidence across North America; and the European residential data are for lung cancer incidence across Europe. (Online version in colour.)

**Figure 3.**
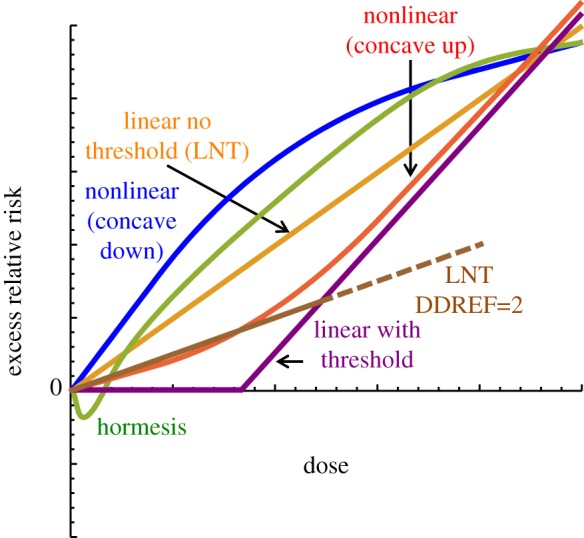
Potential risk models, relating risk of disease and dose of radiation at low dose and low dose-rate. The different models are described in the electronic supplementary material, appendices A and B, paragraph 24. At sufficiently low doses, all models are consistent with available datasets. Adapted from [15].

Across the world, the average effective dose from natural background radiation is 2.4 mSv yr^−1^. Large epidemiological studies can be used to estimate the health risk of higher doses and, through statistical calculation of confidence intervals, infer that risks are greater than zero. But at doses in the range of the natural background, even the largest epidemiological studies have substantial difficulties in reliably distinguishing between low risk and zero risk (figure 2). Radiobiological knowledge of relevant processes following low-level exposure is incomplete and therefore point estimates for low dose or low dose-rate risks above the background are inferred by extrapolation from the results of epidemiological studies at higher doses. Several different models can be used for such extrapolation and most are largely consistent with the low-level data available (figure 3).

### (c) Acute high-dose exposures

High doses are described in units of gray. With a whole-body acute dose of greater than 15 Gy, death is certain within 5 days. With a whole-body acute dose of 2.5–5 Gy, without good medical care, death owing to bone marrow damage may follow within two months in around 50% of healthy adults exposed. With a whole-body acute dose of 1 Gy, without good medical care, death owing to bone marrow damage may follow in about 10% of those exposed. Doses above about 0.5 Gy will depress blood-forming processes over the coming week and cause a range of other morbidities including erythema, epilation and sterility. Cataracts and damage to the circulatory system that may become apparent many years later are also caused at doses above about 0.5 Gy; whether or not lower doses cause cataracts and circulatory disease is a topic of ongoing study and debate. Even at high doses no statistically significant excess of hereditary effects have been seen in the offspring of people who were exposed prior to conception, although animal experiments do show such effects and imply that they may occur at a very low frequency in humans.

### (d) Low dose exposures

The primary ill-health caused by low to moderate doses of ionizing radiation is cancer, although the possibility of non-cancer effects (particularly cardiovascular disease) is of increasing concern. Very large studies would be required to detect the ill-effects of doses of around 1–10 mSv. Doses of this size are routinely encountered—for example, from natural background radiation and medical diagnostic exposures. Radiation epidemiology is primarily informed by studies that compare individuals with varying levels of radiation exposure.

### (e) The Japanese Life Span Study

The study of survivors of the atomic bombings of Japan (the Japanese Life Span Study; LSS) is the largest and longest study of risks from ionizing radiation. It is treated as the ‘gold standard’ in the sense that the results of other studies are compared with its results. Its headline results are that at 1 Gy (dose to the colon) the risk of mortality from solid cancer is raised by 50% and at 1 Gy (dose to the red bone marrow) the risk of mortality from leukaemia is quadrupled. Note that the excess relative risk quoted here is different from the nominal excess absolute lifetime risk coefficient for cancer of 5.5% per Sv derived by the ICRP and used in optimization calculations. Excess relative risk (the proportional increase in risk) is only meaningful in the context of the underlying risk in an unexposed population. So, for example, in the LSS to 2003 there were 50 620 deaths, of which 10 929 were from solid cancers, and 318 from leukaemia. Thus, even though the excess relative risk at 1 Gy is much higher for leukaemia than for solid cancer, around 525 of the solid cancer deaths and only around 105 of the leukaemia deaths are estimated to be radiation-associated. Large studies of individuals conceived to parents who were survivors of the atomic bombings find no statistically significant adverse effects.

### (f) The Chernobyl nuclear power plant accident

A number of early emergency workers at the accident at the Chernobyl nuclear power plant received high doses which produced tissue reactions and 28 early deaths. The long-term health impacts are contested. There is consensus on two major health impacts: thyroid cancers caused by high levels of exposure of children to radioactive iodine, and ill-effects to mental health caused by widespread fear of potential risks and social disruption. There is emerging evidence on the risk of leukaemia among recovery workers and those risks are broadly in line with what is expected from the LSS. At present, there is little convincing evidence of other radiation-associated effects in recovery workers or the wider public.

### (g) The Fukushima Dai-ichi nuclear power plant accident

The Fukushima Dai-ichi nuclear power plant accident has caused substantial ill-health through the effects of the evacuations, continued displacement and fear of radiation. It is unclear if there will be a detectable excess in thyroid cancer in the coming years. No other discernible increase in ill-health attributable to radiation exposure is expected in either emergency workers or members of the public.

### (h) Studies of workers exposed to radiation

Workers in the nuclear industries often have both external and internal radiation exposure. Their risks from external doses for solid cancer and leukaemia are consistent with those observed in the LSS even though their doses are accumulated at low dose-rates over many years. In the International Nuclear Workers Study (INWORKS), even among those who have total accumulated doses below 100 mGy, the risk of mortality from solid cancer is consistent with the LSS estimate (although the confidence intervals are wide). Radiologists and radiation technicians who worked during the early years have increased risks of leukaemia, skin cancer and, for women, breast cancer. More recent cohorts (from an era of lower doses to workers) have not yet displayed excess risks, but are still young. Cataract risk may be increased in medical workers who use X-ray imaging to guide interventions. Underground hard rock miners have an elevated risk of lung cancer roughly in proportion to their exposure to radon gas and its radioactive progeny.

### (i) Environmental exposure

Radon in the home increases the risk of lung cancer, particularly for smokers. Regions of the world with high natural background radiation do not consistently show an excess risk of solid cancers even in large studies. Fallout from nuclear weapons testing caused low-level internal exposures that were concentrated in time and, to a lesser extent, space, with risks of childhood leukaemia that are consistent with the risks estimated from the LSS. There have been clusters of childhood leukaemia close to and away from nuclear installations that remain unexplained.

### (j) Medical exposure

After adjustment for dose fractionation and high-dose cell killing, the risks posed by radiation received as therapy are broadly in line with LSS data. Doses from diagnostic X-rays are much lower, but some studies describe raised risks of childhood leukaemia and other childhood cancers after *in utero* exposure. Recent studies of leukaemia and brain cancer after childhood computed tomography (CT) scans report raised risks, but the extent to which the pre-existing health status of the patients might confound this association needs further consideration. The principle of justification emphasizes that health benefits of radiation use in medicine must outweigh any radiation exposure risks.

### (k) Experimental studies of mechanisms of damage

Studies *in vitro* have clearly established that radiation can damage DNA in ways that if mis-repaired could, *in vivo,* lead to cancer. Because of the stochastic nature of interactions of radiation with DNA and other molecules, it is reasonable to expect initial damage at low doses to have a linear dose–response, but subsequent cellular responses may not have a linear dose–response and may be different at low versus high doses. Despite much elucidation of the underlying cellular processes, it is still not clear precisely what steps are necessary and sufficient for a dose of radiation to eventually lead to cancer (sometimes decades later). Currently, there are no validated bio-markers of radiation-induced cancers. Understanding of the mechanisms whereby radiation causes cardiovascular disease and cataracts is still less advanced.

### (l) Experimental studies that inform risk assessment

Studies *in vitro* demonstrate a linear dose–response for chromosome aberrations at doses between 20 and 100 mGy. Irradiation of animals has clearly established that moderate and high doses of radiation (usually 100 mGy to several Gy) can cause cancer and life-shortening (also largely owing to cancer). Dose–response relationships at low dose are mostly linear. Irradiation of male mice before mating has demonstrated that radiation-induced mutations can be passed to offspring in a manner that is proportional to parental dose. Analysis comparing dose–response slopes at low and high doses implies that radiation delivered at a low dose or a low dose-rate carries two-to fourfold less risk than acute doses of the same total dose. An equivalent analysis that combines human epidemiological data and animal experimental data estimated that the dose and dose-rate effectiveness factor may only be about 1.5-fold and this factor is under further investigation.

### (m) Perspectives

Compared with other common health risks (obesity, tobacco smoking, exposure to ambient particulate air pollution), the number of years of life lost owing to radiation exposure is small.

## 4. Discussion

This restatement appears in the context of a global system designed to integrate and summarize the body of knowledge on the outcome of human exposure to ionizing radiation. That system starts with a disseminated, natural science endeavour that produces a primary literature [16]; continues with several national and international bodies that synthesize the literature on the biology and epidemiology of radiation risks [9,14]; produces recommendations based upon the science [5]; which are turned into safety standards [17]; and then enacted as international and national law [18]. The published syntheses are very much longer and more detailed than this restatement. It is our aim to provide a concise introduction that can point the interested reader deeper into the literature when more detail is needed.

There are several aspects of radiation risk that we do not attempt to address. For example, we do not cover the science of estimating the dispersal of radionuclides after a release; a review is provided by Yao [19]. Nor do we cover the regulation of radioactivity in food [20]. The project considers only the natural science evidence base (although we make some reference to the psycho-social science of the impact of accidents). There are other important social science issues involved in the making of policy around radiation risks. Among these are economic considerations of the valuation of any damage caused, and the costs associated with radiation protection [21] and clean-up operations [22,23]. Finally, in focusing on human health effects of radiation, we have not considered environmental impacts and do not discuss the effects of radiation contamination on wildlife [24].

The purpose of this restatement is not to reach conclusions on whether the legal limits on radiation exposures are too high, too low or just right, nor to declare whether it is defensible to use the LNT model to approximate the risks of stochastic effects (largely cancer but including hereditary effects). Our purpose is to provide an entrée to this large and vibrant literature so that non-specialist individuals with responsibility for making or disputing policy in this area have a straightforward place to start.

## Supplementary Material

APPENDIX A

## Supplementary Material

APPENDIX B

## Data accessibility

This article has no additional data.
