# Supplementary material for: A restatement of the natural science evidence base concerning the health effects of low-level ionizing radiation
Source: Proc Biol Sci. 2017 Sep 13;284(1862):20171070. doi: 10.1098/rspb.2017.1070 (PMC5597830; doi:10.1098/rspb.2017.1070)
Supplement: APPENDIX A [file rspb20171070supp1.pdf]

Appendix A
A restatement of the natural science evidence base concerning the health effects of low-level ionizing radiation

Proceedings of the Royal Society B DOI 10.1098/rspb.2017-1070

Angela R. McLean, Ella K. Adlen, Elisabeth Cardis, Alex Elliott, Dudley T. Goodhead, Mats Harms-Ringdahl, Jolyon H. Hendry, Peter Hoskin, Penny A. Jeggo, David J.C. Mackay, Colin R. Muirhead, John Shepherd, Roy E. Shore, Geraldine A. Thomas, Richard Wakeford & H. Charles J. Godfray

Paragraph numbering corresponds to that in the main document; annotated references are in Appendix B.

INTRODUCTION AND AIMS

1. Radiation is the emission and transmission of energy as electromagnetic waves or subatomic particles. “Ionizing radiation” can damage living things because it carries enough energy to strip electrons from atoms or molecules, leaving them electrically charged, a process called “ionization”.
2. A major mechanism whereby ionizing radiation harms living things is to damage DNA, including the creation of breaks in DNA strands. Although much has been learnt about the radiobiology of the processes that lead to adverse health effects consequent to radiation exposure, knowledge of mechanisms is insufficiently complete to permit the determination of risks from first principles. Thus, risk estimates must be obtained from (overwhelmingly observational) epidemiological studies of exposed groups, which usually pose interpretational challenges to some degree or another.
3. All people are exposed to ionizing radiation from both natural and man-made sources. Policy questions arise concerning how much exposure is acceptable and those questions become debatable and often contentious, particularly at low doses and low dose-rates, where the effects are small or uncertain because they are obscured by variability of the available data and by the high background incidence of cancer and other potentially radiation-induced diseases.
4. The aim here is to provide a succinct summary of the evidence-base relevant to policy-making in this area as of April 2017. This restatement also provides a consensus judgement by the authors on the level of confidence in the different evidence components; it presents a shared opinion based upon the studies listed in the annotated bibliography. For statements concerning evidence we use the following descriptors, indicated by abbreviated codes. In these descriptors a “well-powered study” means a study that has high probability of detecting an effect of a given size when that relationship genuinely exists. Statements are considered to be supported by:
   [C_{ons}] data support a **consensus** based upon a single well-powered study, or one or more pooled analyses with consistent results, or several lower powered studies with consistent results.
   [E_{mco}] data support an **emerging consensus** based upon a single, well-powered study (which may be an individual study or a pooled analysis), but in a context where other studies report disparate results or repeat analyses have not yet been performed.
   [N_{oco}] there is **no consensus** interpretation because the data are insufficient in quantity or too variable.
   [P_{rojn}] **projections** based on available evidence but with substantial uncertainties.
5. This review focuses on the natural science evidence relevant to radiation risks at low doses or low dose-rates although we do include some psycho-social impacts of accidents. The statements are based on evidence in the recognised peer-reviewed scientific literature and in the published summaries provided by authoritative bodies such as those of the United Nations and others.

THE SYSTEM OF RADIOLOGICAL PROTECTION

6. It is well established that moderate and high levels of exposure to radiation are harmful to human health and to other living things. For this reason there are systems of radiological protection designed to prevent or limit radiation-induced

damage, depending on the nature of this damage (see paragraph 18 below).

7. The pre-eminent body issuing such advice is the International Commission on Radiological Protection (ICRP); which is, in its own words, *"an independent, international organization with more than two hundred volunteer members from approximately thirty countries across six continents. These members represent the leading scientists and policy makers in the field of radiological protection."*

8. The ICRP's work is to "*contribute to an appropriate level of protection for people and the environment without unduly limiting the desirable human activities that may be associated with radiation exposure*."

    a. The ICRP does not have legislative power. Instead it issues recommendations which are widely used as the basis for national and international regulations and guidance. The ICRP publishes many reports, most of which are on specific aspects of radiation. Occasionally it publishes "fundamental recommendations" which describe the overall system of radiological protection. The most recent of these recommendations, in "ICRP Publication 103", were issued in 2007. On the basis of ICRP recommendations, international and regional bodies (the International Atomic Energy Agency (IAEA) and, for example, the European Commission's Euratom Programme) publish basic safety standards which are then used as the basis for national legislation.

    b. Other organizations play an important role in synthesizing the large scientific literature on radiobiology and the epidemiology of radiation risks. Chief amongst these are the United Nations Scientific Committee on the Effects of Atomic Radiation (UNSCEAR) and the reports on the Biological Effects of Ionizing Radiation (BEIR) produced by the National Research Council of the US National Academies.

9. Current Recommendations.

    a. The ICRP's recommendations are based principally on a derived quantity called **effective dose,** which is a weighted measure of the energy per unit mass deposited by different types of radiation in different tissues of the body (see paragraph 15 below). The unit of effective dose is called the "sievert" (abbreviated Sv).

    b. The average natural background effective dose experienced by an individual in the UK is 2.3 mSv/yr[1]. There is wide variation around this average.

    c. ICRP's system of protection intends to avoid injury through the effects of high doses and reduce the risks imposed by low doses or low dose-rates to an extent as low as reasonably achievable (ALARA). It does this through the application of three fundamental principles:

        i. Justification – any decision that alters radiation exposure should do more good than harm.

        ii. Optimization – numbers, likelihoods and magnitudes of exposures should all be kept as low as reasonably achievable (ALARA) taking into account economic and societal factors.

        iii. Application of dose limits – in planned exposures (other than medical exposures) the total dose to any individual (over their background exposure) should not exceed recommended limits.

    d. Justification requires consideration of all the consequences of a change in an activity involving radiation exposure. These include the risks associated with radiation and other risks, costs and benefits. Deliberations of justification are therefore much broader than radiological protection.

    e. Optimization is an ongoing qualitative and quantitative process, adapted to address each given situation, for both worker and public protection. Cost-benefit analyses are the main decision-aiding techniques in optimization procedures.

    f. For planned exposures the ICRP's effective **dose limit** for an individual member of the public is 1 mSv/yr and for an occupationally-exposed worker 20 mSv/yr. In practice, doses in planned situations will rarely approach dose limits because control is exercised by optimization of protection. Optimization of protection makes use of **dose constraints** applied to single sources. These are never greater than the pertinent dose limits of 1 mSv/yr for a member of the public and 20 mSv/yr for a worker.

[1] A millisievert or mSv is one thousandth of a sievert and a dose of a millisievert per year is written as 1 mSv/yr

g. For emergency and existing exposures the equivalent quantities are called **reference levels**. These are restrictions on doses below which optimization should be implemented. For naturally occurring radioactive materials and radioactive residues, the reference level is 1-20 mSv/yr according to the situation. For the specific case of the radioactive gaseous element radon, and its radioactive progeny, in the home or at work the reference level is of the order <10 mSv/yr.

h. For medical exposures the ICRP does not make numerical recommendations but instead emphasises the principle of justification: that the procedure should do more good than harm to the patient; that it should have a specified objective; and that these considerations should be specifically applied to each patient as an individual. The ICRP also emphasises the principle of optimization for medical exposures with the associated concept that doses should be as low as reasonably achievable (ALARA) consistent with achieving the clinical objective. Although there are no firm recommendations there are "diagnostic reference levels" which act as benchmark figures to help define good practice.

10. If these recommendations were under-precautionary, workers, members of the public and patients might be exposed to unacceptable health risks. Examples of situations where unacceptable risks might be incurred are:

a. Working conditions for workers in the nuclear industry.
b. Working conditions for medical and technologist staff in areas such as interventional radiography and cardiology, CT scanning, radiotherapy and positron emission tomography.
c. Working conditions in other industries with high exposures e.g. underground hard-rock miners, industrial radiographers.
d. Exposure to radon in the workplace or to the public in their homes.
e. Excessive exposure of the public from contaminated environments.
f. Excessive exposure of patients to diagnostic and therapeutic radiation.
g. Insufficient evacuation of the public after nuclear accidents.
h. Insufficient clean-up by nuclear workers after accidents or legacy operations.

11. These recommendations have profound effects upon costs for many industries and implications for medical practice. If the recommendations were over-precautionary these costs would be too high, or standards may even be unachievable. Examples of areas where unduly disadvantageous effects might be incurred are:

a. Working conditions for workers in nuclear and other industries (e.g. air-crew and miners).
b. Day-to-day functioning of existing nuclear industries (e.g. storage, zoning, transportation).
c. Commissioning of future nuclear facilities.
d. Patients may not undergo diagnostic procedures because of concerns about over-exposure.
e. Some medical practitioners (e.g. interventional cardiologists) may be limited in their specialist work.
f. Provision and staffing of clinical facilities using ionizing radiation.
g. Environmental clean-up of contaminated sites or installations (e.g. the legacy of operations at the Hanford and Sellafield nuclear sites).
h. Actions after accidents (e.g. Chernobyl and Fukushima).
i. There are known to be substantial psycho-social and other health costs linked to the evacuation of populations after nuclear accidents.
j. There is potential for substantial human cost in excess anxiety about exposure to ionizing radiation.

12. *Summary*. There is an international system of radiological protection which exists to protect people and the environment from the harmful effects of ionizing radiation. Natural science evidence is collected and summarised by various national and international bodies, recommendations are made at the international level and those recommendations are enacted as law and guidance by regional and national bodies.

DEFINITIONS AND UNITS

13. All ionizing radiation deposits energy as it passes through matter. The fundamental unit of dose from ionizing radiation is called the "**absorbed dose**", which is the amount of energy absorbed per unit of mass of material. It is measured in joules per kilogram using the SI unit the gray (1 Gy = 1 J/kg).

14. Ionizing radiation takes diverse forms which vary in their nature, their source, their distance of travel through materials and their biological effect for a

given absorbed dose (see Table 7 in the annotated bibliography). For the purposes of radiological protection some of the important dichotomies and variables are:

a. High-LET versus low-LET. Radiation that deposits energy densely along its “track” as it passes through matter is called “high-linear-energy-transfer” radiation (high-LET). Points of microscopic molecular alteration produced by a high-LET track, such as DNA strand breaks, are more likely to be spatially clustered and thus more difficult to repair than spatially separated lesions. For this reason high-LET radiation is more biologically damaging for the same absorbed dose than low-LET (i.e. sparsely ionizing) radiation.

b. Internal versus external exposure. The ionizing radiation from some common sources (e.g. alpha particles from naturally occurring radon) cannot penetrate the skin. However, a source of such radiation that is inhaled or ingested generates internal exposure and some tissues within the body are exposed. Assessing the risks from internal emitters is particularly difficult.

c. Partial versus whole-body exposure. Different organs and tissues within the body have different sensitivity to radiation, so the damage done by the same absorbed dose varies according to which organs or tissues were exposed to the radiation. Since absorbed dose is measured in joules per kilogram, the effect of a particular absorbed dose can only be interpreted in the context of how much and which parts of the body are exposed.

d. Rate of exposure. A dose can be acquired briefly in a single exposure (e.g. the brief exposure of the Japanese atomic bomb survivors), multiple exposures, or slowly accumulated from environmental or work-place exposure. The differences are described by “dose fractionation” (e.g. in radiotherapy when doses are separated in time) and “dose-rate”. The unit of measurement for dose-rate is Gy/min.

15. ICRP has devised the principal protection quantity of effective dose for the control of stochastic effects of radiation (cancer and hereditary effects). Because radiation types differ in their effectiveness per Gy of absorbed dose in causing stochastic effects, these differences are taken into account using radiation weighting factors: components of absorbed dose to individual tissues or organs are multiplied by these weighting factors to calculate **equivalent dose** (in Sv) to the tissues or organs. Equivalent doses to tissues or organs are then summed, multiplying them by tissue weighting factors that are simple representations of their fractional contribution to overall stochastic detriment, to obtain **effective dose** (in Sv). Effective dose provides a single metric for the summation of all doses, from external and internal sources that may irradiate the body uniformly or irradiate specific organs or tissues, for comparison with limits (or other control criteria) also set in effective dose. Effective dose is specifically formulated for use in the context of radiological protection for radiation exposures at low doses or low dose-rates, with defined weighting factors being recommended by the ICRP from its judgement based on scientific evidence available at the time. A unit effective dose is specified so that it is estimated to produce the same predicted risk to health as a unit absorbed dose of reference low-LET (e.g. gamma) radiation delivered uniformly to the whole body as a low dose or at a low dose-rate.

16. At high doses and regarding tissue reactions (deterministic effects, see paragraph 18a), organ or tissue doses are usually quoted in terms of absorbed dose in gray (Gy), and if high-LET radiations are involved, an RBE-weighted dose, RBE.D (Gy), may be used, where RBE is the relative biological effectiveness of the high-LET radiation for the specific effect under consideration.

17. Radioactivity is the transformation of an unstable atomic nucleus from one state to another during which radiation is emitted. Radioactive decay is measured in units of becquerel (Bq) where 1 Bq is the activity of one nuclear transformation per second. Dose delivered to the various organs or tissues of the body will depend on the location of the radionuclide, and the energy and tissue penetration of the emitted radiation. Thus, for example, external exposure from a radionuclide emitting gamma rays can lead to uniform whole-body irradiation (essentially delivering the same dose to all organs), while internal exposure from an inhaled radionuclide emitting alpha particles (for instance, radon) may irradiate principally one organ or tissue region within that organ (for instance, airways of the respiratory tract from deposited radon progeny). Some radionuclides form part of radioactive decay chains; that is, the elements formed by successive transformations are also radioactive and in turn decay emitting radiation.

For example, radon-222 decays through a series of solid elements, including polonium isotopes (radon progeny) that are alpha particle emitters and are responsible for 'radon-induced' lung cancer.

18. The nature of the damage done to organs and tissues by ionizing radiation is different at different doses. There are two broad categorizations.
   a. "Harmful tissue reactions" (previously called "deterministic effects"; the name changed because of the development of response modifying compounds which can reduce or delay the pre-determined effect or reaction after a given dose) occur mostly after high doses. Such damage will be experienced by all exposed individuals above a certain absorbed dose to the organ, tissue, or population of cells, and the severity of these effects then increases with increasing dose. The underlying mechanism is mainly cell killing; examples include gonadal sterility and suppression of haematopoiesis, as well as other functional tissue injury at later times. Massive cell killing, particularly of the most sensitive and critical stem cell populations, can be sufficient to cause death as a result of, for example, damage to the haematopoietic system or loss of integrity of the intestinal epithelium. There are threshold doses below which these kinds of tissue reaction do not occur. **[C$_{ons}$]**
   b. At all doses, ranging from high doses down to low doses, the term "stochastic effects" is used to describe damage that leads probabilistically to effects (largely cancer, but also hereditary effects) that will not occur in all exposed individuals. The underlying mechanism is non-lethal modification of structures within the cell (largely DNA damage), and the effects are cancer in the exposed individual and hereditary changes to the descendants of exposed individuals (these latter are seen in animal experiments, but not conclusively observed in humans). Here, the probability of experiencing ill-effects, but not the severity of the effect, is determined by the dose received by organs and tissues. **[C$_{ons}$]**
   c. Whether or not there is a threshold dose below which the probability of stochastic effects becomes zero is a matter of debate. **[N$_{oco}$]**
   d. The assumption that risk is proportional to dose (the LNT model) is central to the operation of the radiation protection system. It is a pragmatic approach to a practical problem and most scientists working in the field (but not all) view it as prudent.
   e. Some radiogenic diseases (particularly cataracts and cardiovascular disease) do not fall neatly into the harmful tissue reactions versus stochastic effects dichotomy, and there is substantial debate as to whether such health effects are produced by low-level exposure. **[N$_{oco}$]**

19. Not all people are equally sensitive to damage from ionizing radiation. The factors that govern individual sensitivity to radiation are not fully understood.
   a. Children generally have higher sensitivity than adults. **[C$_{ons}$]**
   b. There are certain known rare genetic disorders that are known to increase the probability of adverse effects of radiation exposure. **[C$_{ons}$]**
   c. It is assumed that some variation in radiosensitivity exists in the general population, but the extent of this variation is not properly understood. **[N$_{oco}$]**
   d. Some behaviours increase the risk of damage from radiation (e.g. smoking greatly increases the absolute risk of lung cancer from radon exposure, see paragraph 88b below), but there is an incomplete understanding of the nature of the interactions between radiation and other risk factors. **[C$_{ons}$]**

20. *Summary*. The absorbed dose of radiation is quantified in gray (Gy) and is the amount of energy deposited in joules per kilogram. Equivalent dose and effective dose use weightings of absorbed dose and are described in sievert (Sv). For the purposes of radiological protection at low-level exposure, recommendations regarding stochastic effects are issued using effective dose in sievert. Ill-effects of radiation are divided into two broad types: "harmful tissue reactions" at higher doses and "stochastic effects" (such as cancer) across all doses including lower doses.

BACKGROUND EXPOSURE AND UNCERTAINTIES AT LOW DOSE

21. In the UK the average annual effective dose from natural background radiation is 2.3 mSv and about half of this is from radon (a naturally occurring radioactive gas) and its radioactive progeny. The global annual average from natural sources is 2.4 mSv. Medical procedures are a source of substantial and growing additional exposure – an additional 0.44 mSv/yr in the UK, and an additional

3 mSv/yr in the US. The ICRP's recommended annual effective dose limit for the public in planned situations is for additional exposures over and above these background and medical exposures. At 1 mSv in a year, it is about half the global average annual dose received from natural background radiation. **[C_{ons}]**

22. Background radiation varies greatly with location, largely driven by underlying geology, but also by other factors like altitude. At finer spatial scales even individual houses can have different radon gas concentrations because of geological features and the way they are built. Indoor radon levels across Europe have been mapped at a resolution of 10 km x 10 km (Figure 1), revealing that even within Europe this source of radiation varies by two orders of magnitude. **[C_{ons}]**

23. Because of intense interest in the risks posed by ionizing radiation, there are many epidemiological studies of the relationship between risk of disease and dose of radiation. Many of these are described in paragraphs 41-99. Figure 2 summarises the results of some of the bigger and statistically better-powered studies of solid cancer, leukaemia and lung cancer risk. The figure shows how the risks of various cancers are clear at high doses and reduce as dose decreases. As lower and lower doses are considered, larger and larger well-designed epidemiological studies are needed to distinguish reliably between low risk and no risk. **[C_{ons}]**

24. Knowledge of the radiobiological mechanisms that govern the disease risks posed by low-level exposure to radiation is incomplete, so risks from low doses or low dose-rates must be inferred by extrapolation from the risks obtained from epidemiological studies of higher doses delivered briefly but informed by what is known from experimental systems. For epidemiological data generated by studies at low doses the wide confidence intervals on the estimated risks are compatible with a wide range of different dose response models. Figure 3 presents six potential risk models. **[P_{rojn}]**

    a. Linear no-threshold (LNT) assumes that the risk is directly proportional to dose.

    b. LNT with a dose and dose-rate effectiveness factor (DDREF) >1 assumes that risk is proportional to dose, but that at low doses or low dose-rates the risk per unit dose (and hence the slope of the response) is smaller than that measured at acute moderate-to-high doses. The DDREF encompasses a low dose effectiveness factor (LDEF) and a dose rate effectiveness factor (DREF), both currently assumed to be 2 although it is recognised that these are separate entities. This is the model currently used by the ICRP with DDREF = 2.

    c. LNT is not necessarily the most conservative assumption. Hypothetically, if a small subset of the exposed population were particularly sensitive to radiation risk, or if there were a "bystander" effect (whereby cells not directly traversed by radiation are affected by neighbouring cells that are hit) that became saturated at higher doses, the expected shape of the dose-response curve could be non-linear with an initial higher slope and then a reduced slope.

    d. Models of the interaction or competitive-repair of DNA damage as the drivers of risk can generate a non-linear curve with an initial lower slope and then an upward curve. Other biophysical mechanisms can also account for such shapes.

    e. Models with a threshold assume that there is a dose below which there is no excess risk.

    f. The concept of hormesis is that very low doses and very low dose-rates are beneficial to health and protective with regard to subsequent exposure.

25. *Summary*. Across the world the average effective dose from natural background radiation is 2.4 mSv/yr. Large epidemiological studies can be used to estimate the health risk of higher doses and, through statistical calculation of confidence intervals, infer that risks are greater than zero. But at doses in the range of the natural background, even the largest epidemiological studies have substantial difficulties in reliably distinguishing between low risk and zero risk. Radiobiological knowledge of relevant processes following low-level exposure is incomplete and therefore point estimates for low dose or low dose-rate risks above the background are inferred by extrapolation from the results of epidemiological studies at higher doses. Several different models can be used for such extrapolation and most are largely consistent with the low-level exposure data available.

ACUTE HIGH DOSE EXPOSURES

26. At high doses (1 Gy or above) the damage done to organs and tissues is relatively easy to recognise. For this type of exposure, dose is expressed as absorbed dose in gray.

27. Mortality and morbidity is caused by stochastic effects such as cancer and tissue reactions such as severe damage to the central nervous system (CNS), the gastrointestinal system, the heart, the lungs and the haematopoietic system, over different ranges of dose and latency times. **[C_{ons}]**

28. Table 1 lists deterministic causes of mortality and morbidity after acute high dose irradiation. For both mortality and morbidity the table records the impact of a whole-body dose of low LET radiation delivered in a single brief exposure. For morbidity the dose recorded is the minimum threshold dose above which >1% of a healthy adult population would experience the ill effect. Above these estimated threshold doses incidence and severity rise. **[C_{ons}]** At doses above about 0.5 Gy, cataracts and circulatory disease effects are deterministic but late acting. Whether or not doses below about 0.5 Gy cause cataracts **[N_{oco}]** and circulatory disease **[N_{oco}]** are topics of current study and debate.

29. Tissue reactions in the embryo or fetus depend upon the stage of gestation when exposure occurs as well as the dose. Prior to implantation (days 0-9) the main effect is lethality to the embryo. Malformations are mainly induced during the period of major organogenesis. Japanese A-bomb survivors who were exposed in utero showed a clear excess of mental retardation and a generalised decrease in IQ; however, these deficits were only observed in children who had been exposed during weeks 8 – 25 of gestation. The same population exhibited an excess of microcephaly amongst those exposed during the first and second trimesters and reduced stature after exposure in any trimester. They also exhibited stochastic effects described at paragraph 50. **[C_{ons}]**

30. Even at moderate or high doses no statistically significant excess hereditary effects have been seen in the offspring of people who were exposed prior to conception. Nevertheless estimated hereditary risk is included in the ICRP recommendations, because of the clear evidence from large-scale mouse studies that radiation can cause hereditary effects in mammals. **[C_{ons}]**

31. *Summary.* High doses are described in units of gray. With a whole-body acute dose of >15 Gy, death is certain within 5 days. With a whole-body acute dose of 2.5-5 Gy, without good medical care, death due to bone marrow damage may follow within 2 months in around 50% of healthy adults exposed. With a whole-body acute dose of 1 Gy, without good medical care, death due to bone marrow damage may follow in about 10% of those exposed. Doses above about 0.5 Gy will depress blood-forming processes over the coming week and cause a range of other morbidities including erythema, epilation and sterility. Cataracts and damage to the circulatory system that may become apparent many years later are also caused at doses above about 0.5 Gy; whether or not lower doses cause cataracts and circulatory disease is a topic of ongoing study and debate. Even at high doses no statistically significant excess of hereditary effects have been seen in the offspring of people who were exposed prior to conception, although animal experiments do show such effects and imply that they may occur at a very low frequency in humans.

Table 1. Mortality and morbidity after acute high dose irradiation (harmful tissue reactions or deterministic effects).

Dose in Gy	Consequence
**Mortality: after acute low LET uniform whole body exposure**	
>15	Death via nervous system damage, in 0-5 days
5-15	Death via gastrointestinal tract damage, in 7-20 days or via lung and kidney damage within 60-150 days
3-5	Without medical care, death of 50% of an exposed healthy adult population via haematopoietic syndrome, within 60 days
1-2	Without medical care, death of ~ 10% of patients via haematopoietic syndrome, in 30-60 days
**Morbidity: early effects in specific tissues**	
6-7	Acute pneumonitis, 1-3 months onset
6	Erythema reaction, 10 days onset
6	Permanent male sterility, 3 weeks onset
4	Temporary epilation, 3 weeks onset
3	Permanent female sterility, <1 week onset
2	Early transient erythema, 2-24 hr onset
1	Vomiting, 1-24 hr onset
1	Haematopoietic syndrome onset, 1 hour to 2 days onset
0.5	Depressed haematopoiesis, 3-7 days onset
0.1	Temporary male sterility, 3-9 weeks onset
**Morbidity: later effects**	
1-2	Cognitive defects, onset after several years
0.5	Cataracts or circulatory disease many years after acute or fractionated exposure
0.1-0.2	Cognitive defects in infants, onset after several years

LOW DOSE EXPOSURES

32. The primary health effect of low or moderate doses of ionizing radiation is an increased risk of cancer in the exposed individual. **[Cons]**

33. There is a view that the burden of non-cancer mortality (in particular cardiovascular disease) may be of a similar magnitude, although this remains under debate. **[Noco]** Whether cataracts can be induced by low-level exposure is a subject of current investigations. **[Noco]**

34. Cancers are common in human populations and (in almost all cases) it is not currently possible to distinguish whether an individual case of cancer is due to radiation or some other cause. For this reason it is necessary to study a large number of people in order to detect additional radiogenic cancer cases with confidence. This is particularly true if the dose is small. Theoretically, under a linear no-threshold model, a population of 1 billion would probably be required to detect a statistically significant overall cancer risk from an exposure of 1 mSv above background levels. Such a study would not be feasible even if uncertainties in dosimetry and confounding factors were not present. For some especially sensitive outcomes, such as leukaemia following exposure in early childhood, the number of individuals required will be smaller, but will still number in the tens of thousands. **[Cons]**

35. Most cancers tend to occur later in life and radiogenic cancers tend to occur long after exposure, although leukaemia and thyroid cancers can sometimes appear within a few years of exposure. Well-powered studies therefore have to be carried out over protracted periods as well as including many people. **[Cons]**

36. Many studies in radiation epidemiology have low power to detect radiogenic disease at low doses or low dose-rates. Such studies would be expected to generate very variable results in which only unusually large effects achieve statistical significance and many of those will be false positives due to chance or sampling variation. This is exactly the pattern observed at low doses. **[Cons]**

37. Epidemiological associations can be expressed in a number of ways. Common measures used in radiation epidemiology are:

   a. Excess relative risk (ERR) – the proportional increase in the rate of disease in the exposed population. This is calculated as the ratio of the excess rate of disease in the exposed population to the rate of disease in an equivalent but unexposed population.

   b. Excess absolute risk (EAR) – the difference between the rate of disease in the exposed population and the rate of disease in an equivalent but unexposed population.

   c. Odds ratio (OR) – the odds of disease occurrence in an individual in the exposed group divided by the odds of disease occurrence in an individual in an equivalent but unexposed group.

   d. Other measures are also used, including relative risk (RR=ERR+1), hazard ratio (HR), and standardised incidence/mortality ratio (SIR/SMR).

   e. Each of these metrics has advantages and disadvantages that depend on the circumstances. In this restatement, where the data are available, we generally express risks as the ERR with a 95% confidence interval (CI). We chose ERR because it is often the most appropriate measure when comparing results from diverse studies and is commonly available. ERR must always be interpreted in the light of the magnitude of the underlying risk. ERR per gray is a useful measure for our purpose of comparing different studies. Our use of it recognises the uncertainties associated with the application of an LNT dose-response relationship.

38. Table 2. Effective doses received from common sources of exposure. **[Cons]**

Source of exposure	Dose in mSv
Dental x-ray	0.005
Consuming 100g of Brazil nuts	0.010
Chest x-ray	0.014
Transatlantic flight	0.08
UK annual average radon dose	1.3
CT scan of the head	1.4
UK average annual radiation dose (excluding medical diagnostics)	2.3
UK average annual radiation dose (including medical diagnostics)	2.7
USA average annual radiation dose (excluding medical diagnostics)	3.2
USA average annual radiation dose (including medical diagnostics)	6.2
CT scan of the chest	6.6
Average annual radon dose to people residing in Cornwall	7.8
CT scan of the whole spine	10.0

39. Epidemiological studies have followed individuals exposed in the Japanese atomic bombings, the Chernobyl nuclear accident, the Fukushima Dai-ichi nuclear accident, at work, through their environment and through medical procedures.

40. *Summary*. The primary ill-health caused by low to moderate doses of ionizing radiation is cancer, although the possibility of non-cancer effects (particularly cardiovascular disease) is of increasing concern. Very large studies would be required to detect the ill-effects of doses of around 1 – 10 mSv. Doses of this size are routinely encountered – for example, from natural background radiation and medical diagnostic exposures. Radiation epidemiology is primarily informed by studies that compare individuals with varying levels of radiation exposure.

THE JAPANESE LIFE SPAN STUDY (LSS)

41. In August 1945, two atomic bombs were detonated above the Japanese cities of Hiroshima and Nagasaki. In 1950 a study was set up to follow the long-term after-effects of radiation on survivors, and this cohort study, known as the Life Span Study, or LSS, now forms the key reference population for the radiological protection community. This cohort study is of high quality for evaluating radiation risks because of its large size; broad range of ages at exposure; completeness, duration and fidelity of follow up; and high quality dose estimation over a wide range of doses. **[C_{ons}]**

42. The study commenced in 1950, 5 years after the bombings and some (but far from all) commentators consider that this delay might have led to bias because relatively unhealthy exposed people might have died before then, due to the generally difficult living conditions after the war, causing a "healthy survivor" effect. **[N_{oco}]**

43. The original cohort consisted of around 120,000 people: 55,000 who were within 2.5 km of the blasts; 38,500 city-, age- and sex-matched controls who were present in the cities but further from the blasts; and 26,500 who were not in the city at the times of the bombings. The mean dose in the group within 2.5 km of the explosions was estimated to be around 200 mGy. Mortality and cancer incidence are assessed in the survivors at regular intervals. **[C_{ons}]**

44. Because dose could not be accurately estimated for 7,000 people and those not in the city were excluded from most analyses of the study, there were approximately 86,500 individuals in the LSS with a reliable dose estimate. As of 1 January 2004 (the end of follow-up for the most recently published major review of mortality data) 50,620 of those had died. Approximately 1,000 of those deaths are attributed as excess deaths due to radiation exposure during the bombings. **[C_{ons}]**

45. The Adult Health Study (AHS) is a study of a sub-cohort of the LSS in which clinical examinations of about 10,000 survivors are conducted and blood samples collected every two years. The AHS investigates the risk of non-cancer diseases and physiological changes.

46. An excess of cases of leukaemia among highly exposed survivors started to become apparent from clinical observations in the late 1940s. Excesses of other cancers were reported from the LSS in later years, and now radiation-related excesses are apparent for most types of cancer, although not for all types – for example, there are no significant excesses of pancreatic or rectal cancers. **[C_{ons}]**

47. Table 3. Current estimates for mortality ERR from the LSS. **[C_{ons}]**

**Disease**	**ERR/Gy**	**95% CI**
All solid cancers (based on dose to colon)	0.47	0.38 to 0.56
Leukaemia (ERR at 1 Gy, not per Gy) (based on dose to bone marrow)	3.10	1.80 to 4.30

48. Cataract incidence has also been linked to radiation dose, and – because of recent studies at longer follow-up times showing a lower dose threshold – this is coming to be viewed as possibly a stochastic effect rather than a harmful tissue reaction which one might expect to see only at higher dose. **[N_{oco}]**

49. A follow-up of survivors exposed *in utero* has detected an excess risk of the incidence of solid cancers, but no evidence of increased childhood leukaemia **[C_{ons}]** This could be due to cell-killing by moderate and high doses to the haematopoietic system.

50. Studies of children exposed *in utero* found clear evidence for severe mental retardation amongst those exposed at weeks 8-25 post-conception, with the effect being greatest for those exposed at weeks 8-15 post-conception. There was also a generalised downward shift in IQ for those exposed in weeks 8-

25, but there was no evidence of a radiation effect on intelligence for those exposed before week 8 or after week 26 of gestation. **[C_{ons}]**

51. Studies of over 75,000 individuals have found no statistically significant radiation-associated adverse hereditary effects in the offspring of Japanese atomic bomb survivors who were conceived after their parents were exposed. For example, a study with a median of 54 years of follow up reported a hazard ratio (the hazard in the exposed group divided by the hazard in the unexposed group, which has a baseline value of 1) at 1 Gy of 0.89 (95% CI: 0.69 to 1.15) for maternal gonadal radiation exposure and risk of cancer mortality, and a hazard ratio of 0.82 (95% CI: 0.61 to 1.08) for paternal gonadal radiation exposure and risk of cancer mortality. **[C_{ons}]**

52. *Summary.* The study of survivors of the atomic bombings of Japan (the LSS) is the largest and longest study of risks from ionizing radiation. It is treated as the "gold standard" in the sense that the results of other studies are compared with its results. Its headline results are that at 1 Gy (dose to the colon) the risk of mortality from solid cancer is raised by 50% and at 1 Gy (dose to the red bone marrow) the risk of mortality from leukaemia is quadrupled. Note that the excess relative risk quoted here is different from the nominal excess absolute lifetime risk coefficient for cancer of 5.5% per Sv derived by the ICRP and used in optimization calculations. Excess relative risk (the proportional increase in risk) is only meaningful in the context of the underlying risk in an unexposed population. So, for example, in the LSS to 2003 there were 50,620 deaths, of which 10,929 were from solid cancers, and 318 from leukaemia. Thus, even though the ERR at 1 Gy is much higher for leukaemia than for solid cancer, around 525 of the solid cancer deaths and only around 105 of the leukaemia deaths are estimated to be radiation-associated. Large studies of individuals conceived to parents who were survivors of the atomic bombings find no statistically significant adverse effects.

THE CHERNOBYL NUCLEAR POWER PLANT ACCIDENT

53. In April 1986 an explosion, a fire and severe damage to fuel in a nuclear reactor at the Chernobyl power plant in Ukraine released large quantities of radioactive material into the atmosphere. **[C_{ons}]**

54. Exposure ranged from high whole-body doses leading to acute radiation sickness (134 people including 28 fatalities) in early emergency workers, high thyroid and moderate whole-body exposures in the local population (hundreds of thousands of people received whole-body doses of around ten to several hundred mGy), whilst hundreds of millions of people across Europe were exposed to additional doses of less than 1 mGy. **[C_{ons}]**

55. In the local population, tens of thousands of children received thyroid doses of greater than 1 Gy, mainly due to drinking milk heavily contaminated with radioactive isotopes of iodine. **[C_{ons}]**

56. By 2005 there were around 50 deaths that could be unequivocally attributed to the disaster: the 28 early fatalities above; some, but not all of 19 acute radiation sickness survivors who have since died; and 15 children who had died from thyroid cancer. **[C_{ons}]**

57. There is a well-documented excess incidence of thyroid cancer amongst people who were highly exposed as children with around 6,000 cases detected by 2005. One recent study reports an ERR/Gy of 1.91 (95% CI: 0.43 to 6.34) **[C_{ons}]**. There is no matching convincing evidence of any excess risk of thyroid cancer among those children less exposed to fallout. **[N_{oco}]**

58. Studies of leukaemia risk amongst workers have frequently been limited by low statistical power, dose reconstruction uncertainties and absence of case verifications. Studies of a large cohort of Ukrainian cleanup workers found an ERR/Gy for leukaemia incidence of 1.26 (95% CI: 0.03 to 3.58) – in line with what would be expected from the LSS. **[E_{mco}]** Some, but not all, studies of leukaemia incidence amongst Chernobyl workers have found significant results for chronic lymphocytic leukaemia (CLL) which is generally considered to have a low sensitivity to induction by radiation, suggesting study problems or a hitherto unacknowledged radiogenicity for CLL amongst these cohorts. **[N_{oco}]**

59. There is no consistent evidence of excess risk of leukaemia for those exposed *in utero* or as children, or for the general adult population. **[C_{ons}]**

60. For solid cancers the picture is less clear – partly because of the absence of suitable cohorts. A number of studies of highly exposed workers found no relationship between radiation dose and incidence of solid cancers. However, a recent study of 67,500 Russian recovery workers found a significantly elevated risk of solid cancer incidence with an ERR/Gy of 0.47 (95% CI: 0.03 to 0.96). Some

studies have reported an increased risk of thyroid cancer in workers. However, surveillance bias is possible in these studies of recovery workers. Studies of residents of highly contaminated areas have found no excess of solid cancers. **[E$_{mco}$]**

61. A relatively small study of breast cancers in the most contaminated areas of Belarus and Ukraine found a significant increase in risk for the years 1997-2001, but this result was not replicated in a follow-on study. **[N$_{oco}$]**
62. There is a plethora of small studies with low power, some of which describe excess risk of solid cancers attributable to the Chernobyl accident, but these studies are very difficult to interpret reliably. **[N$_{oco}$]**
63. Thus far no other type of cancer has been shown unequivocally to be increased in people exposed to radiation in the environment from the accident. **[N$_{oco}$]**
64. There is emerging evidence for an increase in benign thyroid adenomas as well as some other thyroid non-cancer disorders in adolescents and children exposed to Chernobyl fallout. **[E$_{mco}$]**
65. There is evidence of excess risk of cataracts in recovery workers **[E$_{mco}$]**, and some evidence for an elevated risk of the incidence of circulatory disease. **[N$_{oco}$]**
66. Although there is, in general, little evidence for an excess risk of congenital malformations associated with Chernobyl exposure, a high rate of neural tube defects has been reported from northern Ukraine although the interpretation is unclear. **[N$_{oco}$]**
67. Some risk projections for the expected numbers of additional cancers across Europe arising from the Chernobyl accident have been published. These additional cancers account for around 0.01% of total expected cancers, and would not be detectable in studies using national cancer statistics. **[C$_{ons}$]**
68. The most significant public health consequences of the Chernobyl accident are likely to be social and mental health effects with large and sustained consequences, particularly with regards to depression. **[C$_{ons}$]**
69. Studies of health effects resulting from the Chernobyl accident are complicated by the substantial background effects arising from the socioeconomic turmoil that followed the collapse of the USSR. There are considerable difficulties in distinguishing between the causes of health effects in the former USSR.
70. *Summary*. A number of early emergency workers at the accident at the Chernobyl nuclear power plant received high doses which produced tissue reactions and 28 early deaths. The long-term health impacts are contested. There is consensus on two major health impacts: thyroid cancers caused by high levels of exposure of children to radioactive iodine, and ill-effects to mental health caused by widespread fear of potential risks and social disruption. There is emerging evidence on the risk of leukaemia amongst recovery workers and those risks are broadly in line with what is expected from the LSS. At present, there is little convincing evidence of other radiation-associated effects in recovery workers or the wider public.

THE FUKUSHIMA DAI-ICHI NUCLEAR POWER PLANT ACCIDENT

71. In March 2011 a magnitude 9 earthquake off the east coast of Japan caused tsunamis that led to over 15,000 deaths and over 2,500 missing people. At the time of the earthquake, 3 of the 6 reactors at the Fukushima Dai-ichi nuclear power station were operating, and these shut down as planned. However, the tsunamis damaged equipment and flooded the emergency generators leaving the site without electrical power and the means to cool the recently operational reactors, the fuel of which was then seriously damaged, leading to a severe nuclear accident. A 20 km radius area around the station was evacuated. **[C$_{ons}$]**
72. Several thousand emergency and recovery workers were exposed to radiation with most exposed to less than 10 mSv. Of those workers involved in the emergency in the first few days of the accident, 6 received effective doses in excess of 250 mSv, mainly due to high thyroid doses (>1 Gy) from inhaled radioiodine. **[C$_{ons}$]**
73. There were no cases of acute radiation sickness after the accident. Although 5 workers died at the site, their deaths were caused by heart disease and accidents, not by radiation. One worker, who received 16 mSv while involved in recovery work during 2012-13, has developed leukaemia and is eligible for compensation as a consequence, but a causal link between this low dose and the disease is unlikely. **[C$_{ons}$]**
74. Additional lifetime effective doses to members of the public (whether in the evacuated districts or in nearby districts that were not evacuated) are

estimated to be around 10 mSv for adults and about 2-fold higher for children and infants. These are expected to be overestimates because of assumptions that were made with inadequate data. **[C_{ons}]**

75. In the most affected district, doses to the thyroid for infants of up to 80 mGy were initially estimated. Actual measurements of thyroid doses in some evacuees gave a median value of 4.2 mGy in children; 98.8% of the measured children had doses of <15 mGy. **[C_{ons}]**

76. Owing to the low doses and small number of people exposed, no general radiation-related increase in ill-health is expected to be discernible. However, it is less clear whether an increased incidence of thyroid cancer among those exposed as children may become apparent, but because the doses were substantially lower than those after the Chernobyl accident, it is expected that there will no discernible increase in thyroid cancer. **[C_{ons}]**

77. A programme to screen the thyroids of all residents of Fukushima prefecture below 19 years old using ultrasound was initiated a few months after the accident. That study detected 113 confirmed or suspected thyroid cancers in its first few years – many-fold more than would be expected from Japanese cancer registry data. However, this excess is attributable to the large and sensitive screening effort and not to an effect of radiation exposure. **[C_{ons}]**

78. The major health impacts have been non-radiation health effects. For example excess deaths amongst evacuated hospitalized patients and senior citizens' homes, and widespread, ongoing psycho-social ill-health. **[C_{ons}]**

79. Summary. The Fukushima Dai-ichi nuclear power plant accident has caused substantial ill-health through the effects of the evacuations, continued displacement and fear of radiation. It is unclear if there will be a detectable excess in thyroid cancer in the coming years. No other discernible increase in ill-health attributable to radiation exposure is expected in either emergency and recovery workers or members of the public.

STUDIES OF WORKERS EXPOSED TO RADIATION

80. Studies of those exposed in the workplace give direct evidence of the impact of protracted exposures to low-level external radiation. Some workers, such as underground hard-rock miners exposed to radon and its radioactive progeny, also receive doses from internal emitters.

81. Those who work in the nuclear industries have been the subject of a number of studies.
  a. Because nuclear workers' exposure to radiation is monitored, measuring doses for external exposure is comparatively straightforward. Table 4 summarises results from 5 discrete and relatively large studies of nuclear workers for solid cancer and leukaemia. Equivalent results for the LSS are given for comparison. In the International Nuclear Workers' Study (INWORKS), when analysis is restricted to those whose total accumulated dose is 100 mGy or less, the ERR/Gy for solid cancer mortality is marginally significant at the 90% level. Despite disparity between dose-rates, results from the worker studies and the LSS are in broad agreement. **[C_{ons}]**
  b. There are fewer published analyses of the risk from internal doses (apart from the large literature on radon). Studies of the workers of the Mayak weapons plant (in the Russian Federation) describe increased risks from inhalation of high levels of plutonium. For example, for lung cancer in males the estimated ERR/Gy is 7.4 (95% CI: 5 to 11) based on 446 deaths. The Mayak workers' risks from external radiation are included in Table 4 for comparison. **[C_{ons}]**
  c. Studies of non-cancer disease risk such as circulatory disease in nuclear workers have produced mixed results. Because such disease is common compared with cancer, even a small ERR would lead to a large number of extra deaths. It is not yet possible to draw a confident conclusion on low-level radiation exposure and circulatory disease risk. **[N_{oco}]**

82. Table 4 lists ERR/Gy for solid cancers and leukaemia following external radiation exposure, estimated from some of the larger studies of workers in nuclear industries.

Table 4. ERR/Gy for solid cancers and leukaemia following external radiation exposure, estimated from some of the larger studies of workers in nuclear industries.

Study	N Total	Mean dose (mGy)	Mean dose per year (mGy/yr)	ERR/Gy (confidence interval)	# deaths
**Solid cancer mortality**					
LSS male survivors exposed ages 20-60 years				0.32 (95%, 0.01 to 0.50)	3,246
INWORKS international nuclear workers*	308,297	20.9**	1.7	0.47 (**90%**, 0.18 to 0.79)	19,064
*INWORKS (<100 mGy)**				*0.81 (**90%**, 0.01 to 1.64)*	*17,814*
Japan nuclear workers *	200,583	12.2		0.20 (95%, -1.42 to 2.09)#	2,636
Chernobyl clean-up workers	67,568	132.0	132	0.58 (95%, 0.00 to 1.25)	2,442
US nuclear power plant workers	53,698	25.7		0.51 (95%, -2.01 to 4.64)	368
Mayak nuclear workers	25,757	354.0		0.12 (95%, 0.03 to 0.21)##	1,825
**Leukaemia excluding CLL mortality**					
LSS male survivors exposed ages 20-60 years				1.40 (90%, 0.10 to 3.40) ++	83
INWORKS international nuclear workers	308,297	15.9+	1.1	2.96 (**90%**, 1.17 to 5.21)	531
Japan nuclear workers	200,583	12.2		-1.93 (95%, -6.12 to 8.57)	80
US nuclear power plant workers	53,698	25.7		5.67 (95%, -2.56 to 30.4)	26
Mayak nuclear workers	22,373	390.0		3.57 (90%, 1.55 to 8.22)	56

*result is for all cancers excluding leukaemia, rather than for solid cancers

** mean colon dose

#excluding alcohol-related cancers

adjusted for plutonium exposure and excluding lung, liver and bone cancers

+ mean red bone marrow dose

++ based on the linear term of the linear-quadratic model

83. Excess skin cancer and leukaemia amongst radiologists was the first evidence of elevated cancer risks following radiation exposure. **[C_{ons}]**

a. Analysis of cancer risks amongst medical workers has to allow for great reductions in their exposure over the years. The earliest cohorts worked in a time (the 1920s) when the radiological protection standard for occupational exposure was to restrict exposure to less than the equivalent of 700 mSv/yr. A review of 8 large studies (totalling 278,000 workers) of radiologists and radiological technicians found excess risks of leukaemia, cancers of the skin and, in women, breast cancer amongst early cohorts and no excess cancer risk in more recent workers. That study cautions that recent workers are still young and will need to be followed as they age and enter those age groups in which background cancer risks are higher. **[C_{ons}]**

b. There is evidence of excess risk of circulatory disease mortality in early cohorts of US radiological technicians, when compared with cohorts who started work after 1960. Studies of circulatory disease risk in other cohorts have given mixed results. **[N_{oco}]**

c. For cataract risk there is mounting evidence of risks of lens opacities for medical specialists who conduct interventional procedures whilst using X-ray imaging without protective eyewear. **[E_{mco}]**

84. During the first half of the 20th Century workers (mostly female) employed to apply radium-based luminous paint to instrument dials inadvertently ingested large quantities of the radium radioisotopes ^{226}Ra and ^{228}Ra. Radiation dose estimates can only be crude but for the US workers are in the region of 10 Gy to the skeleton, where radium deposits. Clear excesses of bone cancers were observed in US and (less so) in UK cohorts, and the US workers had an excess of head cancers thought to be due to radon from the decay of ^{226}Ra **[C_{ons}].** The cohorts also experienced excess breast cancer (possibly due to external radiation from the paint pots) **[N_{oco}]**, but not of leukaemia. **[C_{ons}]**

85. Aircrew are exposed to a few mSv/yr of additional cosmic radiation. Studies of their cancer risks have revealed excess risk of malignant melanoma when compared with the general population, which is likely to be due to behavioural factors other than their additional exposure to ionizing radiation. **[E_{mco}]**

86. Underground hard-rock miners are exposed to radon and its radioactive progeny – a source of internal alpha-emitters when inhaled – delivering radiation doses mainly to the upper lung. Occupational exposure to radon is measured in terms of the length of time an individual is exposed to a certain air concentration of radon progeny, the so-called "working-level-month" or "WLM".

a. It had long been known that such miners had increased risk of lung cancer and extensive cohort studies have quantified this risk. A reasonable summary estimate is an ERR per 100 WLM of 0.5. **[C_{ons}]**

b. Studies of other cancers (including leukaemia) in underground miners mostly find no evidence of a relationship between exposure to radon and cancers other than lung cancer. A large study of uranium miners found a positive non-significant association (ERR of 2.18, 95% CI: -0.41 to 6.37) between leukaemia mortality excluding chronic lymphocytic leukaemia with cumulative radon exposure. **[N_{oco}]**

c. The risk of cardiovascular disease does not currently appear to be related to exposure to radon. **[C_{ons}]**

87. *Summary.* Workers in the nuclear industries often have both external and internal radiation exposure. Their risks from external doses for solid cancer and leukaemia are consistent with those observed in the LSS even though their doses are accumulated at low dose-rates over many years. In the International Nuclear Workers Study (INWORKS), even amongst those who have total accumulated doses below 100 mGy, the risk of mortality from solid cancer is consistent with the LSS estimate (although the confidence intervals are wide). Radiologists and radiation technicians who worked during the early years have increased risks of leukaemia, skin cancer and, for women, breast cancer. More recent cohorts (from an era of lower doses to workers) have not yet displayed excess risks, but are still young. Cataract risk may be increased in medical workers who use X-ray imaging to guide interventions. Underground hard rock miners have an elevated risk of lung cancer roughly in proportion to their exposure to radon gas and its radioactive progeny.

ENVIRONMENTAL EXPOSURE

88. On average, about one half of the effective dose from natural sources is from the inhalation of radon whilst indoors.

a. Three analyses of pooled data from different geographic regions indicate a significant association between exposure to residential radon and the risk of lung cancer. Residential radon concentration is described using Bq/m^3. Those studies are based in: Europe, ERR per 100 Bq/m^3 = 0.08 (95% CI: 0.03 to 0.16); North America ERR per 100 Bq/m^3 = 0.10 (95% CI: -0.01 to 0.26); and China ERR per 100 Bq/m^3 = 0.13 (95% CI: 0.01 to 0.36). **[C_{ons}]**

b. For people who have never smoked the data are less clear. The largest of the pooled studies finds approximately the same excess relative risk for lung cancer in never-smokers as in smokers. Since the underlying risk of lung cancer is around 25-fold higher for smokers than for lifelong non-smokers, the increase in absolute risk from radon is much greater for smokers. Other smaller studies tend to find a positive association between exposure to residential radon and the risk of lung cancer for never smokers, but this association is frequently not statistically significant. **[E_{mco}]**

c. A study of domestic radon and childhood cancer in Denmark found an association between acute lymphoblastic leukaemia (ALL) and radon exposure with an ERR per 1000 Bq/m^3 years = 1.44 (95% CI: 0.24 to 3.81). The study had 860 cases of ALL and found no associations with other types of childhood cancer. A 10-fold larger UK study (see paragraph 90 in the annotated bibliography) did not replicate this finding, with an equivalent ERR of 0.03 (95% CI: -4 to 11). **[N_{oco}]**

89. Some parts of the world have high levels of natural background gamma radiation: for example Guarapari in Brazil, Ramsar in Iran, Yangjiang in China and Kerala in India. Average external doses of 5-6 mGy/yr are common in these regions, with higher levels in a few areas. Two large cohort studies have assessed the risks posed by this high background radiation. The Kerala cohort of 70,000 individuals yields an ERR/Gy = -0.13 (95% CI: -0.58 to 0.46) for incidence of cancers excluding leukaemia. The Yangjiang cohort of 81,000 individuals yields an ERR/Gy = 0.19 (95% CI: -0.65 to 3.04) for solid cancer mortality. **[C_{ons}]**

90. Four recent European studies have compared risks of childhood cancer with natural variation in normal background radiation. Statistically significant positive associations were reported in the UK and Switzerland but not in studies in Finland and France. **[N_{oco}]**

91. Residents close to the Techa River, downstream from the Mayak weapons plant which released large quantities of radionuclides into the river in the early years of operations, have had documented raised risks of both solid cancer incidence (ERR/Gy = 0.77 (95% CI: 0.13 to 1.5)) and leukaemia (except CLL) incidence (ERR/Gy = 2.2 (95% CI: 0.8 to 5.4)). For circulatory disease and ischaemic heart disease mortality in the same cohort, whether or not risk was significantly raised depended on the time-lag used in the analysis. **[C_{ons}]**

92. From 1945 to 1980 there were more than 500 atmospheric tests of nuclear weapons. Those tests released radioactive material into the atmosphere, which, as it fell and settled on the ground, created both temporal and spatial patterns of increased exposure to ionizing radiation received both externally and internally. The global average individual effective dose arising from this fallout peaked in the early 1960s at an annual effective dose of around 0.11 mSv. A large-scale study of 11 cancer registries found no evidence of excess cases of childhood leukaemia corresponding to the timing of those atmospheric tests. A study that focussed on Nordic countries (where high rainfall would have led to doses of around 1.3 mSv to the red bone marrow over the four years of highest exposure) did find a slight increase in the incidence of childhood leukaemia in the years just after fallout was at its highest, when compared with children born a few years earlier or later. These two temporal observations are particularly important because the levels of internal emitters from atmospheric tests of weapons are similar to those discharged from nuclear installations. **[C_{ons}]**

93. Residential areas around nuclear facilities.

a. There have been notable clusters of childhood leukaemia close to nuclear installations at Sellafield in England, Dounreay in Scotland and Kruemmel in Germany. **[C_{ons}]**

b. In addition, a case-control study in Germany found an excess of leukaemia in children under 5 years of age living within 5 km from a nuclear power plant.

When matching studies were conducted in the UK and France no such association was found. **[N_{oco}]**

c. Doses from radioactive discharges from the facilities are too low by a factor of 100 to > 1000 to explain the excess cases on the basis of standard risk models (but see paragraph 93g below). Although over a hundred studies in ten countries have failed to find such clusters close to other nuclear facilities, the three clusters and the German case-control study require explanation and various hypotheses have been put forward. **[N_{oco}]**

d. It has long been thought that childhood leukaemia cases tend to occur in clusters – although not every study confirms such clustering. Such clusters can be observed at sites far from nuclear installations. An especially marked cluster has occurred in the rural community of Fallon, Nevada, away from any nuclear installation. **[C_{ons}]**

e. The population mixing hypothesis proposes that childhood leukaemia is a rare complication of a common, but presently unidentified, infection which is augmented when there are marked influxes of urban populations into remote rural areas. **[N_{oco}]**

f. Occupational exposure of fathers prior to conception was also considered as an explanation for excesses of childhood leukaemia, but was not compatible with the data and is now abandoned as a reasonable explanation. **[C_{ons}]**

g. It has recently been re-proposed that radioactive discharges are responsible and that the discrepancy between the calculated risk and the observed numbers of cases can be explained by a combination of temporal spikes in radionuclide emissions, uncertainties in dosimetry calculations for internal emitters and very high radio-sensitivity of embryos and fetuses. However, this explanation is incompatible with the observed incidence of childhood leukaemia after exposure to internal emitters from the fallout from nuclear weapons tests, as well as the numerous studies that have failed to establish the general occurrence of clusters close to nuclear facilities. **[N_{oco}]**

94. Workers in many other industries (particularly those handling oil, gas and phosphates) are exposed to naturally occurring radioactive materials (NORM). Enhanced exposures can occur in these industries but they have not been as closely studied as the workers described above.

95. *Summary.* Radon in the home increases the risk of lung cancer, particularly for smokers. Regions of the world with high natural background radiation do not consistently show an excess risk of solid cancers even in large studies. Fallout from nuclear weapons testing caused low-level internal exposures that were concentrated in time and, to a lesser extent, space, with risks of childhood leukaemia that are consistent with the risks estimated from the LSS. There have been clusters of childhood leukaemia close to and away from nuclear installations that remain unexplained.

MEDICAL EXPOSURE

96. Treatment with radiotherapy for a range of illnesses is effective and common and relies on the ability of radiation to kill cells. After appropriate correction for dose fractionation and cell sterilization effects, radiotherapy data are consistent with risks from the LSS. **[C_{ons}]**

97. Some groups of patients have received doses from internal emitters. Injections of the short-lived alpha-emitter Ra-224 were administered in Germany for the treatment of a number of diseases. A large excess of bone cancers occurred in these patients. Other patients were injected with the radioactive thorium-based diagnostic contrast medium Thorotrast, resulting in a pronounced excess of liver cancer and also of leukaemia. **[C_{ons}]**

98. Medical imaging has undoubted benefits for patients. Currently the effective dose from a single modern digital chest X-ray at 0.014 mSv is very low – equivalent to a few days' natural background exposure – but it would have been greater in the past due to the use of less advanced equipment. However, the newer technology of computed tomography (CT) scanning delivers much higher doses. A single CT scan of the spine can deliver an effective dose of 10 mSv, equivalent to 4 years' background exposure in the UK and the highest diagnostic radiation doses in current practice come from PET/CT scans, where the effective dose from a combined diagnostic whole-body PET and CT investigation is around 20 mSv, equivalent to 8 years' background equivalent. As for radiotherapy, data from diagnostic radiation have to be treated with caution as most individuals are scanned or X-rayed because they have a suspected or previous pathology.

a. As early as 1956, diagnostic X-rays *in utero* were linked to excess paediatric cancer mortality. A single large UK case-control study (the Oxford Survey of Childhood Cancers, OSCC) found a relative risk (RR) of 1.49 (95% CI: 1.33 to 1.67) for leukaemia in childhood and an RR of 1.45 (95% CI: 1.30 to 1.62) for all other childhood cancers after antenatal diagnostic exposure of the maternal abdomen (mainly in the last month of pregnancy). A pooled analysis of 32 other studies reported an RR of 1.32 (95% CI: 1.19 to 1.46) for childhood leukaemia. These results compare children irradiated *in utero* with those who were not, but do not make any further distinction about the level of dose received. The estimated average X-ray dose received by a fetus in 1958 in the UK was just 10 mGy, leading some to question an interpretation of causality underlying the observed risks. Nonetheless, the approximate ERR/Gy estimate for childhood leukaemia that can be obtained from the OSCC is compatible with that from the LSS for those exposed in early childhood **[N_{oco}]**

b. Studies of the risks from diagnostic X-rays for children and adults generally show mixed results. A recent summary of results from larger studies (>30 cases) of diagnostic X-rays and leukaemia found a statistically significant excess in 4 out of 13 studies. A series of studies of patients who received multiple fluoroscopic examinations of the chest whilst being treated for tuberculosis found a radiation-related risk of breast cancer which is very similar to the absolute risk of breast cancer in the LSS: an excess absolute risk (EAR) per 10,000 person years/Sv = 5.48 (95% CI: 0.90 to 10.43) for fluoroscopy patients versus EAR per 10,000 person years/Sv = 4.95 (95% CI: 3.37 to 6.71) in the LSS. This is despite the fact that the fluoroscopy patients' doses were highly fractionated into doses of ~10 mSv given at intervals of 2-3 weeks. A similar finding for breast cancer was reported for female patients who had been exposed to fractionated diagnostic radiation while being monitored for scoliosis. Other studies have reported null dose-response results for lung cancer risk after multiple fluoroscopic examinations. **[E_{mco}]**

c. Several studies have reported dose-related risks of cancer following childhood CT scans. These studies stimulated extensive discussion and suggestions that "reverse causation" might be at play, with early symptoms of cancer or some underlying pre-disposition to cancer causing the need for CT scans, not vice versa. As the use of CT scans globally increases, this is increasingly an important question. **[N_{oco}]**

99. *Summary.* After adjustment for dose fractionation and high-dose cell killing, the risks posed by radiation received as therapy are broadly in line with LSS data. Doses from diagnostic X-rays are much lower, but some studies describe raised risks of childhood leukaemia and other childhood cancers after *in utero* exposure. Recent studies of leukaemia and brain cancer after childhood CT scans report raised risks, but the extent to which the pre-existing health status of the patients might confound this association needs further consideration. The principle of justification emphasises that health benefits of radiation use in medicine must outweigh any radiation exposure risks.

EXPERIMENTAL STUDIES OF MECHANISMS OF DAMAGE

100. A large body of cellular and molecular data supports the idea that ionizing radiation increases the risk of cancer through damage to DNA. This damage is mediated both directly by ionization of the DNA and indirectly via water molecules that become ionized and create products (such as hydroxyl radicals) which may damage DNA and other cellular components. The most important types of DNA damage are double-strand breaks (DSBs), which arise from clustering of ionizations within radiation tracks and are produced linearly with dose. Complex DSBs, which include additional DNA strand breaks or altered DNA bases within very close proximity, are difficult for the cell to repair and very different from the kind of damage that routinely arises during normal metabolism. Such damage can be produced by the lowest possible dose of a single particle track passing through a cell, even by an electron from X-ray exposure but more efficiently by a high-LET particle. **[C_{ons}]**

101. There are several cellular mechanisms that promote DNA repair, but they are not completely efficient and, if mis-repaired, DSBs can produce mutations and chromosome aberrations composed of various types of rearrangements. Counts of chromosome exchange aberrations have an upwardly curving, linear-quadratic dose response, but at doses below 100 mGy the curve is dominated by the linear

component. There is also a clear relationship with dose-rate, in which the same dose delivered at a lower rate yields a smaller number of aberrations. **[C$_{ons}$]**

102. There are other damage-response mechanisms apart from repair of which checkpoint-arrest is the most important in the context of carcinogenesis. Checkpoint arrest pauses the cell-cycle before mitosis or replication, enhancing the chances for accurate repair. One of these mechanisms of checkpoint arrest is only activated when there are more than 20 DSBs in a cell, so does not operate efficiently at doses below about 200 mGy. Failure of checkpoint arrest at low doses may cause hypersensitivity to killing (a phenomenon called low dose hypersensitivity). Whether it confers hypersensitivity to carcinogenesis is unknown. **[E$_{mco}$]**

103. Although chromosome aberrations and rearrangements are often present in malignant cells, it remains unclear precisely what role they play in initiating carcinogenesis. Modern methods of characterising them have revealed a great diversity of types of aberration and it is becoming clear that some types are much more likely to lead to cancer than others. Neither counts of chromosome aberrations nor any other biomarker of radiation dose has yet yielded a validated predictor of cancer risk. **[C$_{ons}$]**

104. It has long been thought that cancers (including radiation-induced cancers) are caused by a multistep process in which a series of particular mutations (so-called 'oncogenic' mutations) accumulate in a cell and its progeny, driving their proliferation, creating cells with clonal advantage and eventually leading to the formation of tumours. Radiation can contribute both to the initial mutations and to the accumulation of later mutations. **[C$_{ons}$]**

105. Such a multistep process takes time, and it is increasingly accepted that only stem cells (or, in some tissues, their daughter progenitor cells) are resident for long enough in the body to accumulate the mutations, and possibly other changes, necessary to become malignant. This has led to an increasing focus on stem cells as the target cells for carcinogenesis. Understanding which cells in the body are the target cells responsible for carcinogenesis is fundamental to understanding its biological basis. **[E$_{mco}$]**

106. The cellular response to a dose of radiation can depend upon that cell's prior exposures. In adaptive responses a priming dose reduces the damage done by subsequent doses, with the frequency of chromosomal aberrations after the second (challenge) dose reduced. The priming dose is usually in the range of 1 – 100 mGy and the challenge dose is larger. The mechanism of action is via the induction of additional repair processes. **[C$_{ons}$]**

107. Radiation can have an impact upon cells which are not themselves irradiated. Such "non-targeted effects" can be seen in cells in close proximity to irradiated cells, and at distant sites and in the progeny of irradiated cells. Non-targeted effects fall into two broad categories: genomic instability, and bystander effects.
    a. Genomic instability describes the observation that the progeny of irradiated cells have an enhanced rate of generating genetic change. Genomic instability can be induced by doses as low as 10 mGy.
    b. In bystander effects irradiated cells transmit signals of their damage to non-irradiated cells. Various responses are observed in cells that receive these signals – including cell death, adaptive responses and chromosomal damage. These could be either beneficial (e.g. increasing the likelihood that damaged cells will die so reducing eventual cancer risk) or detrimental (e.g. increasing the number of damaged cells). Bystander effects appear to be a low-dose effect and are not seen in all experimental systems. The existence of bystander effects demonstrates that radiation damage occurs at the level of populations of cells organised in tissues and organs, and not just a process that concerns individual, isolated cells.

    These processes (adaptive response and non-targeted effects) could act to either increase or decrease risks at very low dose and it is not yet clear how important they are in relation to radiogenic disease *in vivo*. Whether they are beneficial or detrimental in terms of health effects, when they occur, their impact will be included in the risks observed in epidemiological studies. Their importance is therefore in consideration of the expected shape of the dose-response curve at low doses and low dose-rates. **[N$_{oco}$]**

108. Recent advances in biotechnology allow measurement of the expression of genes and proteins in cells. These techniques are called “transcriptomics” and “proteomics” when they study the expression of genes and proteins respectively. There is now substantial evidence showing that such cellular responses after low dose exposure show differences from those after higher doses, with activation of stress responses being the most significant changes. **[C_{ons}]**

109. If there were a mechanistic understanding of the pathways linking an initial radiation event to a consequent tumour, along with proper quantification of each step, it would be possible to derive the dose-response relationship. That understanding would need to comprise the initial damage and all the defence mechanisms – at the cell, tissue and organismal level. For cancer, the dose response for initial damage is expected to be linear and most subsequent processes to be non-linear. This might eventually provide support for one or other of the theoretical shapes of the dose response relations for cancer or non-cancer diseases in the low-dose and low dose-rate region where reliable epidemiological results are difficult to obtain. **[E_{mco}]**

110. There is no *a priori* reason to assume that the dose-response relationship will be the same for all types of cancers. There are likely to be different dose response relations for non-cancer diseases such as cardiovascular disease, where the aetiology of the diseases may be different from the cancer aetiology. **[E_{mco}]**

111. In vitro studies suggest that chronic low dose-rate exposures can induce premature senescence in endothelial cells. Premature senescence in different organs could contribute to low-level radiation risk with implications for the immune defence and neurological disease. **[E_{mco}]**

112. Mechanistic studies *in vitro* and in animal models at low dose and low dose-rate which aim to understand the cellular processes that are induced from functional aspects may eventually also provide new biomarkers for exposure, disease and tissue sensitivity.

113. It is widely believed that individuals differ in their inherent susceptibility to radiation induced carcinogenesis. It would be very useful to be able to identify individuals with higher than average susceptibility (e.g. risk-benefit calculations concerning therapeutic and diagnostic radiation are different for people with higher than average sensitivity to radiation.) Apart from a few rare genetic disorders (see paragraph 19b), variation in radio-sensitivity is not yet properly understood. A better understanding of the mechanisms of disease induction should, eventually, allow a deeper understanding of such variation in individual sensitivity to radiation.

114. The biological mechanisms that underlie cardiovascular disease caused by medium and high doses of radiation are microvascular changes and atherosclerosis (the thickening of artery walls because of the accumulation of white blood cells) through pro-inflammatory effects. It is likely that the mechanisms at low doses or low dose-rates will have differences from those at medium and high doses and long-term changes in immunity are postulated to be involved. **[E_{mco}]**

115. The mechanism whereby ionizing radiation induces cataracts is not well understood. Cataracts are defined as progressive opaqueness of the lens of the eye, leading to loss of vision. Genomic damage of lens epithelial cells is considered one of the key mechanisms and such damage has been observed in mice experimentally exposed to whole body doses as low as 20 mGy. Oxidative damage, changes in morphology and altered cell signalling also play a role. **[E_{mco}]**

116. *Summary* Studies *in vitro* have clearly established that radiation can damage DNA in ways that if mis-repaired could, *in vivo,* lead to cancer. Because of the stochastic nature of interactions of radiation with DNA and other molecules, it is reasonable to expect initial damage at low doses to have a linear dose-response, but subsequent cellular responses may not have a linear dose response and may be different at low versus high doses. Despite much elucidation of the underlying cellular processes it is still not clear precisely what steps are necessary and sufficient for a dose of radiation to eventually lead to cancer (sometimes decades later). Currently there are no validated bio-markers of radiation-induced cancers. Understanding of the mechanisms whereby radiation causes cardiovascular disease and cataracts is still less advanced.

EXPERIMENTAL STUDIES THAT INFORM RISK ASSESSMENT

117. *In vitro* studies of radiation-induced DNA damage and subsequent mutations and chromosome aberrations have yielded substantial information on the shape of the dose-response relationship, and on the modulating effects of dose rate. Chromosome aberration counts from a number of studies follow a linear dose-response at doses in the range of 20 – 100 mGy, but below 20 mGy the data cannot distinguish between a linear model and a model with a threshold. Values for DDREF in such studies are generally in the range 2 – 4 implying that, compared to the risks from acute high doses, risks from low doses or doses delivered at low doses rate are halved or quartered. **[C_{ons}]**

118. Animal experiments with cancer endpoints illustrate the diversity of dose response curves seen for different types of cancer. Some experimental systems generate data compatible with a linear-quadratic model: for example the induction of leukaemia in mice at doses of 250 mGy to 3 Gy or mammary cancer in female mice at doses of 100 mGy to 2 Gy. But other kinds of cancer show a linear dose response: for example mammary tumours in Sprague-Dawley rats at doses of 100 mGy to 2 Gy. A threshold effect is observed for skin cancer in both mice and rats with no tumours generated at doses below about 10 Gy. Most of these studies do not explore doses in the range below 100 mGy, with the smallest dose usually at 100 mGy. Values for DDREF from appropriate animal carcinogenesis studies are in the range 4-5. **[C_{ons}]**

119. Mice and dogs have been used in a large number of experiments that study the life-shortening effects of radiation. When doses and dose-rates are low most early deaths in these experiments are from radiation-induced cancers. There is a common pattern across many of these studies in which the reciprocal of mean age at death rises linearly with total dose. When the total dose is delivered at a lower dose-rate the slope of the linear relationship is smaller. **[C_{ons}]**

120. Studies of radiation-induced heritable effects in mice form the main basis for quantitative estimates of the risk of heritable disease in humans by UNSCEAR and ICRP. Such studies count the number of radiation-induced mutations in the offspring of irradiated male mice. The conclusion from studying tens of thousands of such offspring is that at ~ 1 Gy of chronic low-LET radiation the number of radiation-induced mutations in one generation is as large as the number that arise from other causes. **[C_{ons}]**

121. Probably the largest contribution to radiation protection from experimental data has been for decisions on radiation weighting factors. ICRP (and others) have relied heavily on both *in vitro* and animal experiments because of the paucity of appropriate human data. **[C_{ons}]**

122. The BEIR VII report combined radiobiological evidence from animal experiments with the LSS data in a Bayesian statistical analysis in order to estimate a DDREF (defined at paragraph 24b) from both kinds of data. The radiobiological evidence came from mouse experiments comparing acute, fractionated and chronic doses on both cancer risk and on life-shortening. The resulting estimate was DDREF = 1.5 (95% CI: 1.1 to 2.3). Despite the sophistication of the approach, the committee drew attention to how difficult it is to measure DDREF with the comment that it "recognizes the limitation of the data and the uncertainties in estimating the DDREF". The DDREF is under further investigation. **[E_{mco}]**

123. *Summary.* Studies *in vitro* demonstrate a linear dose-response for chromosome aberrations at doses between 20 mGy and 100 mGy. Irradiation of animals has clearly established that moderate and high doses of radiation (usually 100 mGy to several Gy) can cause cancer and life-shortening (also largely due to cancer). Dose response relationships at low dose are mostly linear. Irradiation of male mice before mating has demonstrated that radiation-induced mutations can be passed to offspring in a manner that is proportional to parental dose. Analysis comparing dose-response slopes at low and high doses implies that radiation delivered at a low dose or a low dose rate carries 2 – 4 fold less risk than acute doses of the same total dose. An equivalent analysis that combines human epidemiological data and animal experimental data estimated that the DDREF may be only about 1.5-fold, and this factor is under further investigation.

PERSPECTIVES

124. The risks from ionizing radiation have been, and continue to be, exceptionally well studied and can be compared with the risks posed by other factors.

a. Whilst the risks at very low levels of a few millisievert can be inferred only by extrapolation, risks at higher levels can be directly measured. Comparing those risks with the impact of other

insults to health is illuminating. An individual in the very heavily exposed group of survivors of the Japanese atomic bombings (those who received > 1 Gy) can on average expect to lose fewer years of life than a lifelong smoker or someone who is severely obese (Table 5). **[C_{ons}]**

b. Across the world, radiation arising from residential radon poses health risks to very large populations. Nevertheless, a comprehensive analysis of the global modelled burden of disease attributable to a wide range of risk factors compared estimated deaths from residential radon and found them to be around 30-60 times lower in number than deaths that could be attributed either to ambient particulate matter pollution, or to tobacco smoking (Table 5). **[C_{ons}]**

125. Table 5. Comparisons of average years of life lost and numbers of attributable deaths for radiation versus other risks.

**Average years of life lost**		
Japanese atomic bomb survivor in the very heavily exposed group (>1 Gy)	35 year old white, severely obese male	Lifetime smoking male doctor
2.6 years	4 - 10 years	10 years
**Annual number of attributable deaths, worldwide, 2010**		
Residential Radon	Air pollution, ambient particulate $PM_{2.5}$	Tobacco Smoking
99,000	3,200,000	6,300,000

126. *Summary.* Compared with other common health risks (obesity, tobacco smoking, exposure to ambient particulate air pollution), the number of years of life lost due to radiation exposure is small.
