# Supplementary material for: A restatement of the natural science evidence base concerning the health effects of low-level ionizing radiation
Source: Proc Biol Sci. 2017 Sep 13;284(1862):20171070. doi: 10.1098/rspb.2017.1070 (PMC5597830; doi:10.1098/rspb.2017.1070)
Supplement: APPENDIX B [file rspb20171070supp2.pdf]

Appendix B
A restatement of the natural science evidence base concerning the health effects of low-level ionizing radiation

Proceedings of the Royal Society B DOI 10.1098/rspb.2017-1070

Angela R. McLean, Ella K. Adlen, Elisabeth Cardis, Alex Elliott, Dudley T. Goodhead, Mats Harms-Ringdahl, Jolyon H. Hendry, Peter Hoskin, Penny A. Jeggo, David J.C. Mackay, Colin R. Muirhead, John Shepherd, Roy E. Shore, Geraldine A. Thomas, Richard Wakeford & H. Charles J. Godfray

Paragraph numbering corresponds to that in the main document. Website URLs were accessed 29/11/2016.

INTRODUCTION AND AIMS

1. The UK National Physical Laboratory has an educational poster on the subject of ionizing radiation http://www.npl.co.uk/educate-explore/factsheets/ionising-radiation/. Three major publications summarise the natural science evidence base: that from the United Nations UNSCEAR (2006a), that from the US National Academies BEIR VII (2006) and the latest recommendations of the International Commission on Radiological Protection ICRP 103 (2007).
2. See UNSCEAR (2006a) (Annex A, Section I), and the epidemiology guidelines published by Bradford-Hill in 1965 and reproduced in 2015 Hill (2015) with the accompanying editorial Wakeford (2015). Doll (2002) gives a further review.
3. UNSCEAR (2008) gives global figures on natural and artificial background radiation. Abbot (2015) gives comparative figures for different countries and different years. In this paper we define, for sparsely ionizing radiation, a low dose as being <100 mGy and a low dose-rate as being <0.1 mGy/min averaged over one hour, following UNSCEAR (2012b).
4. The use of the descriptors [Cons], [Emco], [Noco] and [Projn] follows practice in previous restatements Godfray *et al.* (2013), Godfray *et al.* (2014), Godfray *et al.* (2015), Dadson *et al.* (2017).
5. Since its inception UNSCEAR has published 25 major reports available here: http://www.unscear.org/unscear/en/publications.html. The National Academies series of BEIR reports are available here: http://www.nap.edu/search/?term=BEIR . ICRP publications are here: http://www.icrp.org/publications.asp.

THE SYSTEM OF RADIOLOGICAL PROTECTION

6. Chapter 5 of ICRP 103 (2007) describes the system of radiological protection of humans. Wrixon (2008) provides a summary and contextualises the changes in recommendations.
7. ICRP website at: http://www.icrp.org/index.asp
8. See Cooper (2012) for a summary of current radiation protection principles. Chapter 8 of ICRP 103 describes protection of the environment.
   a. ICRP 103 (2007), IAEA (2014). Countries within the European Union are currently working to enact the Basic Safety Standards in BSS 2013/59/Euratom Euratom (2013), work which they are obliged to complete by February 2018. The most recent and only change to the 2007 ICRP recommendations is the Statement on Tissue Reactions, specifically the reduced dose limits to the eye lens Stewart *et al.* (2012).
   b. UNSCEAR (2008), BEIR VII (2006). Figure 3.1 in Clarke and Valentin (2009) represents the basis for and use of ICRP recommendations in a flow chart. Some of the proponents of alternative theories about the dangers (or otherwise) of radiation do not view reports by these international bodies as authoritative and instead view them as representing an establishment position that attempts to argue 'from authority'. However, these reports are widely recognised as representing the views of the majority working in the field.
9.
   a. UNSCEAR (2008). Table 8, pages 116-117 of ICRP 103 (2007) summarises the current recommendations for radiological protection in planned, emergency and existing situations.
   b. See Oatway *et al.* (2016) for UK radiation exposures.
   c. ICRP 103 (2007) Paragraph 2.2, page 42 and Paragraph 5.6, page 88.
   d. ICRP 103 (2007) Paragraph 5.7, page 89.
   e. ICRP 103 (2007) Paragraph 5.8, page 91, Mobbs *et al.* (2011). For an example of an optimization procedure in the context of management of occupational exposure, see Figure 1 in IAEA (2002).
   f. For example, the average annual dose to occupationally exposed workers in the UK is 0.0004 mSv Oatway *et al.* (2016). For sources that arise from the disposal of radioactive waste the recommended effective dose constraint for public exposure is 0.3 mSv/yr and for prolonged exposure from long-lived radionuclides, if dose assessment is problematic, the recommended dose constraint is <0.1 mSv/yr. A radionuclide is a particular version of an atomic nucleus characterised by its number of protons and neutrons and their arrangement within the nucleus. For example iodine-131 (^{131}I) is the radionuclide of iodine with 53 protons and 78 neutrons, whereas the nucleus of technetium-99m has 43 protons and 56 neutrons in a metastable state.
   g. Lecomte *et al.* (2014) refers to an upper reference level of 300 Bq/m^3 for radon-222 corresponding to about 4.5 mSv/y in workplaces and 15.8 mSv/y in homes. Radon-related risk in the home is determined by a person's smoking status as well as indoor radon concentration Gray *et al.* (2009). The phrase "according to the situation" is a quote from Table 8 ICRP 103 which refers to the judgement required when levels of radiation are abnormally high.
   h. Chapter 7 of ICRP 103 (2007) describes the system of recommendations for medical exposures. See also Wrixon (2008).
10. Brenner (2010), Muller (2015), Auvinen *et al.* (2015).
11. Morgan and Bair (2013), Niles (2014), Pearce (2015). Annex IV of UNSCEAR (2008) and the WHO (2006) Chernobyl Forum report both document the large health impact of the Chernobyl accident because of fears about radiation.
12. Authors' summary.

DEFINITIONS AND UNITS

Table 6. List of abbreviations.

**Organizations**	
BEIR	Biological Effects of Ionizing Radiation
CERRIE	Committee Examining Radiation Risks of Internal Emitters
COMARE	Committee on Medical Aspects of Radiation in the Environment
IAEA	International Atomic Energy Agency
ICRP	International Commission on Radiological Protection
ICRU	International Commission on Radiation Units and Measurements
RERF	Radiation Effects Research Foundation
UN	United Nations
UNSCEAR	United Nations Scientific Committee on the Effects of Atomic Radiation
WHO	World Health Organization
**Units**	
Bq	becquerel
Gy	gray
PM	Particulate Matter
Sv	sievert
WLM	Working Level Month
**Cohorts/Studies**	
ECLIS	European Childhood Leukaemia-Lymphoma Study
KiKK	Kinderkrebs in der Umgebung von Kernkraftwerken
LSS	Life Span Study
OSCC	Oxford Survey of Childhood Cancers
**Other Acronyms**	
ALL	Acute lymphocytic leukaemia
CI	Confidence interval
CLL	Chronic lymphocytic leukaemia
CT	Computed tomography
DDREF	Dose and dose-rate effectiveness factor
DNA	Deoxyribonucleic acid
DSB	Double strand break
EAR	Excess absolute risk
ERR	Excess relative risk
HR	Hazard ratio
LD	Lethal dose
LET	Linear energy transfer
LNT	Linear no threshold
NIC	Not in city
NPP	Nuclear power plant
OR	Odds ratio
RBE	Relative biological effectiveness
RR	Relative risk
SI	System Internationale d'Unites or international system of units
SIR	Standardized incidence ratio
SMR	Standardized mortality ratio
SRR	Standardized rate ratio (or standardized registration ratio)
UV	Ultra violet

13. Chapter 4 in ICRP 103 (2007) is devoted to explanations and definitions of doses, exposures and the associated uncertainties. The International Commission on Radiation Units and Measurements (ICRU) issues periodic reports, in particular see ICRU (2011).
14. Chapter 1 in Mettler and Upton (2008) covers basic radiation physics, chemistry and biology and Chapter 2 covers sources of exposure. CERRIE (2004) reviewed data on radiation risks of internal emitters as does COMARE (2004). Tables 2 and 3 in ICRP 103 (2007) document radiation weighting factors and tissue weighting factors. There are no universally agreed definitions of low dose or low dose-rate. Tables 6 and 7 in UNSCEAR (2006a) summarize definitions that have been used for low dose and low dose-rate respectively. Ruhm *et al.* (2016) summarise recent evidence and debate on the impact of dose-rate upon the health effects of radiation.
15. BIPM (Bureau International des Poids et Mesures) pages defining SI units for ionizing radiation are at: http://www.bipm.org/en/measurement-units/history-si/radioactivity/. Tables 2 and 3 in ICRP 103 (2007) document radiation weighting factors and tissue weighting factors.
16. Streffer *et al.* (2003). The recommended ICRP limits to prevent deterministic effects are set in equivalent dose, although strictly these should be set in absorbed dose.
17. The relationship between activity (Bq) and the resulting dose (Sv) depends on multiple factors including the nature and energy of the decay, and the location of the radioactive material with regards to the receptor. See Marsh *et al.* (2010), Tirmarche *et al.* (2010), Harrison and Marsh (2012), Muller *et al.* (2016).
18.
    a. Tissue reactions are discussed more fully under the section "Acute high dose exposures", paragraphs 26-31.
    b. Stochastic effects are discussed more fully under the section "Lower dose exposures", paragraphs 32-40.
    c. Brenner *et al.* (2003), Doss (2013).
    d. The matched pair of review articles by Little *et al.* (2009) and Tubiana *et al.* (2009) presents the arguments for and against LNT. Also see paragraph 24.
    e. Stewart *et al.* (2012) is the ICRP document reviewing both circulatory disease and diseases of the eye after radiation exposure. Little *et al.* (2008b), Little *et al.* (2012), Little (2013) review the literature on the risks of non-cancer disease and radiation. Kitahara *et al.* (2015) summarise data on low dose radiation and circulatory disease or cataracts published after 2006. Darby *et al.* (2013) found a linear dose-response relationship between radiation-related risks and major coronary events, suggesting that the LNT concept may be relevant to some forms of circulatory disease, although this was a study of high doses received during radiotherapy.

Table 7. Major types of ionizing radiation and their properties.

Radiation type	Wave or particle type	Radiation weighting factor, w_R	Effective Shielding	High/Low Linear Energy Transfer	Common source
X rays	Electromagnetic wave	1	Lead plate	Low	Diagnostic x rays
Gamma rays	Electromagnetic wave	1	Lead plate	Low	Terrestrial radionuclides and building materials
Beta particles	Emitted electron or positron	1	Aluminium plate	Low	Strontium-90 in radiotherapy
Neutrons	Emitted neutron	5-20	Metres of water or concrete	High	Neutron component of cosmic radiation
Alpha particles	2 protons + 2 neutrons	20	Sheet of paper	High	Radon

19. See UNSCEAR (2006a) pages 32-33 on human genetic susceptibility. Susceptibility and the development of biomarkers to identify radio-sensitive cancer patients is discussed in Manning and Rothkamm (2013).
    a. Whilst the excess relative risk of cancer is higher for exposures in childhood than for those in adulthood, the difference in excess absolute risk is not as marked (see paragraph 37b). Ozasa *et al.* (2012) document the impact of age at exposure on solid cancer mortality risk in Japanese atomic bomb survivors; Hsu *et al.* (2013) do the same for leukaemia.
    b. BEIR VII (2006) reviews data on genetic susceptibility to radiation-induced cancer noting unambiguous evidence of radio-sensitivity for two human genetic disorders: ataxia-telangiectasia Easton (1994) and Nijmegen breakage syndrome Zhao *et al.* (2000).
    c. See Sigurdson and Stram (2012) on potential implications for risk assessment.
    d. Darby *et al.* (2005), Pierce *et al.* (2003), Furukawa *et al.* (2010).
20. Authors' summary.

BACKGROUND EXPOSURE AND UNCERTAINTIES AT LOW DOSE

21. Oatway *et al.* (2016) review radiation exposure of the UK population based on data for 2010. Their Table 1 breaks exposure down for UK doses by source. UNSCEAR (2008) gives global averages. See NCRP (2009) for US exposures. Reference levels for exposures in emergencies would be much greater than background levels. The limits for planned situations are predicated on what might be acceptable or tolerable levels of risk for on-going continuous exposures from operations involving the use of radiation – for instance in the nuclear power industry or in hospitals.
22. For radon measurements at fine scales see Chen and Ford (2016). Hughes *et al.* (2005) report that some homes in the UK give rise to individual annual effective doses exceeding 100 mSv from radon, and a house with a very high radon level giving an annual effective dose to the occupants of 1.2 Sv has been reported from Ireland Organo *et al.* (2004). Construction differences such as sealed double glazing and closed cycle heating have an impact on radon levels via pressure differences UNSCEAR (2006b). Bossew *et al.* (2015) report on the European atlas of natural radiation. The map of indoor radon concentration, which covers 25 countries, is the most advanced component of that project Tollefsen *et al.* (2014), Hoffmann *et al.* (2016). Maps of geogenic radon potential, secondary cosmic radiation, terrestrial gamma radiation and concentration of the elements U, Th and K are also under construction.
23. Figure 2 refers to data from large studies of excess relative risk at different radiation doses. In this figure, relative risk (RR), odds ratios (OR) and standardised rate ratios (SRR) have been approximated to excess relative risk (ERR), under the assumption that the populations are homogenous and that the probabilities of the underlying diseases are small. Figure 2a describes excess relative risks of solid cancers. The **Japanese Life Span Study (LSS)** cohort are Japanese atomic bomb survivors Ozasa *et al.* (2012). The **international workers** cohort is a very large pooled study of radiation workers (INWORKS), most of whom are employed in the nuclear industry, from the UK, US, and France Richardson *et al.* (2015). Kashcheev *et al.* (2015) describe risks in **Chernobyl workers** who cleaned up after the accident. **Mayak workers** at the eponymous nuclear plant were subject to prolonged low dose-rate external gamma radiation and plutonium exposure Sokolnikov *et al.* (2015), as were nearby **Techa River residents** due to discharges of radioactive waste Davis *et al.* (2015). **Yangjiang** is an area of high natural background radiation in China Tao *et al.* (2012) and **Kerala** is an area of high natural background radiation in India Nair *et al.* (2009). **Ankylosing spondylitis** patients in the UK were historically treated with X-rays in the mid-20th century Weiss *et al.* (1994).
    Figure 2b describes excess relative risks of leukaemia for the **Japanese Life Span Study (LSS)** Hsu *et al.* (2013), **international workers** in the INWORKS nuclear worker cohort Leuraud *et al.* (2015), **Chernobyl clean-up workers** Zablotska *et al.* (2013) as well as a different cohort of **Chernobyl liquidators** Kesminiene *et al.* (2008), **Mayak workers** Kuznetsova *et al.* (2016), **Techa River residents** Krestinina *et al.* (2013b), residents receiving exposure from **Kerala** background radiation in India Nair *et al.* (2009) and patients with **ankylosing spondylitis** in the UK Weiss *et al.* (1995).
    For the purposes of representation and as the studies pertain almost entirely to low-LET radiation, Sv and Gy are assumed to be equivalent in Figures 2a and 2b. For the purposes of representation, ERRs for cancer incidence and mortality are also plotted on the same axis. In reality, the relationship between cancer incidence and mortality will depend on the ability and availability of diagnostics and therapies to improve survivorship. See Coleman *et al.* (1993).
    Figure 2c describes excess relative risks of lung cancer for underground miners Lubin *et al.* (1995), Villeneuve *et al.* (2007), Schubauer-Berigan *et al.* (2009), Lane *et al.* (2010), Tomasek (2012), Kreuzer *et al.* (2015a) or after exposure to residential radon in Europe Darby *et al.* (2005), China Lubin *et al.* (2004) and North America Krewski *et al.* (2006).
    For underground miners, risks are expressed relative to cumulative radon exposures given in WLM, and for domestic exposures to radon, risks are expressed relative to concentrations in Bq/m^3.
    These diverse studies include individuals who have been exposed at greatly varying dose-rates – some briefly, others very slowly. Ruhm *et al.* (2016) summarise recent evidence and debate on the impact of dose-rate upon the health effects of radiation, although a distinction needs to be made between low-LET and high-LET radiation.
    Table 8 contains further detail about the data in this figure.

Table 8. Components of Figure 2.

	Cohort	Reference	Reference	Endpoint	Confidence Intervals	Y-Axis	X-Axis
**Figure 2a**	Japanese life span study (LSS)	Ozasa *et al.* (2012)	Figure 4	solid cancer mortality	95%	ERR	weighted colon dose, Gy
	International workers (INWORKS)	Richardson *et al.* (2015)	Figure 1	all cancer mortality other than leukaemia	90%	RR*	cumulative colon dose, Gy
	Chernobyl workers	Kashcheev *et al.* (2015)	Figure 6	solid cancer mortality	95%	RR*	dose, Gy
	Mayak workers	Sokolnikov *et al.* (2015)	Figure 2	solid cancer mortality ex bone, lung and liver	95%	ERR	colon dose, Gy
	Techa River residents	Davis *et al.* (2015)	Figure 1	solid cancer incidence	none	ERR	cumulative dose, Gy
	Kerala background radiation	Nair *et al.* (2009)	Table 4	all cancer incidence other than leukaemia	95%	RR*	cumulative radiation dose, Gy
	Yangjiang background radiation	Tao *et al.* (2012)	Table 2	all cancer mortality excluding leukaemia	95%	RR*	cumulative individual dose (range midpoint), mGy
	Ankylosing spondylitis patients	Weiss *et al.* (1994)	Figure 2	all neoplasms mortality except leukaemia	95%	RR*	total body dose, Gy
**Figure 2b**	Japanese life span study (LSS)	Hsu *et al.* (2013)	Figure 1b	leukaemia incidence ex CLL & ALL	none	ERR	weighted red bone marrow dose, Gy
	International workers (INWORKS)	Leuraud *et al.* (2015)	Table A2	leukaemia mortality excluding CLL	90%	RR*	dose, Gy
	Chernobyl workers (Ukraine)	Zablotska *et al.* (2013)	Figure 1	leukaemia incidence excluding CLL	95%	RR*	bone marrow dose, Gy
	Chernobyl liquidators (Belarus, Russia & Baltic countries)	Kesminiene *et al.* (2008)	Figure 1	leukaemia incidence excluding CLL	95%	OR	total red bone marrow dose, Gy
	Mayak workers	Kuznetsova *et al.* (2016)	Table 3	leukaemia incidence excluding CLL	None	RR	external dose to bone marrow, Gy (range midpoint)
	Techa River residents	Krestinina *et al.* (2013b)	Figure 1	leukaemia incidence excluding CLL	none	ERR	red bone marrow dose, Gy
	Kerala background radiation	Nair *et al.* (2009)	Table 6	leukaemia incidence	95%	RR*	cumulative radiation dose, Gy
	Ankylosing spondylitis patients	Weiss *et al.* (1995)	Table IV Figure 1	leukaemia mortality excluding CLL	95%	RR*	total marrow dose, Gy
**Figure 2c**	Canadian uranium workers	Lane *et al.* (2010)	Figure 1	lung cancer mortality	95%	RR*	WLM
	Uranium & tin miners	Lubin *et al.* (1995)	Figure 1a	lung cancer mortality	95%	RR*	cumulative WLM
	German uranium miners	Kreuzer *et al.* (2015a)	Figure 1a	lung cancer mortality	95%	RR*	cumulative WLM
	Colorado uranium miners	Schubauer-Berigan *et al.* (2009)	Table 4	lung cancer mortality	95%	SRR*	cumulative WLM (range midpoint)
	Czech uranium miners	Tomasek (2012)	Figure 1	lung cancer mortality	90%	O/E*	cumulative WLM
	Newfoundland fluorspar miners	Villeneuve *et al.* (2007)	Table 4	lung cancer mortality	95%	RR*	cumulative WLM
**Figure 2c**	China residential	Lubin *et al.* (2004)	Table 3	lung cancer incidence	95%	OR*	Radon concentration (range midpoint) Bq/m3
	North America residential	Krewski *et al.* (2006)	Figure 1A	lung cancer incidence	95%	OR*	Radon concentration Bq/m3
	Europe residential	Darby *et al.* (2006)	Figure 7	lung cancer incidence	95%	RR*	radon concentration Bq/m3 (corrected for random variation)

*converted to ERR by the approximations RR=SRR=OR=ERR+1

24. Figure 3 is adapted from Brenner *et al.* (2003) who review the biophysical processes that could generate the different curves. Valentin (2005) is a comprehensive review of radiation-related cancer risk extrapolation at low dose. The matched pair of review articles by Little *et al.* (2009) and Tubiana *et al.* (2009) present the arguments for and against LNT. There are many different experimental systems and different endpoints for modelling radiation carcinogenesis. Within one system, experiments are often replicable but they do not give a common answer to describe the shape of the dose response curve at very low dose.
    a. Little *et al.* (2009) argue that LNT is "…(almost) the best we can do". Shah *et al.* (2012) argue that LNT is "prudent" as the basis for radiation protection policy. Calabrese and O'Connor (2014) give a critical history of the development and adoption of the LNT model.
    b. The BEIR VII (2006) report used a Bayesian analysis that combined epidemiological and experimental data and settled on an estimate of DDREF = 1.5. Page 53 of ICRP 103 (2007) uses DDREF = 2. DDREF combines considerations of total dose and rate of delivery. Sometimes those are given separate consideration as: LDEF (low-dose effectiveness factor); and DREF (dose-rate effectiveness factor) Ruhm *et al.* (2016). Ruhm *et al.* (2015) summarises the development of the DDREF concept and the current findings of the active ICRP Task Group 91 in assessing its applicability. See our paragraph 122.
    c. Figure 5 in Brenner *et al.* (2003) illustrates how a very small, highly radiosensitive population could generate a downwardly curving dose-effect relationship. To date, most studies do not support a dose response curve of this shape. Other hypothetical mechanisms could also lead to such an expectation, for example bystander effects or low-level radiation-induced instability discussed at paragraph 107.
    d. Cornforth *et al.* (2002a) and Sachs *et al.* (1997) model the formation of chromosome aberrations. In their models it is interaction between points of damage from separate radiation tracks that brings in the upward curvature of the dose response. In Cucinotta *et al.* (2000) it is competition between repair pathways that brings in the curvature. Other biophysical mechanism/models are reviewed in Bodgi *et al.* (2016).
    e. Tubiana (2005) summarises the conclusions of a joint report by the French Academie des Sciences and the Academie Nationale de Medecine which argues for the existence of threshold doses below which risk is negligible or zero. See Fritz (2002) for a study on dogs examining chronic whole-of-life effects which has similar conclusions.
    f. Vaiserman (2010) gives a historical review of arguments in favour of radiation hormesis. Feinendegen (2005) presents a mechanistic argument for radiation hormesis, in particular his Figure 3 presents the argument that whilst the induction of DNA damage is expected to be linear in dose, protective responses against such damage are so strongly stimulated at low dose that there is a net hormetic effect. Luckey (2011) hypothesises that the optimal dose is 100 mGy/yr with a threshold at 10 Gy/yr separating beneficial from harmful effects. Sacks *et al.* (2016) argue that epidemiological studies based on linear assumptions are invalid because of hormesis, although it is usual in such studies to test for the appropriateness of a linear fit to the data.
25. Authors' summary.

ACUTE HIGH DOSE EXPOSURES

26. The Office of Science of the US Department of Energy has produced a chart summarizing the health effects of radiation at doses at different orders of magnitude. https://www.nrc.gov/docs/ML1209/ML120970113.pdf
27. ICRP 103 (2007) (Table A.3.3 page 167) lists cause of mortality at different dose thresholds. See also Edwards and Lloyd (1998) Table 1 which gives threshold doses for different tissue syndromes leading to mortality. Mettler and Upton (2008) Chapter 6 reviews tissue reactions across a range of tissues and doses. See Donnelly *et al.* (2010) on medical aspects of acute radiation syndrome.
28. ICRP 103 (2007) (Tables A.3.4. and A.3.3 pages 167-8) and Stewart *et al.* (2012) (Table 2.4, page 298) list thresholds for 1% incidence of morbidity and mortality involving various tissues. Edwards and Lloyd (1998) and Mettler and Upton (2008) as above. Several of the morbidity early effects in Table 1 are primarily of concern in partial body irradiation, for instance erythema (skin) and permanent sterility (gonads). Radiation effects upon cataracts and circulatory disease do not fall neatly into either tissue reaction or stochastic effects.
29. Streffer *et al.* (2003). Chapter 8 in Mettler and Upton (2008) gives a detailed review of tissue reactions of *in utero* exposure to radiation. Otake and Schull (1998) review radiation-related brain damage and growth retardation amongst prenatally exposed survivors.
30. Otake *et al.* (1990) describe studies of untoward pregnancy outcome (defined as stillbirth, major malformation or death within 14 days) amongst >65,000 offspring of atomic bomb survivors finding a positive association with joint parental dose, but the regression slope was not statistically significant. Fujiwara *et al.* (2008) found no evidence of an increased prevalence of adult-onset multi-factorial disease amongst 11,951 adult offspring (median age 50 years) of atomic bomb survivors. Odds ratios at a paternal or maternal dose of 1 Gy were 0.91 (0.81 – 1.01) and 0.98 (0.86 – 1.10) respectively. Neel and Schull (1991) is a book-length collection of essays on the children of atomic bomb survivors. Nakamura (2006) reviews more recent studies of the same children. Neel *et al.* (1990) summarise 40 years' effort to quantify the genetic effects of the atomic bombs with the words "no statistically significant findings have emerged". The most recent report of mortality amongst 75,000 offspring, after 62 years of follow-up, showed no excess of either cancer or noncancer mortality in relation to either paternal or maternal radiation dose Grant *et al.* (2015). See Searle (1974) and Nakamura *et al.* (2013) for animal studies.
31. Authors' summary.

LOWER DOSE EXPOSURES

32. Preston *et al.* (2003), Ozasa *et al.* (2012) Grant *et al.* (2017).
33. Preston *et al.* (2003), Shimizu *et al.* (2010), Little *et al.* (2012), Ozasa *et al.* (2012), Ozasa *et al.* (2016), Baselet *et al.* (2016), Little (2016), Ozasa *et al.* (2017), Shore (2016a).
34. Brenner *et al.* (2003), National Research Council (1995). New approaches in genomics and epigenetics offer promising advances in the ability to distinguish radiation induced cancers Behjati *et al.* (2016).
35. Shore (2009).
36. Land (1980). UNSCEAR 2006 Annex A Paragraph 16 discusses the problems of inadequately powered studies generating results which can overstate true risk despite being statistically significant. Pooled analyses and meta-analyses can sometimes

combine data from several smaller studies to create one well-powered study. Studies of radon in the home Lubin *et al.* (2004), Darby *et al.* (2006), Krewski *et al.* (2006) and of nuclear fuel cycle workers Leuraud *et al.* (2015), Richardson *et al.* (2015) have exploited this strategy.

37. The glossary in ICRP 103 (2007) defines ERR and EAR. Sistrom and Garvan (2004) explains relative risk (RR) and odds ratios (OR). Hernán (2010) discusses hazard ratios (HR). The standardized incidence ratio (SIR) is explained in Breslow (1987) and the standardized mortality ratio (SMR) in Everitt and Skrondal (2010). Schubauer-Berigan *et al.* (2009) describes the calculation of the standardized rate ratio (SRR).

38. Public Health England – guidance publication https://www.gov.uk/government/publications/ionising-radiation-dose-comparisons/ionising-radiation-dose-comparisons

39. Brenner *et al.* (2003), Little (2003), Wakeford (2004), Mullenders *et al.* (2009), Mobbs *et al.* (2011), Preston *et al.* (2013), Shore (2014), Kitahara *et al.* (2015), Mattsson and Nilsson (2015).

40. Authors' summary.

THE JAPANESE LIFESPAN STUDY (LSS)

Table 9. RERF reports on cancer mortality in LSS subjects

**RERF report**	**12**	**13**	**14**
Author	Pierce *et al.* (1996)	Preston *et al.* (2003)	Ozasa *et al.* (2012)
Time period	1950-1990	1950-1997	1950-2003
Solid cancer deaths	7,578	9,335	10,929
ERR/Gy solid cancer	0.29	0.37	0.47
95% confidence intervals	0.23 to 0.35	0.26 to 0.49	0.38 to 0.56

41. The Radiation Effects Research Foundation website www.rerf.jp describes the LSS and lists publications based upon the cohort. Key papers describing solid cancer mortality in the LSS are in Table 9. After the bombs were detonated 60-80,000 people were killed instantly in Hiroshima and another 90-166,000 died in the ensuing 4 months. In Nagasaki there were 22-75,000 instant fatalities and another 60-80,000 deaths in the ensuing months. The LD_{50} dose (at which 50% of the exposed population died) occurred at a radius of 1-1.3km of each blast and later dose reconstruction yielded a bone marrow dose estimate for the LD_{50} of 2.9-3.3 Gy Pierce *et al.* (1996), Preston *et al.* (2003), Wakeford (2004). The key message from the LSS papers is that risk of cancer mortality for people exposed on the day remains elevated 60 years on. The ERR is approximately linear with dose, persistently higher for those who were younger at exposure and approximately doubled for women Ozasa *et al.* (2012). The Osaza report indicated that by 2003 about 525 radiation-associated excess solid cancer deaths had occurred in the LSS cohort. A detailed presentation of cancer incidence by tumour site is provided in Preston *et al.* (2007), and an up to date report of solid cancer incidence including sex-specific analyses and the joint effects of smoking is Grant *et al.* (2017).

42. Stewart and Kneale (2000), Little and Charles (1990), Little (2002b), Tubiana *et al.* (2009). If there were a healthy survivor effect, LSS ERRs would be underestimates of the true values.

43. www.rerf.jp gives further details on cohort structure. The 55,000 individuals situated within 2.5 km of the blast were exposed to levels of radiation of 5 mGy or higher with a mean dose of 200 mGy. The 38,500 people 2.5-10 km from the blast received doses below 5 mGy. The 26,500 people not in the city were unexposed residents of Hiroshima or Nagasaki who were not in either city ('NIC') at the time of the bombings. 85% of the cohort experienced irradiation below the mean level of 200 mGy. Dose distribution of the cohort is given in Table 1 of Ozasa *et al.* (2012).

Table 10. Dose distribution amongst the LSS.

**Weighted Colon Dose (Gy)**	**# Subjects**	**%**
Not in City	26,500	22%
<0.005	38,500	32%
0.005-0.1	30,000	25%
0.1-0.2	6,000	5%
0.2-0.5	6,400	5%
0.5-1	3,400	3%
1-2	1,700	1.5%
>2	600	0.5%
Unknown dose	7,000	6%
TOTAL	120,000	

Concerns have been raised that internal or short-range external exposure from contaminated rainfall over the cities and surrounding areas following the atomic bombings distorts the dose distribution measurements in the LSS Takada *et al.* (1983), Sawada (2007). While data are limited, systematic analyses have failed to find deleterious health effects from rain exposure Sakata *et al.* (2014).

44. Up to date cause of death data is in Ozasa *et al.* (2012). The breakdown of excess deaths attributable to radiation is: ~500 from solid cancer, ~ 100 from leukaemia, and ~400 from non-cancer disease. See Ozasa's Table 9. Detailed analysis of leukaemia mortality is in Richardson *et al.* (2009) and incidence in Hsu *et al.* (2013). The not in city group has been included in some analyses, for instance Sugiyama *et al.* (2014).

45. For a list of Adult Health Study report titles, see http://www.rerf.jp/library/archives_e/ahstitle.html. On cardiovascular disease see Ozasa *et al.* (2017). Also see Shimizu *et al.* (2010) on circulatory disease and Yamada *et al.* (2004) on non-cancer disease.

46. Figure 1 in Ozasa *et al.* (2012) contains ERR per gray estimates for all specific causes of death in the latest LSS analysis. Also see Tables 2a and 2b in Furukawa *et al.* (2010). Hsu *et al.* (2013) report recent data on leukaemia incidence.

47. Ozasa *et al.* (2012).

48. Minamoto *et al.* (2004), Neriishi *et al.* (2007), Shore *et al.* (2010), Little (2013), Nakashima *et al.* (2006) .

49. The ERR/Sv for solid cancer incidence is 1.0 (95% CI: 0.2 to 2.3) for *in utero* exposure. The EAR did not increase with time/age as it did for an equivalent cohort exposed in early childhood, suggesting that lifetime risks following *in utero* exposure may be lower than for early childhood exposure Ohtaki *et al.* (2004), Preston *et al.* (2008). During the first 15 years of life for the *in utero* cohort 1 death and 2 cases of solid cancers were recorded, but no leukaemias Delongchamp *et al.* (1997) Jablon and Kato (1970). A surprising absence of stable chromosome aberrations among intrauterine exposed survivors who had received moderate and high doses (>100 mGy) contrasted with findings for mothers, and suggests high sensitivity of the haematopoietic system *in utero* to cell killing. This may be a reason for the absence of childhood leukaemia in this group. See Ohtaki *et al.* (2004).

50. Schull and Otake (1999), Otake and Schull (1998).

51. Jablon and Kato (1970), Delongchamp *et al.* (1997). The study of children of survivors is known as the F1 cohort. Little *et al.* (1994), Neel and Schull (1991), Nakamura (2006), Otake *et al.* (1990), Tatsukawa *et al.* (2013), Fujiwara *et al.* (2008), Neel *et al.* (1990), Kodaira *et al.* (2004), Kodaira *et al.* (2010), Izumi *et al.* (2003), Satoh *et al.* (1996), Grant *et al.* (2015). For a recent review see Little *et al.* (2013). For further discussion of genetic effects, see UNSCEAR (2010) Section B, ICRP 103 (2007) Annex A and BEIR VII (2006).
52. Authors' summary. The excess relative risk quoted here is different from the nominal cancer risk coefficient of 5.5% per Sv derived by the ICRP and used in optimization calculations. The ERR of 0.47 per gray for solid cancer is an estimate of the amount by which the underlying risk of solid cancer is increased proportionally for each gray of exposure. The ICRP's "nominal risk coefficient" of 5.5% per Sv is an estimate of the health detriment due to cancer experienced in a population exposed to low level radiation; it includes attributable fatal and non-fatal cancer, years of life lost and pain and suffering.

THE CHERNOBYL NUCLEAR POWER PLANT ACCIDENT

53. UNSCEAR (2008)'s Table 1 page 49 describes the accident and documents the principal radionuclides released. The radioactive release from Chernobyl lasted around 10 days, and two radionuclides, the short-lived iodine-131 and the long-lived caesium-137, were particularly significant. The radioactive plume spread over much of the western USSR and Europe. Maps are in Figures I and II in UNSCEAR (2008) and at http://www.unscear.org/unscear/en/chernobylmaps.html.
54. Data from Table 11 is from UNSCEAR (2008) Table 2, page 54. Extensive further details of radiation doses are presented in their Appendix B.
55. Local populations continued to drink milk that had been contaminated with radioactive iodine when cows grazed on contaminated pastures WHO (2006).
56. As of 2016 the most recently published summary figures are in UNSCEAR (2008). A recent update is at http://www.who.int/ionizing_radiation/chernobyl/Chernobyl-update.pdf?ua=1. Table D7 page 189 in UNSCEAR (2008) lists the causes of death of the 19 Chernobyl ARS survivors who died between 1993 and 2004. Of these 19 deaths only the 5 from malignancy are likely to be related to radiation. Literature on childhood thyroid cancer post-Chernobyl is discussed below.
57. The figure of 6,000 cases is given in Volume II, Annex D of UNSCEAR (2008) where Paragraphs 66-73 summarise childhood thyroid cancer as a confirmed effect of Chernobyl radiation exposure. Cardis and Hatch (2011) is a more recent review of published work. See also Cardis *et al.* (2006a), Boice (2005), Zablotska *et al.* (2011), Tronko *et al.* (2006) and Brenner *et al.* (2011). Ivanov *et al.* (2012) report that ERR/Gy is decreasing with time since exposure in a Russian cohort exposed as children. Suzuki and Yamashita (2012) summarises the debate about low dose risk of thyroid cancer, concluding that a statistically significant increase has hardly been described with radiation doses below 100 mSv. A recent pooled analysis of 12 studies of thyroid cancer after childhood exposure to external radiation found a significant increase in RR for doses <0.10 Gy with no significant departure from linearity Veiga *et al.* (2016).
58. Zablotska *et al.* (2013) give the ERR of 1.26. See also Kesminiene *et al.* (2008) and Romanenko *et al.* (2008). Paragraphs D173-D179 in UNSCEAR (2008) summarise a large number of studies of leukaemia in emergency and recovery workers at Chernobyl. Reports of chronic lymphocytic leukaemia in Chernobyl liquidators Zablotska *et al.* (2013) and Kesminiene *et al.* (2008) showed positive non-significant increases in risk. A significant dose response was seen in the LSS cohort based on a simple trend test on 12 eligible cases of CLL, 4 of which occurred among survivors with doses in excess of 0.2 Gy Hsu *et al.* (2013). A significantly increased risk of the incidence of CLL was observed in uranium miners Rericha *et al.* (2006). However, no evidence for an increase in CLL risk has been seen in several other major studies Krestinina *et al.* (2013b), Leuraud *et al.* (2015), Kuznetsova *et al.* (2016). For a review of the open questions surrounding the radiogenicity of chronic lymphocytic leukaemia see Hamblin (2008) and Richardson *et al.* (2005).
59. Parkin *et al.* (1996) describe the largest and most comprehensive study to date, the European Childhood Leukaemia-Lymphoma Study (ECLIS), in particular their Figure 3, page 92, shows the lack of a dose-response relationship between leukaemia incidence and radiation dose. Davis *et al.* (2006) and Noshchenko *et al.* (2002) describe studies showing mixed results regarding leukaemia incidence amongst exposed children in the Ukraine which are criticised regarding selection bias in WHO (2006). Noshchenko *et al.* (2010) found an increase in leukaemia among Ukrainian children exposed at ages 0-5 to more than 10 mSv, but less than that reported by Noshchenko *et al.* (2002), and there are questions about possible biases in control selection UNSCEAR (2013b). Paragraphs D169-D172 in UNSCEAR (2008) review other peer-reviewed publications. Petridou *et al.* (1996) describe a low-powered study that suggested *in utero* radiation exposure from Chernobyl increased risk of infant leukaemia in Greece. Similar studies in Germany were unable to replicate the Greek results Steiner *et al.* (1998). The UK Committees COMARE and CERRIE concluded that there is insufficient evidence to support hypotheses of increased childhood leukaemia in European countries linked to Chernobyl CERRIE (2004) pages 67-68, COMARE (2004). Two members of the CERRIE committee criticized this conclusion of the majority and recorded in the report their belief that the current risk estimates are appreciably in error. Peer-reviewed scientific analyses of this argument have not lent support to this critique. For a review of the debate around risks of internal emitters subsequent to the publication of the ICRP 2007 recommendations, see Harrison and Day (2008).

Table 11. Dose distribution from the Chernobyl accident.

**Population**	**Number**	**Average effective dose (mSv) from external and internal radiation* 1986 - 2005**	**Average thyroid dose (mGy) 1986**
Recovery operation workers	530,000	117.0	-¥
Evacuees	115,000	31.0	490.0
Residents of contaminated areas (>37kBq m^{-2})	6,400,000	9.0	102.0
Residents of Belarus, Russian Fed and Ukraine	98,000,000	1.3	16.0
Other European Residents	500,000,000	0.3	1.3

*Excluding thyroid dose

¥ Data unavailable

60. Reviewed in Cardis and Hatch (2011) and UNSCEAR (2000a) Paragraphs D182 – D199. Ivanov *et al.* (2004), Rahu *et al.* (2006b), Ivanov *et al.* (2008), Rahu *et al.* (2013a), Rahu *et al.* (2013b) all study recovery workers and find non-significant dose-responses. Kashcheev *et al.* (2015) report the ERR/Gy of 0.47 for incidence, but find the studied cohort has lower solid cancer mortality than controls – attributed either to monitoring or a healthy worker effect. It is notable that Kashcheev et al have a longer follow-up than the other studies of solid cancers in highly exposed workers. Ivanov *et al.* (2004), Prysyazhnyuk *et al.* (2007) and Table D19 in UNSCEAR (2008) describe solid cancer risks for exposed (non-worker) population groups.
61. Pukkala *et al.* (2006), Bogdanova *et al.* (2010), Dardynskaia *et al.* (2006).
62. Cotterill et al. (2001) and Tondel et al. (2004) are studies of cancer trends in Western and Northern Europe finding increases of cancer that are attributed to Chernobyl. Cardis et al. (2006b) refute these (in particular their Figure 5, page 1232) with temporal trends in cancer incidence grouped by average dose and age at diagnosis across Europe that found no measurable association between solid cancer trends and the Chernobyl accident. Alinaghizadeh et al. (2014) (with authorship including Tondel) similarly found no measurable effect from Swedish data. Yablokov and Nesterenko (2009) is a book of papers published in Russian and then republished (but not peer-reviewed) in the Annals of the New York Academy of Sciences which contains a number of low-quality analyses and uncorroborated statements that have been misleading for those attempting to gauge the evidence surrounding Chernobyl and radiation impacts. http://www.nyas.org/Publications/Annals/Detail.aspx?cid=f3f3bd16-51ba-4d7b-a086-753f44b3bfc1 gives access to the explicit statement from the journal that the publication is not peer reviewed and links to negative reviews (Charles (2010), Balonov (2012) and Jargin (2010)).
63. Hatch *et al.* (2015), Ostroumova *et al.* (2016).
64. Zablotska *et al.* (2008), Ostroumova *et al.* (2009), Ostroumova *et al.* (2013), Zablotska *et al.* (2015).
65. Worgul *et al.* (2007) is the key cataract risk study which calculated an ERR/Gy of 0.4 (95% CI 0.01 to 2.00). Ivanov *et al.* (2006) and Kashcheev *et al.* (2016) report on cerebrovascular disease in Chernobyl emergency workers. Kashcheev *et al.* (2016) report an increased incidence risk for cerebrovascular diseases (ERR/Gy = 0.45, 95% CI: 0.28 to 0.62). While the Kashcheev study was large, observing over 61,000 workers between 1986 to 2012, it was not able to adjust for known risk factors like weight, smoking and alcohol consumption, and the proportion of workers reported to have been diagnosed with a cerebrovascular disease is surprisingly high (43%).
66. For an assessment concluding that there is no convincing evidence of increased risk of birth defects from exposure to radiation in contaminated areas see UNSCEAR (2001) part VI-A, section 3-4, page 57. See WHO (2006) for data showing that increases in birth defects between 1986 and 1999 in Belarus were not different between contaminated and uncontaminated areas. This WHO study was criticised by some groups for underestimating the impact of low-level radiation on health in general. Holt (2010) gives some perspective.Wertelecki (2010) describes a study showing above average rates of birth defects in the Ukraine including neural tube defects (odds ratio 1.46 (95% CI: 1.13-1.93)) and microcephaly (odds ratio 2.8 (95% CI: 1.15-6.79)). This study lacked data about confounding risk factors such as maternal alcohol intake and diet. Weinberg *et al.* (2001) and Aghajanyan and Suskov (2009) describe genomic abnormalities in children born to exposed individuals. Bridges *et al.* (2013) discuss potential design flaws in these studies. A series of studies describe germline excess minisatellite mutations in a Belarusian population exposed to Chernobyl radiation Dubrova *et al.* (1996), Dubrova *et al.* (1997), Dubrova *et al.* (2002). This phenomenon has not been observed in offspring of Japanese bomb survivors Satoh *et al.* (1996), Asakawa *et al.* (2004), Kodaira *et al.* (2004), Kodaira *et al.* (2010), nor in other Chernobyl-exposed groups Livshits *et al.* (2001), Kiuru *et al.* (2003), Slebos *et al.* (2004), Furitsu *et al.* (2005), nor in Sellafield workers Tawn *et al.* (2015). Little (2015) discusses possible reasons for differences. Mughal *et al.* (2012) suggests that genomic instability in offspring may be triggered only by a dose in excess of a threshold, higher for chronic exposure than acute. The implication for expressed phenotype of increased frequencies of minisatellite mutations is not known Bouffler *et al.* (2006).
67. Cardis *et al.* (1996) give a calculation suggesting that the predicted lifetime excess of cancer and leukaemia deaths due to Chernobyl radiation is 4000 for liquidators, evacuees and residents of the strict control zones, and a further 5000 deaths for the most exposed persons in Belarus, Russia and Ukraine (about 1% of the total numbers of cancers expected in these populations). Cardis *et al.* (2006b) give further extrapolation to European countries and conclude that Chernobyl radiation may eventually be responsible for 16,000 cases of thyroid cancer (95% CI: 3,400 to 72,000) and 25,000 cases of other cancer (95% CI: 11,000 to 59,000). This accounts for around 0.01% of total cancers expected over the time period to 2025. Concerns have been expressed about the calculation of numbers of potential deaths from theoretical risk models (for instance Gonzalez *et al.* (2013)).
68. For reviews finding elevated levels of depression and post-traumatic stress disorder in first responders and clean-up workers, as well as poor quality of life measures amongst the general population, see Kinley (2006),WHO (2006), Bromet *et al.* (2011), Bromet (2012). Rahu *et al.* (2006a) finds an increased risk of suicide amongst 5000 Estonian clean-up workers. Havenaar *et al.* (1996), Havenaar *et al.* (1997) describe a study showing that exposed Belarusian residents had poorer mental health scores than controls. Adams *et al.* (2002)), Adams *et al.* (2011) demonstrate the lasting nature of psychological impacts on evacuees in Kiev. There is no evidence of a dose response effect for psychological effects amongst evacuees or the general population. In one major study the key risk factors were the belief that one's health was affected by Chernobyl, and being diagnosed with a Chernobyl-related health problem Bromet and Havenaar (2007). A study on mental health of Ukrainian clean up workers found that high exposure level (roof workers) was associated with current somatic and post-traumatic stress disorder symptom severity Loganovsky *et al.* (2008).
69. Men *et al.* (2003).
70. Authors' summary.

THE FUKUSHIMA DAI-ICHI NUCLEAR ACCIDENT

71. A brief description of the accident is given in Hasegawa *et al.* (2015) and a longer one in part II (page 28) of UNSCEAR (2013a).
72. Table 2 of Hasegawa *et al.* (2015), summarised in Table 12 below.

Table 12. The distribution of effective radiation doses to workers in the emergency and recovery operations at the Fukushima Dai-ichi nuclear power plant.

Dose (mSv)	Number of Workers
<10	19,198
10-50	8,614
50-100	1,347
100-150	138
>150	35

73. Tanigawa and Chhem (2013).
74. Table 5 page 53 in UNSCEAR (2013a) and Table 3 in Nagataki and Takamura (2016) show estimated effective doses for the first year following the accident for members of the public. Tables 4.4-1 to 4.4-3 in IAEA (2015) present calculated additional lifetime risks for emergency workers. Section 4.4 to 5.3 in the same document describes modelled lifetime risks for members of the public.
75. Measured doses are summarised in Nagataki and Takamura (2016) and Tokonami *et al.* (2012). Table 6 page 57 in UNSCEAR (2013a) shows estimated effective doses to evacuees.
76. UNSCEAR (2013a).
77. Between October 2011 and March 2014 300,476 residents of Fukushima Prefecture aged 18 or under at the time of the accident were screened Suzuki (2016a). The sensitive ultrasound technique revealed 113 cancers or suspected cancers. This number was around 30-fold higher than would have been expected from cancer registry data. Tsuda *et al.* (2016b) attributed the findings to radiation exposure, but this conclusion was vigorously contested Jorgensen (2016), Korblein (2016), Sallmen *et al.* (2016), Shibata (2016), Suzuki (2016b), Takahashi *et al.* (2016), Takamura (2016), Tsuda *et al.* (2016a), Wakeford *et al.* (2016) on multiple grounds. Wakeford (2016) and Suzuki (2016a) enumerate the reasons why this is an incorrect interpretation. There is a well-documented precedent for this pattern in South Korea where the introduction of sensitive ultrasound thyroid screening caused over-diagnosis of thyroid cancer Ahn *et al.* (2014), Ahn and Welch (2015), Williams (2015). A smaller but more directly applicable study of three unexposed Japanese prefectures, which found similar results to those observed iCardis *et al.* (2007)n Fukushima prefecture, is Hayashida *et al.* (2013). See Normile (2016) for an overview.
78. Tanigawa *et al.* (2012). See also an editorial Thomas and Symonds (2016) and reviews of Hasegawa *et al.* (2015) and Hasegawa *et al.* (2016). During evacuation there were over 50 deaths amongst vulnerable populations and in the 3 months after the accident mortality among elderly people at nursing facilities increased three-fold.
79. Authors' summary.

STUDIES OF WORKERS EXPOSED TO RADIATION.

80. Wakeford (2009) gives an overview focussing on occupational exposure.
81.
    a. See Wernli (2016) for a history of individual monitoring and Ainsbury *et al.* (2011) for a review of retrospective dosimetry techniques. The studies' results are presented as ERR/Gy, although some studies include neutron doses in sieverts. The large pooled study, INWORKS, includes studies of worker cohorts in the UK, France and the US. The results for the nuclear worker studies and the LSS are in broad agreement even though the workers usually accumulated their doses over many years, whilst the LSS subjects received theirs in a few seconds – supporting the assumption of additivity and dose-rate independence of radiation doses. There are other key differences between the LSS and worker studies, including exposure to different types of radiation (e.g. gamma rays of different energies), and differences in the demographics, genetics and lifestyle features of the subject population Stewart and Kneale (2000), Little (2002a). The LSS figures for working-age males in Table 4 are as calculated by Cardis *et al.* (2005) and Muirhead *et al.* (2009). INWORKS is described by Richardson *et al.* (2015) for solid cancer risk and Leuraud *et al.* (2015) for leukaemia risk. See Nagataki and Kasagi (2015) and also Doss (2015) for comments and concerns regarding the INWORKS study design. The INWORKS study is a refinement of previously published pooled cohort studies known as the 14- and 15-country studies (the former being the 15-country study minus Canada); results from the 15-country study were found to be affected by historic dose estimate issues in one contributing cohort (i.e. Canada) Cardis *et al.* (2005), Cardis *et al.* (2007), Thierry-Chef *et al.* (2007), Ashmore *et al.* (2010), Wakeford (2014b), Zablotska *et al.* (2014). Japanese nuclear worker data originates from Akiba and Mizuno (2012), where they also discuss the risk of alcohol consumption confounding this result. The ERR/Sv estimate of all cancers excluding leukaemia and alcohol-related cancers in the Japanese study was 0.2 (95% CI -1.42 to 2.09). The Chernobyl Russian clean up worker cohort was analysed by Kashcheev *et al.* (2015). Mayak nuclear worker risks for solid cancers other than lung, liver and bone are from Sokolnikov *et al.* (2015) and for leukaemia from Kuznetsova *et al.* (2016). Rocketdyne data is from Boice *et al.* (2011) and US nuclear power plant workers from Howe *et al.* (2004).
    b. See Stabin and Xu (2014) for an explanation of basic principles in internal radiation dosimetry, focussing on the concept of phantoms. See Gilbert *et al.* (2013) for a Mayak study on lung cancer and Sokolnikov *et al.* (2015) for solid cancer other than lung, liver and bone. Vasilenko *et al.* (2007) details internal dosimetry methods at Mayak. A question of other factors influencing risks in groups of nuclear workers is raised by Gillies and Haylock (2014) and commented on in Boice (2014). A further (environmental) study examining the effects of intake of I-131 from emissions at the Hanford Nuclear Site in the USA is Davis *et al.* (2004).
    c. UNSCEAR (2006a) lists 18 studies of non-cancer disease risks in nuclear workers. Kitahara *et al.* (2015) update this list with a further 3 studies. Little *et al.* (2012) is a systematic review and meta-analysis of circulatory disease risk in 9 studies including 7 nuclear worker studies. Little (2013) is a further review. A series of studies of the Mayak cohort have appeared since the Kitahara update: Moseeva *et al.* (2014), Azizova *et al.* (2015). Azizova *et al.* (2016) report on cataract incidence in the Mayak cohort.
82. See paragraph 81a for references in Table 4.
83. Radiologists.
    a. Wakeford (2004), Mutscheller (1925). A review of the history of dose limits is given by Inkret (1995). Yoshinaga *et al.* (2004) review 8 studies of medical radiation workers. Those studies are of: 6,500 US radiologists (Matanoski *et al.*

(1987); 2,700 UK radiologists Berrington *et al.* (2001); 146,000 US radiological technologists Mohan *et al.* (2003); 6,600 US Army radiological technologists Jablon and Miller (1978); 27,000 Chinese X-ray workers Wang *et al.* (2002); 4,200 Danish radiation therapy workers Andersson *et al.* (1991); 12,200 Japanese radiation technologists Yoshinaga *et al.* (1999) and 73,100 Canadian radiation workers Ashmore *et al.* (1998).

b. Hauptmann *et al.* (2003) describe the circulatory risk for US radiological technicians. Shore (2014) summarises studies from this and three other studies of circulatory disease in medical radiation workers.

c. See Table 3 in Shore (2016b) for a summary that suggests that there is occupational radiation cataract risk amongst medical specialists who receive large cumulative doses (with estimated mean doses from various studies ranging from 0.028 Gy to 6 Gy).

84. 10 Gy is the average alpha dose to the skeleton as a whole: to endosteal surfaces, the putative originating cells for osteosarcoma, the average alpha dose would be about half this. In a US cohort of 820 people there were 46 deaths from bone cancer where less than 1 would have been expected, and a clear excess of cancers of the paranasal sinuses and mastoid air cells was also apparent due to radon formed on the decay of ^{226}Ra in the bones of the head. The equivalent UK workers ingested less radium and experienced 1 bone cancer death against 0.17 expected in a cohort of 1110 individuals. For studies on the US radium dial workers, see Rowland *et al.* (1978), Thomas (1994), Fry (1998) (US overviews), Spiers *et al.* (1983) (US leukaemia), Adams and Brues (1980) (US breast cancer). For studies on the UK radium dial workers, see Baverstock and Papworth (1989) (UK leukaemia) and Baverstock and Vennart (1983) (UK breast cancer). A careful analysis of the data on breast cancer in US radium dial painters suggested that the reported association may have been due to other factors and may not have been causal Stebbings *et al.* (1984).

85. Sigurdson and Ron (2004), Yong *et al.* (2014), dos Santos Silva *et al.* (2013), Hammer *et al.* (2014), Sanlorenzo *et al.* (2015), Shantha *et al.* (2015).

86. A working level (WL) is defined as any combination of the short lived progeny of radon in one litre of air that will result in the emission of 1.3 x 10^5 MeV of potential alpha energy, and a working level month (WLM) is defined as the cumulative exposure from breathing in an atmosphere at a concentration of 1 WL for a working month of 170 hours Tirmarche *et al.* (2010).

a. The 2006 UNSCEAR report combined data from 9 studies comprising over 3,000 lung cancer cases in miners and found an ERR per 100 WLM of 0.59 (95% CI: 0.35 to 1.0), in close agreement with an estimate of 0.49 (95% CI: 0.2 to 1.0) per 100 WLM made 10 years earlier, and a recent assessment concluded that a reasonable summary ERR estimate is 0.5 per 100 WLM. Reviewed in Tirmarche *et al.* (2010) and UNSCEAR (2006b), Annex E. For results from individual studies see Table 21 in UNSCEAR (2006b). For comparisons of meta-analyses see Tirmarche *et al.* (2010) Annex A page 51, UNSCEAR (2006b) Paragraph 427, Lubin (1994), BEIR VI (1999) and Tirmarche *et al.* (2012). A single large study of German Wismut uranium miners (3,016 lung cancer deaths in just under 2 million person-years of follow-up), not included in the earlier pooled analyses, found a smaller risk of 0.19 per 100 WLM (95% CI: 0.16 to 0.22), but when the study was limited to miners with comparatively low cumulative exposures the ERR became 1.3 per 100 WLM (95% CI: 0.7 to 2.1) Walsh *et al.* (2015). Studies of hard rock miners show a *decrease* in ERR per unit of exposure as the rate of exposure *increases,* which may explain differences in results for different studies.

b. Reviewed in Darby *et al.* (1995), UNSCEAR (2006b) Annex E Paragraphs 485 – 491, Tirmarche *et al.* (2010) and (for the German uranium miners) Walsh *et al.* (2015). Also see Kreuzer *et al.* (2015b) and Mohner *et al.* (2010). The ERR of 2.18 is from Kreuzer *et al.* (2016).

c. UNSCEAR (2006a) Annex B, Table 11 summarises results on circulatory disease for 5 studies of miners. Walsh *et al.* (2015) summarise current results for the German uranium miners.

87. Authors' summary.

ENVIRONMENTAL EXPOSURE

88. UNSCEAR (2008), Table 1 in Hughes *et al.* (2005). Bossew *et al.* (2015) map indoor radon across Europe. Because radon is localised in some areas only, exposure prevention is required in such areas known to be affected.

a. Becquerels per metre cubed is strictly an improper (summary) measure: it is practical to use it because it is the ambient concentration that corresponds to a certain dose-rate. Pooled analyses from Europe Darby *et al.* (2006), North America Krewski *et al.* (2006) and China Lubin *et al.* (2004) are summarised in Table 2.2, page 30 of Tirmarche *et al.* (2010). When uncertainties associated with variations in exposure were accounted for, the estimated relative risk in the European pooled analysis increases from 0.08 to 0.16 per 100 Bq/m^3 Darby *et al.* (2006).

b. Figures 2 and 3 in Darby *et al.* (2005) compare risks for smokers and non-smokers. See also the large paper Darby *et al.* (2006). The small study of Torres-Duran *et al.* (2014) reviews studies of residential radon and lung cancer risk in never smokers.

c. See Raaschou-Nielsen *et al.* (2008) for the study of acute lymphoblastic leukaemia in Denmark and Kendall *et al.* (2013) for the study in the UK.

89. Radiation risks in areas with high natural background radiation are reviewed by Hendry *et al.* (2009) Boice (2010) and Aliyu and Ramli (2015). For Kerala results see Nair *et al.* (2009) and for Yangjiang results see Tao *et al.* (2012), the confidence intervals for Tao et al are calculated in Shore (2014). The results of these studies are not statistically significant, although they are of comparable size and dose to some of the worker studies that have identified positive, statistically significant estimates of ERR/Gy. Nevertheless, there is no statistical inconsistency between these estimates, although the Kerala risk estimate is close to statistical incompatibility with the LSS risk estimate.

90. A case-control study in Great Britain based on >27,000 cases from the National Registry of Childhood Tumours compared risks of childhood leukaemia and other cancers with cumulative dose through exposure to indoor gamma radiation and radon based on the mother's address at the time of the child's birth Kendall *et al.* (2013). It found a statistically significant relationship between dose from naturally occurring gamma radiation and the risk of childhood leukaemia, with an ERR/Sv = 120 (95% CI: 30 to 220). Radon exposure did not predict childhood leukaemia, and other childhood cancers were not related to either radon or gamma radiation exposure. UNSCEAR (2013b) cautions that there are large

uncertainties associated with this study with respect to its use of an ecological measure of dose. A census-based cohort study of childhood cancer in Switzerland (with 1,800 incident cases) reported positive relationships between cumulative dose of external radiation and both childhood leukaemia (ERR/Sv = 50 (95% CI: 0 to 100)) and central nervous system cancers (ERR/Sv = 50 (95% CI: 0 to 110)) Spycher *et al.* (2011). A Finnish case-control study (1093 cases) with full residential history found a non-significant odds ratio increase for childhood leukaemia with increasing dose-rate of background radiation, with a significantly elevated odds ratio in the age group 2-7 years Nikkila *et al.* (2016). These relationships between exposure to background gamma radiation and childhood leukaemia incidence are broadly comparable to those from the LSS, lending some support to the application of risk estimates derived from the LSS to the very low dose-rates received from naturally occurring background gamma radiation. However, a French census-based analysis with 9,056 incident cases over 20 years found no evidence of an association of childhood leukaemia risk with either radon (SIR by 100 Bq/m^3 1.01, 95% CI 0.91 to 1.12) or gamma radiation (SIR by 10 nSv/h 1.01, 95% CI 1.00 to 1.02) Demoury *et al.* (2016).

91. For Techa River residents see Schonfeld *et al.* (2013) and Davis *et al.* (2015) for solid cancer, Krestinina *et al.* (2013b) for leukaemia and Krestinina *et al.* (2013a) for cardiovascular disease.

92. Cancer risks due to fallout are reviewed in, for example, Simon *et al.* (2006), Simon and Bouville (2015). The analysis of 11 cancer registries is reported in Wakeford *et al.* (2010) and the study focussing on the Nordic countries is reported in Darby *et al.* (1992). The relative risk of leukaemia for ages 0-14 in the high exposure period versus the medium exposure period was 1.07 (95% CI: 1.00 to 1.14). Other populations exposed during fallout include the Marshall Islanders Land *et al.* (2010), Simon *et al.* (2010), inhabitants of Utah near to the Nevada Test Site Stevens *et al.* (1990) and inhabitants of Semipalatinsk in Kazakhstan Abylkassimova *et al.* (2000), Akleyev (2007). See Wakeford (2014a) for a discussion of fallout in the context of discharges from nuclear installations.

93. Residential areas around nuclear facilities.

a. The epidemiology of childhood leukaemia near nuclear installations has been reviewed in Laurier *et al.* (2008). See also Laurier *et al.* (2014), Kinlen (2011), Janiak (2014) and COMARE (2011). Wakeford (2014a) briefly summarises the history of investigations of childhood leukaemia near Sellafield and Dounreay. Bunch *et al.* (2014) report recent follow-up data from the same populations which have not exhibited excess cases of leukaemia since the early 1990s. The cluster near Krummel was first reported by Schmitz-Feuerhake *et al.* (1993). COMARE (2011) comprehensively reviews studies of the risk of leukaemia in young people living in the vicinity of nuclear power plants in Great Britain and other countries in Chapter 3 and in Germany in Chapter 4. COMARE (2016) reviews the incidence of childhood cancer around the Sellafield and Dounreay nuclear installations with data up to 2006 concluding that in the time period 1991-2006 the incidence rates of leukaemia and non-Hodgkins lymphoma had reduced to unexceptional levels in both locations.

b. The KiKK study was a German case control study of cancer diagnosed in children below the age of 5. Its main finding was a statistically significant positive association between the risk of leukaemia before 5 years of age and living less than 5km from a nuclear power plant Kaatsch *et al.* (2008a). A commentary by Little *et al.* (2008a) gives context. Chapter 4 of COMARE (2011) summarises further descriptions and analyses of the KiKK study. Additional analysis of the KiKK data compared observed and expected numbers of cases in the same group of children (leukaemia below 5 years of age and living within 5km of a nuclear power plant in Germany) and reported a standardised incidence ratio (SIR) not significantly different from 1 Kaatsch *et al.* (2008b). The KiKK case-control study design was repeated in France and Britain (set in context in Muirhead (2013)). The geographical approach was also repeated for the same risk group in France, Britain and Switzerland. Neither case-control study recapitulated the odds ratio (OR) significantly different from 1 and just one time interval from the French geographical study generated an SIR marginally significantly different from 1. When the French study broadened the age group under consideration to include children under 15, a marginally significant result was observed (see Table 2, Sermage-Faure *et al.* (2012)). Table 13 summarises numerical values of ORs and SIRs from these studies and gives references.
Other recent studies in Finland (2 NPPs (nuclear power plants)), Canada (3 NPPs) and Belgium (5 NPPs) report non-significant SIRs for children below 15 years with various definitions of residing close to NPPs Heinavaara *et al.* (2010), Bollaerts (2012), Lane (2013). Table 5.1, page 59 in COMARE (2011) calculates an SIR from a meta-analysis of older (pre-2009) data for children under 5 years "in the vicinity" of 80 nuclear power plants in 5 countries. The resulting SIR is 1.07 (0.92 to 1.26).

Table 13. Childhood leukaemia in children < 5 years old living < 5 km from a nuclear power plant published as the KiKK study and since. N is the number of cases of leukaemia in children below 5 years of age resident < 5km from a nuclear power plant (NPP).

**Country**	**OR from case control studies**	**SIR from ecological and cohort studies**
Germany	2.19 lower 95% CL = 1.51 1980-2003<br>N=37<br>Kaatsch *et al.* (2008a)	1.41 (0.98 to 1.97) 1980 - 2003<br>N = 34<br>Kaatsch *et al.* (2008b)
France	1.6 (0.7 to 4.1) 2002 - 2007<br>N=6<br>Sermage-Faure *et al.* (2012)	2.2 (1.0 to 4.4) 2002 – 2007<br>N=8<br>1.4 (0.8 to 2.3) 1990 – 2007<br>N=14<br>Sermage-Faure *et al.* (2012)
Britain	0.86 (0.49 to 1.52) 1962 - 2007<br>N = 10<br>Bithell *et al.* (2013)	1.22 (0.75 to 1.89) 1969 - 2004<br>N=20<br>COMARE (2011)
Switzerland		Incidence Rate Ratio<br>1.2 (0.6 to 2.41) 1985 - 2009<br>N = 8<br>Spycher *et al.* (2011)

c. See Black (1984) for Sellafield, COMARE (1999) for Dounreay, SSK (2008) for KiKK.

d. COMARE (2006) found that childhood leukaemia in Britain tends to cluster, but this is not a consistent result across all such studies. For example Alexander (1998) finds evidence of clustering in a dataset of 13,351 cases of childhood leukaemia from 17 countries, whilst Schmiedel *et al.* (2010) found no evidence of a tendency to clustering amongst 11,946 cases of childhood leukaemia in Germany. The town of Fallon in Nevada USA had an unusually high incidence of childhood leukaemia (14 cases) during the years 1997–2003 Francis *et al.* (2012). The town is not near a nuclear installation and the cause of the cluster remains unknown.

e. Kinlen (1988) first proposed the population mixing hypothesis. Kinlen (2012) presents a review and meta-analysis of 20 years' data on childhood leukaemia and population mixing. Lupatsch *et al.* (2015) describe a Swiss cohort study in which population mixing did not predict the risk of childhood leukaemia, but see Kinlen's letter of response Kinlen (2015) and also Lupatsch *et al.* (2016) which found an association between Swiss population growth and childhood leukaemia.

f. Gardner *et al.* (1990) proposed paternal pre-conceptional radiation as the cause of the cluster near Sellafield. Doll *et al.* (1994), COMARE (2002) and COMARE (1999) summarise the evidence against the hypothesis. Recent reviews are Wakeford (2013), Wakeford (2014a).

g. Fairlie (2014), CERRIE (2004), Wakeford (2014a).

94. Industrial processes such as the burning of coal, the production of phosphate fertilizers and the extraction of oil and gas have the potential to increase exposure to naturally occurring radioactive materials (NORMs) and hence to elevated exposure in workers and in the environment. Individuals in such industries have received less scrutiny than other exposed individuals described here. Doyi *et al.* (2016) IAEA (2003) and http://www.world-nuclear.org/information-library/safety-and-security/radiation-and-health/naturally-occurring-radioactive-materials-norm.aspx

95. Authors' summary.

MEDICAL EXPOSURE

96. Dose fractionation (in which the total dose is delivered as a number of doses separated in time) allows the optimization of the lethal effect on diseased cells while sparing healthy tissues. There are known risks from such therapy which have to be balanced against the benefits of treating the underlying disease. There is a large body of data on those risks which is growing as radiotherapy becomes more successful and people survive ever longer after their radiotherapy. These data have to be treated with caution as individuals treated with radiotherapy are already patients, so they are not a representative sample of the general population, and this could affect estimates of radiation risks. Further, radiotherapy is usually focused on localised diseased tissues, leading to a highly heterogeneous distribution of doses within the body. There is an overall pattern that the ERR/Gy from radiotherapy tends to be lower than the corresponding values in the LSS. This pattern is more marked at higher average radiotherapy dose and is therefore thought to be explained by spatially-focussed radiation used in therapy killing a large proportion of cells that might otherwise have become cancerous due to irradiation – the so called sterilization effect. However these two patterns are not ubiquitous: some individual radiotherapy studies have a higher ERR/Gy than the corresponding values in the LSS. Little (2001) reviews 116 radiotherapy studies and compares ERRs for incidence and mortality with comparable risks in the LSS. Travis *et al.* (2003a) found that radiation related risk remained high even at the highest doses in women < 30 years of age treated for Hodgkin disease with radiotherapy (i.e., there was no evidence of a sterilization effect). Wakeford (2004) reviews cancer epidemiology amongst medically irradiated groups. Two of the largest studies are of 14,000 ankylosing spondylitis patients Weiss *et al.* (1994), Weiss *et al.* (1995)and 80,000 women treated for cervical cancer Boice *et al.* (1985), Boice *et al.* (1987), Boice *et al.* (1988). Little (2016) reviews risks from therapeutic and diagnostic doses, and Tran *et al.* (2017) updates analyses for two diagnostically treated groups.

97. For studies on Ra-224 see Wick *et al.* (1999), Nekolla *et al.* (2000). For Thorotrast studies see Travis *et al.* (1992), Travis *et al.* (2001), Travis *et al.* (2003b).

98. Linet *et al.* (2012) review risks from diagnostic imaging and their Table 4 gives estimates for dose from various different examinations.

a. Bithell and Stewart (1975) describe the OSCC, Wakeford (2008) reports pooled results from 32 smaller studies of X-rays *in utero*. See Doll and Wakeford (1997) for dose estimates and comparisons with risks from post-natal radiation, and also Preston *et al.* (2008) for a study on the LSS.

b. Linet *et al.* (2012) discuss the wide range of results of studies of X-rays in children and adults, and why they might be so variable. Table 2 in Shore (2014) summarises leukaemia risks from larger studies of medical exposures. Little and Boice (1999) compare breast cancer risks in fluoroscopy patients and the LSS. Ronckers *et al.* (2010) examines breast cancer and scoliosis. Howe (1995) reports strictly null dose-response results for lung cancer risk after multiple fluoroscopic examinations.

c. A UK study calculated absorbed dose from CT scans to the red bone marrow and the brain and found an ERR/Gy = 37 (95% CI: 6 to 121) for leukaemia and ERR/Gy = 24 (95% CI: 11 to 47) for brain tumours Pearce *et al.* (2012). The equivalent values from the LSS based on age at exposure and follow up time were an ERR/Sv of 45 (95% CI: 16 to 188) for leukaemia and an ERR/SV of 6.1 (95% CI: 0.1 to 64) for brain tumours. An Australian study reported: an ERR/Gy = 39 (95% CI: 14 to 70) for leukaemia, using bone marrow dose; an ERR/Gy = 21 (95% CI: 14 to 29) for brain cancer after brain CT using dose to the brain; and an ERR/Sv = 27 (95% CI: 17 to 37) for solid cancer (excluding brain cancer after brain CT) using effective dose Mathews *et al.* (2013). See, for example Brenner (2014), Walsh *et al.* (2014), Journy *et al.* (2015) for discussion of these results. A French study Journy *et al.* (2015) that attempted to take into account predisposing factors was shown to suffer from some methodological limitations Cardis and Bosch de Basea (2015), Muirhead (2015). A further overview is Boice (2015).

99. Authors' summary.

EXPERIMENTAL STUDIES OF MECHANISMS OF DAMAGE

100. Valentin (2005), page 11 and pages 313-315 of BEIR VII (2006), Annex A2 of ICRP 103 (2007), UNSCEAR (2012a) and NCRP (2015) all offer reviews of radiobiology. Complex DSB are a

combination of strand breaks and base damages all within a few nanometres along the DNA, containing at least one break on each strand of the DNA (hence "DSB") and at least one more break and/or base damage (hence "complex"). These are illustrated in Figure 5 of Goodhead (2009). Goodhead (1994) and Lomax *et al.* (2013) give general introductory explanations of the generation and relevance of clustered damage. Choi *et al.* (2015) give an explanation of indirect damage.

101. Molecular mechanisms of DNA repair are reviewed in pages 32-39 of BEIR VII (2006) and in Shibata and Jeggo (2014). Molecular mechanisms of DNA repair including a consideration of damage complexity are reviewed in Moore *et al.* (2014). At high doses the linear quadratic dose response saturates for counts of chromosome aberrations (e.g. for human lymphocytes saturation occurs at 4-5 Gy).
102. On checkpoints see ICRP 99. Lobrich and Jeggo (2007), Deckbar *et al.* (2007) and Fernet *et al.* (2010) discuss the threshold below which the G2/M checkpoint does not operate. Martin *et al.* (2013) review low dose hypersensitivity.
103. Hlatky *et al.* (2002) and Goodhead (2009) describe diversity in chromosome aberrations. Annex A, Paragraph A45 of ICRP 103 (2007) on radiation-associated tumours states "evidence for the presence of specific mutational signatures of radiation is currently lacking". The European research initiative DoReMi has this research area as one of its main priorities Salomaa *et al.* (2015). Pernot *et al.* (2012) provides a comprehensive review of attempts to identify biomarkers that would be of use in the epidemiology of radiation risk. There is current research into biomarkers for thyroid cancer Dom *et al.* (2012), Suzuki *et al.* (2015) and in experimental systems Sherborne *et al.* (2015).
104. The multistep model of carcinogenesis was proposed by Armitage and Doll (1954) and is supported by molecular data from human colon cancers Vogelstein *et al.* (1988). For a comprehensive review of cancer biology see Chapter 18 (p273) in Hall and Giaccia (2011). For a recent review see Mullenders *et al.* (2009).
105. Stem cell biology and its implications for radiological protection are discussed by Hendry *et al.* (2016). The important role of stem cells in carcinogenesis generally is supported by the recent observations of a strong correlation between cancer incidence and the number of lifetime stem cell divisions Tomasetti *et al.* (2017).
106. Adaptive responses appear to involve transcriptional modulation of specific gene sets Tapio and Jacob (2007). See Mullenders *et al.* (2009) for in vivo studies showing evidence of adaptive response, and Wolff (1996) on adaptive responses to very low radiation doses.
107. So-called "non-targeted effects" are reviewed by Morgan (2003a), Morgan (2003b). UNSCEAR (2012a) reviews more recent evidence. Little (2010) considers the shape of the dose response in light of non-targeted effects. Not all authors would categorise the adaptive response and genomic instability as non-targeted effects, but they are included in Little's 2010 review. A contrasting review of non-targeted effects is Hei *et al.* (2011).
108. Ding *et al.* (2005), Hauptmann *et al.* (2016) and Wahba *et al.* (2017) describe qualitative differences in cellular responses to low dose radiation. The proteomics of low dose radiation is reviewed in Leszczynski (2014). Brooks *et al.* (2016) reviews molecular and cellular events after exposure to ionizing radiation at low dose-rate.
109. Preston (2017) and Ruhm *et al.* (2017) explain how the combination of biological and epidemiological data should, in time, allow development of a data-driven model of the dose-response curve at low dose and low dose-rate. The Euratom project DoReMi has produced a substantial body of literature towards a better understanding of the biological effects of ionizing radiation at low dose and low dose-rates. The project is described in Belli *et al.* (2011), Aerts *et al.* (2014), Belli *et al.* (2015) and the project's publications with their abstracts are listed at www.melodi-online.eu/DoReMi/Publications.html.
110. Animal studies of carcinogenesis directly illustrate the diversity of dose response curves for different cancers. See paragraph 118.
111. Premature cellular senescence after low dose-rate radiation is described in Yentrapalli *et al.* (2013b), Yentrapalli *et al.* (2013a), Rombouts *et al.* (2014).
112. A comprehensive review of radiation specific biomarkers from 2012 Pernot *et al.* (2012) has recently been updated Hall *et al.* (2017) emphasising the potential of a systems biology approach to integrate the rapidly growing "omics" into a mechanistic understanding.
113. Bouffler (2016) reviews variation in individual radio-sensitivity. Individual sensitivity was one of the foci of the Euratom DoReMi research programme Belli *et al.* (2011), Aerts *et al.* (2014), Belli *et al.* (2015), with resulting advances in understanding of susceptibility due to genetic and epigenetic mechanisms Sagne *et al.* (2013), Flockerzi *et al.* (2014), Gurtler *et al.* (2014), Pernot *et al.* (2014), Sagne *et al.* (2014), Schanz *et al.* (2014). An ATM mutation is emerging as a relative contraindication for radiotherapy. For example, ATM mutations in female breast cancer patients predict for an increase in radiation-induced late effects Iannuzzi *et al.* (2002). A-T heterozygotes may comprise 1% of the population, 4% of the cancer population and up to 14% of the breast cancer population Swift *et al.* (1991). The emerging field of radiogenomics aims to understand genetic risk factors for adverse reactions to radiotherapy (for a review see Roberson *et al.* (2016)) .
114. Biological mechanisms whereby ionizing radiation causes cardiovascular disease have been reviewed recently in Stewart (2012), Baselet *et al.* (2016) and Boerma *et al.* (2016). Atherosclerosis is described in Hansson and Hermansson (2011). Animal models have contributed to our understanding of the role of inflammation Monceau *et al.* (2013), Mathias *et al.* (2015) and also of the disruption of cellular organization Barjaktarovic *et al.* (2013) in low dose radiation damage to the heart.
115. Ainsbury *et al.* (2016) offer a recent review on mechanisms of cataract induction by ionizing radiation. Genomic damage is specifically discussed in Worgul *et al.* (1989), oxidative stress in Hamada (2016) and downstream effects including cell division in Jacob *et al.* (2012).
116. Authors' summary.

EXPERIMENTAL STUDIES THAT INFORM RISK ASSESSMENT

117. See, for example, Peacock *et al.* (2000) and Brooks *et al.* (2009) with reviews in Dauer *et al.* (2010) and Morgan and Bair (2013). Figure 2.4 in BEIR VII (2006) (p59) illustrates abnormal chromosome count data as a function of dose from studies of thousands of cells exposed up to 50 mGy. The

original data sources are Pohl-Ruling *et al.* (1983) and Lloyd *et al.* (1992). For studies examining values of DDREF see Lloyd *et al.* (1992), Thacker (1992), UNSCEAR (2000b), Cornforth *et al.* (2002b).

118. Figure 3b in Haley *et al.* (2015) reviews dose response curves from 11 animal carcinogenesis experiments. These experiments and others are described on p73-4 of BEIR VII. The original data for leukaemia in mice is in Bouffler *et al.* (1996b), Bouffler *et al.* (1996a), Bouffler *et al.* (1997); for mammary cancer in mice in Ullrich *et al.* (1987); and for mammary cancer in rats in Shellabarger *et al.* (1980). Radiation induced skin cancer in mice and rats is reviewed in Coggle and Williams (1990).
119. Figures 5 in Haley *et al.* (2015) reviews life shortening data from 16 mouse studies. Life shortening studies in dogs are reviewed in Thompson *et al.* (1989) and Muggenburg *et al.* (2008).
120. Section A16, page 217 in ICRP 103 (2007) describes how the risk estimates for human heritable disease were calculated. Searle (1974) and Sankaranarayanan and Chakraborty (2000) review the large body of data on mutagenesis studies in mice whilst Nakamura *et al.* (2013) compare human and animal data.
121. ICRP (2003), ICRP 103 (2007).
122. Pages 246-250 of BEIR VII (2006) describe the methodology and results for calculating DDREF from animal experiments and human epidemiological data combined. Haley *et al.* (2015) performed an equivalent analysis with a larger database of animal experiments and a different methodology and challenged the BEIR estimate. Different radiation protection organisations use different measures of DDREF. ICRP used a different method and considered that a value of 2.0 was most appropriate for radiation protection purposes ICRP 99 (2005). There is a growing indication that the LDEF and DREF components of DDREF may differ Niwa (2010) with higher values suggested for DREF, e.g. Paunesku *et al.* (2017). Shore *et al.* (2017) conducted a meta-analysis of low dose-rate epidemiologic studies that provide dose-response estimates of total solid cancer risk in adulthood in comparison to corresponding acutely-exposed atomic bomb survivor risk, in order to estimate a dose rate effectiveness factor (DREF) of between 1 and 2. Ruhm *et al.* (2015) describe the historical development of the DDREF concept in light of emerging scientific evidence on dose and dose-rate effects, summarises the conclusions recently drawn by a number of international organisations, mentions current scientific efforts to obtain more data on low dose and low dose-rate effect effects at molecular, cellular, animal and human levels, and discusses future options to improve and optimize the DDREF concept for the purpose of radiological protection.
123. Authors' summary.

PERSPECTIVES

124.
   a. Smith (2007), Cologne and Preston (2000).
   b. Lim *et al.* (2013). For individuals in situations where radiation levels are high, radon dose represents a large risk and exposure prevention measures are well justified.

125. Smith (2007), Cologne and Preston (2000) Lim *et al.* (2013).
126. Authors' summary. Small health risks of radiation are due in part to the active prevention policies which have been implemented and improved for decades in both industry and medicine. Clarke and Valentin (2009), Figure 1 in Inkret (1995).

1. Abbot A. 2015 Researchers pin down risks of low-dose radiation. *Nature* **523**. (doi:10.1038/523017a).

2. Abylkassimova Z., Gusev B., Grosche B., Bauer S., Kreuzer M., Trott K. 2000 Nested case-control study of leukemia among a cohort of persons exposed to ionizing radiation from nuclear weapon tests in Kazakhstan (1949-1963). *Ann Epidemiol* **10**, 479.

3. Adams E.E., Brues A.M. 1980 Breast cancer in female radium dial workers first employed before 1930. *Journal of Occupational and Environmental Medicine* **22**, 583-587.

4. Adams R.E., Bromet E.J., Panina N., Golovakha E., Goldgaber D., Gluzman S. 2002 Stress and well-being in mothers of young children 11 years after the Chornobyl nuclear power plant accident. *Psychol Med* **32**, 143-156.

5. Adams R.E., Guey L.T., Gluzman S.F., Bromet E.J. 2011 Psychological well-being and risk perceptions of mothers in Kyiv, Ukraine, 19 years after the Chornobyl disaster. *Int J Soc Psychiatry* **57**, 637-645. (doi:10.1177/0020764011415204).

6. Aerts A.M., Impens N.R., Baatout S., Benotmane M.A., Camps J., Dabin J.M., Derradji H., Grosche B., Horemans N., Jourdain J.R., et al. 2014 Joint research towards a better radiation protection-highlights of the Fifth MELODI Workshop. *J Radiol Prot* **34**, 931-956. (doi:10.1088/0952-4746/34/4/931).

7. Aghajanyan A., Suskov I. 2009 Transgenerational genomic instability in children of irradiated parents as a result of the Chernobyl Nuclear Accident. *Mutat Res* **671**, 52-57. (doi:10.1016/j.mrfmmm.2009.08.012).

8. Ahn H.S., Kim H.J., Welch H.G. 2014 Korea's thyroid-cancer "epidemic"--screening and overdiagnosis. *N Engl J Med* **371**, 1765-1767. (doi:10.1056/NEJMp1409841).

9. Ahn H.S., Welch H.G. 2015 South Korea's thyroid-cancer "epidemic"--Turning the Tide. *N Engl J Med* **373**, 2389-2390. (doi:10.1056/NEJMc1507622).

10. Ainsbury E.A., Bakhanova E., Barquinero J.F., Brai M., Chumak V., Correcher V., Darroudi F., Fattibene P., Gruel G., Guclu I., et al. 2011 Review of retrospective dosimetry techniques for external ionising radiation exposures. *Radiat Prot Dosimetry* **147**, 573-592. (doi:10.1093/rpd/ncq499).

11. Ainsbury E.A., Barnard S., Bright S., Dalke C., Jarrin M., Kunze S., Tanner R., Dynlacht J.R., Quinlan R.A., Graw J., et al. 2016 Ionizing radiation induced cataracts: Recent biological and mechanistic developments and perspectives for future research. *Mutat Res* **770**, 238-261. (doi:10.1016/j.mrrev.2016.07.010).

12. Akiba S., Mizuno S. 2012 The third analysis of cancer mortality among Japanese nuclear workers, 1991-2002: estimation of excess relative risk per radiation dose. *J Radiol Prot* **32**, 73-83. (doi:10.1088/0952-4746/32/1/73).

13. Akleyev A. 2007 The local health impacts of atmospheric nuclear weapons testing. Cancer epidemiology in areas adjacent to the Semipalatinsk test site, Kazakhstan. *Radiat Environ Biophys* **46**, 297-298.

14. Alexander F. 1998 Clustering of childhood acute leukaemia - The EUROCLUS project. *Radiat Environ Biophys* **37**, 71-74. (doi:10.1007/s004110050096).

15. Alinaghizadeh H., Tondel M., Walinder R. 2014 Cancer incidence in northern Sweden before and after the Chernobyl nuclear power plant accident. *Radiat Environ Biophys* **53**, 495-504. (doi:10.1007/s00411-014-0545-6).

16. Aliyu A.S., Ramli A.T. 2015 The world's high background natural radiation areas (HBNRAs) revisited: A broad overview of the dosimetric, epidemiological and radiobiological issues. *Radiation Measurements* **73**, 51-59. (doi:10.1016/j.radmeas.2015.01.007).

17. Andersson M., Engholm G., Ennow K., Jessen K.A., Storm H.H. 1991 Cancer risk among staff at 2 radiotherapy departments in Denmark. *British Journal of Radiology* **64**, 455-460.

18. Armitage P., Doll R. 1954 The age distribution of cancer and a multi-stage theory of carcinogenesis. *Br J Cancer* **8**, 1-12.

19. Asakawa J., Kuick R., Kodaira M., Nakamura N., Katayama H., Pierce D., Funamoto S., Preston D., Satoh C., Neel J.V., et al. 2004 A genome scanning approach to assess the genetic effects of radiation in mice and humans. *Radiat Res* **161**, 380-390.

20. Ashmore J.P., Krewski D., Zielinski J.M., Jiang H., Semenciw R., Band P.R. 1998 First Analysis of Mortality and Occupational Radiation Exposure based on the National Dose Registry of Canada. *American Journal of Epidemiology* **148**, 564-574.

21. Ashmore J.P., Gentner N.E., Osborne R.V. 2010 Incomplete data on the Canadian cohort may have affected the results of the study by the International Agency for Research on Cancer on the radiogenic cancer risk among nuclear industry workers in 15 countries. *J Radiol Prot* **30**, 121-129. (doi:10.1088/0952-4746/30/2/001).

22. Auvinen A., Kivelä T., Heinävaara S., Mrena S. 2015 Eye Lens Opacities Among Physicians Occupationally Exposed to Ionizing Radiation. *Annals of Occupational Hygiene*. (doi:10.1093/annhyg/mev022).

23. Azizova T., Grigorieva E., Hunter N., Pikulina M., Moseeva M. 2015 Risk of mortality from circulatory diseases in Mayak workers cohort following occupational radiation exposure. *J Radiol Prot* **35**, 517.

24. Azizova T.V., Bragin E.V., Hamada N., Bannikova M.V. 2016 Risk of Cataract Incidence in a Cohort of Mayak PA Workers following Chronic Occupational Radiation Exposure. *PLoS One* **11**, e0164357. (doi:10.1371/journal.pone.0164357).

25. Balonov M.I. 2012 On protecting the inexperienced reader from Chernobyl myths. *J Radiol Prot* **32**, 181-189. (doi:10.1088/0952-4746/32/2/181).

26. Barjaktarovic Z., Anastasov N., Azimzadeh O., Sriharshan A., Sarioglu H., Ueffing M., Tammio H., Hakanen A., Leszczynski D., Atkinson M.J., et al. 2013 Integrative proteomic and microRNA analysis of primary human coronary artery endothelial cells exposed to low-dose gamma radiation. *Radiat Environ Biophys* **52**, 87-98. (doi:10.1007/s00411-012-0439-4).

27. Baselet B., Rombouts C., Benotmane A.M., Baatout S., Aerts A. 2016 Cardiovascular diseases related to ionizing radiation: The risk of low-dose exposure (Review). *Int J Mol Med* **38**, 1623-1641. (doi:10.3892/ijmm.2016.2777).

28. Baverstock K., Papworth D. 1989 *The UK Radium Luminizer Survey*. UK, British Institute of Radiology.

29. Baverstock K.F., Vennart J. 1983 A note on radium body content and breast cancer in U.K. radium luminisers. *Health Phys* **44 Suppl 1**, 575-577.

30. Behjati S., Gundem G., Wedge D.C., Roberts N.D., Tarpey P.S., Cooke S.L., Van Loo P., Alexandrov L.B., Ramakrishna M., Davies H., et al. 2016 Mutational signatures of ionizing radiation in second malignancies. *Nat Commun* **7**, 12605. (doi:10.1038/ncomms12605).

31. BEIR VI (Biological Effects of Ionizing Radiation). 1999 Health Effects of Exposure to Radon. Washington (DC), National Academies Press.

32. BEIR VII (Biological Effects of Ionizing Radiation). 2006 Phase II Report Health Risks from Exposure to Low Levels of Ionizing Radiation. Washington (DC), National Academies Press.

33. Belli M., Salomaa S., Ottolenghi A. 2011 MELODI: the 'Multidisciplinary European Low-Dose Initiative'. *Radiat Prot Dosimetry* **143**, 330-334. (doi:10.1093/rpd/ncq392).

34. Belli M., Tabocchini M.A., Jourdain J.R., Salomaa S., Repussard J. 2015 The European initiative on low-dose risk research: from the HLEG to MELODI. *Radiat Prot Dosimetry* **166**, 178-181. (doi:10.1093/rpd/ncv136).

35. Berrington A., Darby S.C., Weiss H.A., Doll R. 2001 100 years of observation on British radiologists: mortality from cancer and other causes 1897-1997. *Br J Radiol* **74**, 507-519.

36. Bithell J.F., Stewart A.M. 1975 Pre-natal irradiation and childhood malignancy: a review of British data from the Oxford Survey. *Br J Cancer* **31**, 271-287.

37. Bithell J.F., Murphy M.F.G., Stiller C.A., Toumpakari E., Vincent T., Wakeford R. 2013 Leukaemia in young children in the vicinity of British nuclear power plants: a case-control study. *British Journal of Cancer* **109**, 2880-2885. (doi:10.1038/bjc.2013.560).

38. Black D. 1984 Investigation of the possible increased incidence of cancer in West Cumbria: report of the Independent Advisory Group. London, HMSO.

39. Bodgi L., Canet A., Pujo-Menjouet L., Lesne A., Victor J.M., Foray N. 2016 Mathematical models of radiation action on living cells: From the target theory to the modern approaches. A historical and critical review. *J Theor Biol* **394**, 93-101. (doi:10.1016/j.jtbi.2016.01.018).

40. Boerma M., Sridharan V., Mao X.W., Nelson G.A., Cheema A.K., Koturbash I., Singh S.P., Tackett A.J., Hauer-Jensen M. 2016 Effects of ionizing radiation on the heart. *Mutat Res* **770**, 319-327. (doi:10.1016/j.mrrev.2016.07.003).

41. Bogdanova N.V., Antonenkova N.N., Rogov Y.I., Karstens J.H., Hillemanns P., Dork T. 2010 High frequency and allele-specific differences of BRCA1 founder mutations in breast cancer and ovarian cancer patients from Belarus. *Clin Genet* **78**, 364-372. (doi:10.1111/j.1399-0004.2010.01473.x).

42. Boice J.D., Day N.E., Andersen A., Brinton L.A., Brown R., Choi N.W., Clarke E.A., Coleman M.P., Curtis R.E., Flannery J.T., et al. 1985 Second cancers following radiation treatment for cervical cancer. An international collaboration among cancer registries. *J Natl Cancer Inst* **74**, 955-975.

43. Boice J.D., Blettner M., Kleinerman R.A., Stovall M., Moloney W.C., Engholm G., Austin D.F., Bosch A., Cookfair D.L., Krementz E.T., et al. 1987 Radiation dose and leukemia risk in patients treated for cancer of the cervix. *J Natl Cancer Inst* **79**, 1295-1311.

44. Boice J.D., Engholm G., Kleinerman R.A., Blettner M., Stovall M., Lisco H., Moloney W.C., Austin D.F., Bosch A., Cookfair D.L., et al. 1988 Radiation dose and second cancer risk in patients treated for cancer of the cervix. *Radiat Res* **116**, 3-55.

45. Boice J.D. 2010 Uncertainties in studies of low statistical power. *J Radiol Prot* **30**, 115-120. (doi:10.1088/0952-4746/30/2/e02).

46. Boice J.D., Cohen S.S., Mumma M.T., Ellis E.D., Eckerman K.F., Leggett R.W., Boecker B.B., Brill A.B., Henderson B.E. 2011 Updated mortality analysis of radiation workers at Rocketdyne (Atomics International), 1948-2008. *Radiat Res* **176**, 244-258. (doi:10.1667/rr2487.1).

47. Boice J.D. 2014 The importance of radiation worker studies. *J Radiol Prot* **34**, E7-12. (doi:10.1088/ 0952-4746/34/3/e7).

48. Boice J.D. 2015 Radiation epidemiology and recent paediatric computed tomography studies. *Ann ICRP* **44**, 236-248. (doi:10.1177/0146645315575877).

49. Boice J.D., Jr. 2005 Radiation-induced thyroid cancer--what's new? *J Natl Cancer Inst* **97**, 703-705. (doi:10.1093/jnci/dji151).

50. Bollaerts K., Fierens, S., Simons, K., Francart, J., Poffijn, A., Sonck, M., Van Bladel, L., Geraets, D., Gosselin, P., Van Oyen, H., Van Eycken, L., Van Nieuwenhuyse, A. 2012 Monitoring of Possible Health Effects of Living in the Vicinity of Nuclear Sites in Belgium. Brussels, Scientific Institute of Public Health (WIV-ISP).

51. Bossew P., Tollefsen T., Cinelli G., Gruber V., De Cort M. 2015 Status of the European atlas of natural radiation. *Radiation Protection Dosimetry*. (doi:10.1093/rpd/ncv216).

52. Bouffler S.D., Meijne E.I., Huiskamp R., Cox R. 1996a Chromosomal abnormalities in neutron-induced acute myeloid leukemias in CBA/H mice. *Radiat Res* **146**, 349-352.

53. Bouffler S.D., Breckon G., Cox R. 1996b Chromosomal mechanisms in murine radiation acute myeloid leukaemogenesis. *Carcinogenesis* **17**, 655-659.

54. Bouffler S.D., Meijne E.I., Morris D.J., Papworth D. 1997 Chromosome 2 hypersensitivity and clonal development in murine radiation acute myeloid leukaemia. *Int J Radiat Biol* **72**, 181-189.

55. Bouffler S.D., Bridges B.A., Cooper D.N., Dubrova Y., McMillan T.J., Thacker J., Wright E.G., Waters R. 2006 Assessing radiation-associated mutational risk to the germline: repetitive DNA sequences as mutational targets and biomarkers. *Radiat Res* **165**, 249-268.

56. Bouffler S.D. 2016 Evidence for variation in human radiosensitivity and its potential impact on radiological protection. *Annals of the ICRP* **45 (1 Suppl)**, 280-304.

57. Brenner A.V., Tronko M.D., Hatch M., Bogdanova T.I., Oliynik V.A., Lubin J.H., Zablotska L.B., Tereschenko V.P., McConnell R.J., Zamotaeva G.A., et al. 2011 I-131 dose response for incident thyroid cancers in Ukraine related to the Chornobyl accident. *Environ Health Perspect* **119**, 933-939. (doi:10.1289/ehp.1002674).

58. Brenner D. 2014 What we know and what we don't know about cancer risks associated with radiation doses from radiological imaging. *British Journal of Radiology* **87**, 20130629.

59. Brenner D.J., Doll R., Goodhead D.T., Hall E.J., Land C.E., Little J.B., Lubin J.H., Preston D.L., Preston R.J., Puskin J.S., et al. 2003 Cancer risks attributable to low doses of ionizing radiation: assessing what we really know. *Proc Natl Acad Sci U S A* **100**, 13761-13766. (doi:10.1073/pnas.2235592100).

60. Brenner D.J. 2010 Should We be Concerned About the Rapid Increase in CT Usage? *Reviews on Environmental Health* **25**, 63-67.

61. Breslow N.E., Day, N.E. 1987 Statistical Methods in Cancer Research, Volume II - The Design and Analysis of Cohort Studies. Lyon, France, International Agency for Research on Cancer.

62. Bridges B.A., Goodhead D.T., Little M.P., Bouffler S. 2013 Evidence for Transgenerational Effects Following Exposure to Ionising Radiation. Didcot, Oxfordshire, Health Protection Agency.

63. Bromet E.J., Havenaar J.M. 2007 Psychological and perceived health effects of the Chernobyl disaster: a 20-year review. *Health Phys* **93**, 516-521. (doi:10.1097/01.hp.0000279635.14108.02).

64. Bromet E.J., Havenaar J.M., Guey L.T. 2011 A 25 Year Retrospective Review of the Psychological Consequences of the Chernobyl Accident. *Clinical Oncology* **23**, 297-305. (doi:10.1016/j.clon.2011.01.501).

65. Bromet E.J. 2012 Mental health consequences of the Chernobyl disaster. *J Radiol Prot* **32**, N71-75. (doi:10.1088/0952-4746/32/1/N71).

66. Brooks A.L., Eberlein P.E., Couch L.A., Boecker B.B. 2009 The role of dose-rate on risk from internally-deposited radionuclides and the potential need to separate dose-rate effectiveness factor (DREF) from the dose and dose-rate effectiveness factor (DDREF). *Health Physics* **97**, 458-469.

67. Brooks A.L., Hoel D.G., Preston R.J. 2016 The role of dose rate in radiation cancer risk: evaluating the effect of dose rate at the molecular, cellular and tissue levels using key events in critical

pathways following exposure to low LET radiation. *Int J Radiat Biol* **92**, 405-426. (doi:10.1080/09553002.2016.1186301).

68. Bunch K.J., Vincent T.J., Black R.J., Pearce M.S., McNally R.J.Q., McKinney P.A., Parker L., Craft A.W., Murphy M.F.G. 2014 Updated investigations of cancer excesses in individuals born or resident in the vicinity of Sellafield and Dounreay. *British Journal of Cancer* **111**, 1814-1823. (doi:10.1038/bjc.2014.357).

69. Calabrese E.J., O'Connor M.K. 2014 Estimating Risk of Low Radiation Doses - A Critical Review of the BEIR VII Report and its Use of the Linear No-Threshold (LNT) Hypothesis. *Radiat Res* **182**, 463-474. (doi:10.1667/rr13829.1).

70. Cardis E., Anspaugh L., Ivanov V., Likhtarev I., Mabuchi K., Okeanov A., Prisyazhniuk A. 1996 Estimated long term health effects of the Chernobyl accident. In *One Decade after Chernobyl*, pp. 241-279. Vienna.

71. Cardis E., Vrijheid M., Blettner M., Gilbert E., Hakama M., Hill C., Howe G., Kaldor J., Muirhead C.R., Schubauer-Berigan M., et al. 2005 Risk of cancer after low doses of ionising radiation: retrospective cohort study in 15 countries. *BMJ* **331**, 77. (doi:10.1136/bmj.38499.599861.E0).

72. Cardis E., Howe G., Ron E., Bebeshko V., Bogdanova T., Bouville A., Carr Z., Chumak V., Davis S., Demidchik Y., et al. 2006a Cancer consequences of the Chernobyl accident: 20 years on. *J Radiol Prot* **26**, 127-140. (doi:10.1088/0952-4746/26/2/001).

73. Cardis E., Krewski D., Boniol M., Drozdovitch V., Darby S.C., Gilbert E.S., Akiba S., Benichou J., Ferlay J., Gandini S., et al. 2006b Estimates of the cancer burden in Europe from radioactive fallout from the Chernobyl accident. *Int J Cancer* **119**, 1224-1235. (doi:10.1002/ijc.22037).

74. Cardis E., Vrijheid M., Blettner M., Gilbert E., Hakama M., Hill C., Howe G., Kaldor J., Muirhead C.R., Schubauer-Berigan M., et al. 2007 The 15-Country Collaborative Study of Cancer Risk among Radiation Workers in the Nuclear Industry: Estimates of Radiation-Related Cancer Risks. *Radiat Res* **167**, 396-416. (doi:10.1667/RR0553.1).

75. Cardis E., Hatch M. 2011 The Chernobyl accident--an epidemiological perspective. *Clin Oncol (R Coll Radiol)* **23**, 251-260. (doi:10.1016/j.clon.2011.01.510).

76. Cardis E., Bosch de Basea M. 2015 Comment on 'Are the studies on cancer risk from CT scans biased by indication? Elements of answer from a large-scale cohort study in France'--Evidence of confounding by predisposing factors unclear. *Br J Cancer* **113**, 1641. (doi:10.1038/bjc.2015.393).

77. CERRIE. 2004 Report of the Committee Examining Radiation Risks of Internal Emitters. London.

78. Charles M. 2010 Chernobyl: consequences of the catastrophe for people and the environment. *Radiation Protection Dosimetry* **141**, 101-104. (doi:10.1093/rpd/ncq185).

79. Chen J., Ford K.L. 2016 A study on the correlation between soil radon potential and average indoor radon potential in Canadian cities. *J Environ Radioact*. (doi:10.1016/j.jenvrad.2016.01.018).

80. Choi T.A., Costes S.V., Abergel R.J. 2015 Understanding the Health Impacts and Risks of Exposure to Radiation. In *Reflections on the Fukushima Daiichi Nuclear Accident*, pp. 259-281, Springer.

81. Clarke R., Valentin J. 2009 The history of ICRP and the evolution of its policies. *Annals of the ICRP* **39**, 75-110.

82. Coggle J.E., Williams J.P. 1990 Experimental studies of radiation carcinogenesis in the skin: a review. *Int J Radiat Biol* **57**, 797-808.

83. Coleman M.P., Estève J., Damiecki P., Arslan A., Renard H. 1993 Trends in cancer incidence and mortality. *IARC Sci Publ*, 1-806.

84. Cologne J.B., Preston D.L. 2000 Longevity of atomic-bomb survivors. *The Lancet* **356**, 303-307.

85. COMARE (Committee on Medical Aspects of Radiation in the Environment). 1999 Sixth Report: Review of radioactive particles around the Dounreay nuclear site. Health Protection Agency.

86. COMARE (Committee on Medical Aspects of Radiation in the Environment). 2002 Seventh Report: Childhood cancer following preconceptional radiation exposure. Health Protection Agency.

87. COMARE (Committee on Medical Aspects of Radiation in the Environment). 2004 Ninth report: Advice to Government on the review of the radiation risks from radioactive internal emitters carried out and published by the Committee Examining Radiation Risks of Internal Emitters. Health Protection Agency.

88. COMARE (Committee on Medical Aspects of Radiation in the Environment). 2006 Eleventh report: Childhood cancer distribution in Great Britain 1969 to 1993. Health Protection Agency.

89. COMARE (Committee on Medical Aspects of Radiation in the Environment). 2011 Fourteenth report: further consideration of the incidence of childhood leukaemia around nuclear power plants in Great Britain. Health Protection Agency

90. COMARE (Committee on Medical Aspects of Radiation in the Environment). 2016 Seventeenth report: Further consideration of the incidence of cancers around the nuclear installations at Sellafield and Dounreay.

91. Cooper J.R. 2012 Radiation protection principles. *J Radiol Prot* **32**, N81-87. (doi:10.1088/0952-4746/32/1/n81).

92. Cornforth M.N., Bailey S.M., Goodwin E.H. 2002a Dose responses for chromosome aberrations produced in noncycling primary human fibroblasts by alpha particles, and by gamma rays delivered at sublimiting low dose rates. *Radiation Research* **158**, 43-53. (doi:10.1667/0033-7587(2002)158[0043:drfcap]2.0.co;2).

93. Cornforth M.N., Bailey S.M., Goodwin E.H. 2002b Dose responses for chromosome aberrations produced in noncycling primary human fibroblasts by alpha particles, and by gamma rays delivered at sublimiting low dose rates. *Radiat Res* **158**, 43-53.

94. Cotterill S.J., Pearce M.S., Parker L. 2001 Thyroid cancer in children and young adults in the North of England. Is increasing incidence related to the Chernobyl accident? *European Journal of Cancer* **37**, 1020-1026. (doi:10.1016/s0959-8049(00)00449-4).

95. Cucinotta F.A., Nikjoo H., O'Neill P., Goodhead D.T. 2000 Kinetics of DSB rejoining and formation of simple chromosome exchange aberrations. *Int J Radiat Biol* **76**, 1463-1474.

96. Dadson S.J., Hall J.W., Murgatroyd A., Acreman M., Bates P., Beven K., Heathwaite L., Holden J., Holman I.P., Lane S.N., et al. 2017 A restatement of the natural science evidence concerning catchment-based 'natural' flood management in the UK. *Proc Math Phys Eng Sci* **473**, 20160706. (doi:10.1098/rspa.2016.0706).

97. Darby S., Hill D., Auvinen A., Barros-Dios J.M., Baysson H., Bochicchio F., Deo H., Falk R., Forastiere F., Hakama M., et al. 2005 Radon in homes and risk of lung cancer: collaborative analysis of individual data from 13 European case-control studies. *BMJ* **330**, 223. (doi:10.1136/bmj.38308.477650.63).

98. Darby S., Hill D., Deo H., Auvinen A., Barros-Dios J.M., Baysson H., Bochicchio F., Falk R., Farchi S., Figueiras A. 2006 Residential radon and lung cancer—detailed results of a collaborative analysis of individual data on 7148 persons with lung cancer and 14 208 persons without lung cancer from 13 epidemiologic studies in Europe. *Scand J Work Environ Health*, 1-84.

99. Darby S.C., Olsen J.H., Doll R., Thakrar B., Brown P.D., Storm H.H., Barlow L., Langmark F., Teppo L., Tulinius H. 1992 Trends in childhood leukaemia in the Nordic countries in relation to fallout from atmospheric nuclear weapons testing. *Br Med J* **304**, 1005-1009.

100. Darby S.C., Whitley E., Howe G.R., Hutchings S.J., Kusiak R.A., Lubin J.H., Morrison H.I., Tirmarche M., Tomasek L., Radford E.P., et al.

1995 Radon and cancers other than lung cancer in underground miners: a collaborative analysis of 11 studies. *J Natl Cancer Inst* **87**, 378-384.

101. Darby S.C., Ewertz M., McGale P., Bennet A.M., Blom-Goldman U., Bronnum D., Correa C., Cutter D., Gagliardi G., Gigante B., et al. 2013 Risk of ischemic heart disease in women after radiotherapy for breast cancer. *N Engl J Med* **368**, 987-998. (doi:10.1056/NEJMoa1209825).

102. Dardynskaia I., Imrey P.B., Okeanov A., Hryhorczuk D. 2006 Breast cancer trends in two oblasts of Belarus and the Chernobyl accident. *Int J Occup Environ Health* **12**, 415-422. (doi:10.1179/oeh.2006.12.4.415).

103. Dauer L.T., Brooks A.L., Hoel D.G., Morgan W.F., Stram D., Tran P. 2010 Review and evaluation of updated research on the health effects associated with low-dose ionising radiation. *Radiation Protection Dosimetry*, 103-136.

104. Davis F.G., Yu K.L., Preston D., Epifanova S., Degteva M., Akleyev A.V. 2015 Solid Cancer Incidence in the Techa River Incidence Cohort: 1956-2007. *Radiat Res* **184**, 56-65. (doi:10.1667/rr14023.1).

105. Davis S., Kopecky K.J., Hamilton T.E., Onstad L. 2004 Thyroid neoplasia, autoimmune thyroiditis, and hypothyroidism in persons exposed to iodine 131 from the Hanford nuclear site. *JAMA* **292**, 2600-2613. (doi:10.1001/jama.292.21.2600).

106. Davis S., Day R., Kopecky K., Mahoney M., McCarthy P., Michalek A., Moysich K., Onstad L., Stepanenko V., Voillequé P. 2006 Childhood leukaemia in Belarus, Russia, and Ukraine following the Chernobyl power station accident: results from an international collaborative population-based case–control study. *Int J Epidemiol* **35**, 386-396.

107. Deckbar D., Birraux J., Krempler A., Tchouandong L., Beucher A., Walker S., Stiff T., Jeggo P., Lobrich M. 2007 Chromosome breakage after G2 checkpoint release. *J Cell Biol* **176**, 749-755. (doi:10.1083/jcb.200612047).

108. Delongchamp R.R., Mabuchi K., Yoshimoto Y., Preston D.L. 1997 Cancer mortality among atomic bomb survivors exposed in utero or as young children, October 1950-May 1992. *Radiat Res* **147**, 385-395.

109. Demoury C., Marquant F., Ielsch G., Goujon S., Debayle C., Faure L., Coste A., Laurent O., Guillevic J., Laurier D., et al. 2016 Residential exposure to natural background radiation and risk of childhood acute leukemia in France, 1990 - 2009. *Environ Health Perspect*. (doi:10.1289/EHP296).

110. Ding L.H., Shingyoji M., Chen F., Hwang J.J., Burma S., Lee C., Cheng J.F., Chen D.J. 2005 Gene expression profiles of normal human fibroblasts after exposure to ionizing radiation: a comparative study of low and high doses. *Radiat Res* **164**, 17-26.

111. Doll R., Evans H.J., Darby S.C. 1994 Paternal exposure not to blame. *Nature* **367**, 678-680. (doi:10.1038/367678a0).

112. Doll R., Wakeford R. 1997 Risk of childhood cancer from fetal irradiation. *Br J Radiol* **70**, 130-139. (doi:10.1259/bjr.70.830.9135438).

113. Doll R. 2002 Proof of causality: deduction from epidemiological observation. *Perspect Biol Med* **45**, 499-515.

114. Dom G., Tarabichi M., Unger K., Thomas G., Oczko-Wojciechowska M., Bogdanova T., Jarzab B., Dumont J.E., Detours V., Maenhaut C. 2012 A gene expression signature distinguishes normal tissues of sporadic and radiation-induced papillary thyroid carcinomas. *Br J Cancer* **107**, 994-1000. (doi:10.1038/bjc.2012.302).

115. Donnelly E.H., Nemhauser J.B., Smith J.M., Kazzi Z.N., Farfan E.B., Chang A.S., Naeem S.F. 2010 Acute radiation syndrome: assessment and management. *South Med J* **103**, 541-546. (doi:10.1097/SMJ.0b013e3181ddd571).

116. dos Santos Silva I., De Stavola B., Pizzi C., Evans A.D., Evans S.A. 2013 Cancer incidence in professional flight crew and air traffic control officers: disentangling the effect of occupational versus lifestyle exposures. *Int J Cancer* **132**, 374-384. (doi:10.1002/ijc.27612).

117. Doss M. 2013 Linear No-Threshold Model VS. Radiation Hormesis. *Dose Response* **11**, 480-497. (doi:10.2203/dose-response.13-005.Doss).

118. Doss M. 2015 INWORKS study: risk of leukaemia from protracted radiation exposure. *Lancet Haematol* **2**, e404-405. (doi:10.1016/S2352-3026(15)00145-3).

119. Doyi I., Essumang D.K., Dampare S., Glover E.T. 2016 Technologically Enhanced Naturally Occurring Radioactive Materials (TENORM) in the Oil and Gas Industry: A Review. *Rev Environ Contam Toxicol* **238**, 107-119. (doi:10.1007/398_2015_5005).

120. Dubrova Y.E., Nesterov V.N., Krouchinsky N.G., Ostapenko V.A., Neumann R., Neil D.L., Jeffreys A.J. 1996 Human minisatellite mutation rate after the Chernobyl accident. *Nature* **380**, 683-686. (doi:10.1038/380683a0).

121. Dubrova Y.E., Nesterov V.N., Krouchinsky N.G., Ostapenko V.A., Vergnaud G., Giraudeau F., Buard J., Jeffreys A.J. 1997 Further evidence for elevated human minisatellite mutation rate in Belarus eight years after the Chernobyl accident. *Mutat Res* **381**, 267-278.

122. Dubrova Y.E., Grant G., Chumak A.A., Stezhka V.A., Karakasian A.N. 2002 Elevated minisatellite mutation rate in the post-Chernobyl families from Ukraine. *Am J Hum Genet* **71**, 801-809. (doi:10.1086/342729).

123. Easton D.F. 1994 Cancer risks in A-T heterozygotes. *Int J Radiat Biol* **66**, S177-S182.

124. Edwards A.A., Lloyd D.C. 1998 Risks from ionising radiation: deterministic effects. *J Radiol Prot* **18**, 175-183. (doi:10.1088/0952-4746/18/3/004).

125. Euratom. 2013 Council Directive 2013/59 EURATOM of 5 December 2013 Laying Down the Basic Safety Standards for the Protection of the Health of Workers and the General Public against the Dangers Arising from Ionizing Radiation, and Repealing Directives 89/618/Euratom. Brussels.

126. Everitt B., Skrondal A. 2010 *The Cambridge Dictionary of Statistics*. Cambridge, Cambridge University Press.

127. Fairlie I. 2014 A hypothesis to explain childhood cancers near nuclear power plants. *Journal of Environmental Radioactivity* **133**, 10-17. (doi:10.1016/j.jenvrad.2013.07.024).

128. Feinendegen L.E. 2005 Evidence for beneficial low level radiation effects and radiation hormesis. *Br J Radiol* **78**, 3-7.

129. Fernet M., Megnin-Chanet F., Hall J., Favaudon V. 2010 Control of the G2/M checkpoints after exposure to low doses of ionising radiation: implications for hyper-radiosensitivity. *DNA Repair (Amst)* **9**, 48-57. (doi:10.1016/j.dnarep.2009.10.006).

130. Flockerzi E., Schanz S., Rube C.E. 2014 Even low doses of radiation lead to DNA damage accumulation in lung tissue according to the genetically-defined DNA repair capacity. *Radiother Oncol* **111**, 212-218. (doi:10.1016/j.radonc.2014.03.011).

131. Francis S.S., Selvin S., Yang W., Buffler P.A., Wiemels J.L. 2012 Unusual space-time patterning of the Fallon, Nevada leukemia cluster: Evidence of an infectious etiology. *Chemico-Biological Interactions* **196**, 102-109. (doi:10.1016/j.cbi.2011.02.019).

132. Fritz T.E. 2002 The influence of dose, dose-rate and radiation quality on the effect of protracted whole body irradiation of beagles. *Brit J Radiol suppl* **26**, 103-111.

133. Fry S.A. 1998 Studies of U.S. radium dial workers: an epidemiological classic. *Radiat Res* **150**, S21-29.

134. Fujiwara S., Suyama A., Cologne J.B., Akahoshi M., Yamada M., Suzuki G., Koyama K., Takahashi N., Kasagi E., Grant E.J., et al. 2008 Prevalence of adult-onset multifactorial disease among offspring of atomic bomb survivors. *Radiat Res* **170**, 451-457.

135. Furitsu K., Ryo H., Yeliseeva K.G., Thuy le T.T., Kawabata H., Krupnova E.V., Trusova V.D., Rzheutsky V.A., Nakajima H., Kartel N., et al. 2005 Microsatellite mutations show no increases in the children of the Chernobyl liquidators. *Mutat Res* **581**, 69-82. (doi:10.1016/j.mrgentox.2004.11.002).

136. Furukawa K., Preston D.L., Lonn S., Funamoto S., Yonehara S., Matsuo T., Egawa H., Tokuoka S., Ozasa K., Kasagi F., et al. 2010 Radiation and smoking effects on lung cancer incidence among atomic bomb survivors. *Radiat Res* **174**, 72-82. (doi:10.1667/RR2083.1).

137. Gardner M.J., Snee M.P., Hall A.J., Powell C.A., Downes S., Terrell J.D. 1990 Results of case-control study of leukemia and lymphoma among young people near Sellafield nuclear plant in West Cumbria. *Br Med J* **300**, 423-429.

138. Gilbert E.S., Sokolnikov M.E., Preston D.L., Schonfeld S.J., Schadilov A.E., Vasilenko E.K., Koshurnikova N.A. 2013 Lung cancer risks from plutonium: an updated analysis of data from the Mayak worker cohort. *Radiat Res* **179**, 332-342. (doi:10.1667/rr3054.1).

139. Gillies M., Haylock R. 2014 The cancer mortality and incidence experience of workers at British Nuclear Fuels plc, 1946-2005. *J Radiol Prot* **34**, 595-623. (doi:10.1088/0952-4746/34/3/595).

140. Godfray H.C., Blacquiere T., Field L.M., Hails R.S., Potts S.G., Raine N.E., Vanbergen A.J., McLean A.R. 2015 A restatement of recent advances in the natural science evidence base concerning neonicotinoid insecticides and insect pollinators. *Proc Biol Sci* **282**, 20151821. (doi:10.1098/rspb.2015.1821).

141. Godfray H.C.J., Donnelly C.A., Kao R.R., Macdonald W., McDonald R.A., Petrokofsky G., Wood J.L.N., Woodroffe R., Young D.B., McLean A.R. 2013 A restatement of the natural science evidence base relevant to the control of bovine tuberculosis in Great Britain. *Proceedings of the Royal Society B-Biological Sciences* **280**, 9. (doi:10.1098/rspb.2013.1634).

142. Godfray H.C.J., Blacquiere T., Field L.M., Hails R.S., Petrokofsky G., Potts S.G., Raine N.E., Vanbergen A.J., McLean A.R. 2014 A restatement of the natural science evidence base concerning neonicotinoid insecticides and insect pollinators. *Proceedings of the Royal Society B-Biological Sciences* **281**, 9. (doi:10.1098/rspb.2014.0558).

143. Gonzalez A.J., Akashi M., Boice J.D., Jr., Chino M., Homma T., Ishigure N., Kai M., Kusumi S., Lee J.K., Menzel H.G., et al. 2013 Radiological protection issues arising during and after the Fukushima nuclear reactor accident. *J Radiol Prot* **33**, 497-571. (doi:10.1088/0952-4746/33/3/497).

144. Goodhead D.T. 1994 Initial Events in the Cellular Effects of Ionizing Radiations: Clustered Damage in DNA. *Int J Radiat Biol* **65**, 7-17. (doi:doi:10.1080/09553009414550021).

145. Goodhead D.T. 2009 Fifth Warren K. Sinclair Keynote Address: Issues in quantifying the effects of low-level radiation. *Health Phys* **97**, 394-406. (doi:10.1097/HP.0b013e3181ae8acf).

146. Grant E.J., Furukawa K., Sakata R., Sugiyama H., Sadakane A., Takahashi I., Utada M., Shimizu Y., Ozasa K. 2015 Risk of death among children of atomic bomb survivors after 62 years of follow-up: a cohort study. *Lancet Oncol* **16**, 1316-1323. (doi:10.1016/S1470-2045(15)00209-0).

147. Grant E.J., Brenner A., Sugiyama H., Sakata R., Sadakane A., Utada M., Cahoon E.K., Milder C.M., Soda M., Cullings H.M., et al. 2017 Solid cancer incidence among the Life Span Study of atomic bomb survivors: 1958-2009. *Radiat Res*. (doi:10.1667/rr14492.1).

148. Gray A., Read S., McGale P., Darby S. 2009 Lung cancer deaths from indoor radon and the cost effectiveness and potential of policies to reduce them. *BMJ* **338**, a3110. (doi:10.1136/bmj.a3110).

149. Gurtler A., Hauptmann M., Pautz S., Kulka U., Friedl A.A., Lehr S., Hornhardt S., Gomolka M. 2014 The inter-individual variability outperforms the intra-individual variability of differentially expressed proteins prior and post irradiation in lymphoblastoid cell lines. *Arch Physiol Biochem* **120**, 198-207. (doi:10.3109/13813455.2014.953548).

150. Haley B.M., Paunesku T., Grdina D.J., Woloschak G.E. 2015 The Increase in Animal Mortality Risk following Exposure to Sparsely Ionizing Radiation Is Not Linear Quadratic with Dose. *PLoS One* **10**, e0140989. (doi:10.1371/journal.pone.0140989).

151. Hall E.J., Giaccia A.J. 2011 Radiobiology for the Radiologist. 7th ed. Philadelphia, Lippincott Williams & Wilkins.

152. Hall J., Jeggo P.A., West C., Gomolka M., Quintens R., Badie C., Laurent O., Aerts A., Anastasov N., Azimzadeh O., et al. 2017 Ionizing radiation biomarkers in epidemiological studies - An update. *Mutat Res* **771**, 59-84. (doi:10.1016/j.mrrev.2017.01.001).

153. Hamada N. 2016 Ionizing radiation sensitivity of the ocular lens and its dose rate dependence. *Int J Radiat Biol*, December (online) 1-11. (doi:10.1080/09553002.2016.1266407).

154. Hamblin T.J. 2008 Have we been wrong about ionizing radiation and chronic lymphocytic leukemia? *Leuk Res* **32**, 523-525. (doi:10.1016/j.leukres.2007.08.015).

155. Hammer G.P., Auvinen A., De Stavola B.L., Grajewski B., Gundestrup M., Haldorsen T., Hammar N., Lagorio S., Linnersjo A., Pinkerton L., et al. 2014 Mortality from cancer and other causes in commercial airline crews: a joint analysis of cohorts from 10 countries. *Occup Environ Med* **71**, 313-322. (doi:10.1136/oemed-2013-101395).

156. Hansson G.K., Hermansson A. 2011 The immune system in atherosclerosis. *Nat Immunol* **12**, 204-212. (doi:10.1038/ni.2001).

157. Harrison J., Day P. 2008 Radiation doses and risks from internal emitters. *J Radiol Prot* **28**, 137-159. (doi:10.1088/0952-4746/28/2/r01).

158. Harrison J.D., Marsh J.W. 2012 Effective dose from inhaled radon and its progeny. *Ann ICRP* **41**, 378-388. (doi:10.1016/j.icrp.2012.06.012).

159. Hasegawa A., Tanigawa K., Ohtsuru A., Yabe H., Maeda M., Shigemura J., Ohira T., Tominaga T., Akashi M., Hirohashi N. 2015 Health effects of radiation and other health problems in the aftermath of nuclear accidents, with an emphasis on Fukushima. *The Lancet* **386**, 479-488.

160. Hasegawa A., Ohira T., Maeda M., Yasumura S., Tanigawa K. 2016 Emergency Responses and Health Consequences after the Fukushima Accident; Evacuation and Relocation. *Clin Oncol (R Coll Radiol)* **28**, 237-244. (doi:10.1016/j.clon.2016.01.002).

161. Hatch M., Ostroumova E., Brenner A., Federenko Z., Gorokh Y., Zvinchuk O., Shpak V., Tereschenko V., Tronko M., Mabuchi K. 2015 Non-thyroid cancer in Northern Ukraine in the post-Chernobyl period: Short report. *Cancer Epidemiol* **39**, 279-283. (doi:10.1016/j.canep.2015.02.002).

162. Hauptmann M., Mohan A.K., Doody M.M., Linet M.S., Mabuchi K. 2003 Mortality from diseases of the circulatory system in radiologic technologists in the United States. *Am J Epidemiol* **157**, 239-248.

163. Hauptmann M., Haghdoost S., Gomolka M., Sarioglu H., Ueffing M., Dietz A., Kulka U., Unger K., Babini G., Harms-Ringdahl M., et al. 2016 Differential Response and Priming Dose Effect on the Proteome of Human Fibroblast and Stem Cells Induced by

Exposure to Low Doses of Ionizing Radiation. *Radiat Res* **185**, 299-312. (doi:10.1667/rr14226.1).

164. Havenaar J.M., Van den Brink W., Van den Bout J., Kasyanenko A.P., Poelijoe N.W., Wholfarth T., Meijler-Iljina L.I. 1996 Mental health problems in the Gomel region (Belarus): an analysis of risk factors in an area affected by the Chernobyl disaster. *Psychol Med* **26**, 845-855.

165. Havenaar J.M., Rumyantzeva G.M., van den Brink W., Poelijoe N.W., van den Bout J., van Engeland H., Koeter M.W. 1997 Long-term mental health effects of the Chernobyl disaster: an epidemiologic survey in two former Soviet regions. *Am J Psychiatry* **154**, 1605-1607.

166. Hayashida N., Imaizumi M., Shimura H., Okubo N., Asari Y., Nigawara T., Midorikawa S., Kotani K., Nakaji S., Otsuru A., et al. 2013 Thyroid ultrasound findings in children from three Japanese prefectures: Aomori, Yamanashi and Nagasaki. *PLoS One* **8**, e83220. (doi:10.1371/journal.pone.0083220).

167. Hei T.K., Zhou H., Chai Y., Ponnaiya B., Ivanov V.N. 2011 Radiation induced non-targeted response: mechanism and potential clinical implications. *Curr Mol Pharmacol* **4**, 96-105.

168. Heinavaara S., Toikkanen S., Pasanen K., Verkasalo P.K., Kurttio P., Auvinen A. 2010 Cancer incidence in the vicinity of Finnish nuclear power plants: an emphasis on childhood leukemia. *Cancer Causes & Control* **21**, 587-595. (doi:10.1007/s10552-009-9488-7).

169. Hendry J.H., Simon S.L., Wojcik A., Sohrabi M., Burkart W., Cardis E., Laurier D., Tirmarche M., Hayata I. 2009 Human exposure to high natural background radiation: what can it teach us about radiation risks? *J Radiol Prot* **29**, A29.

170. Hendry J.H., Niwa O., Barcellos-Hoff M.H., Globus R.K., Harrison J., Martin M.T., Seed T.M., Shay J.W., Story M.D., Suzuki K., et al. 2016 Stem cell biology with respect to carcinogenesis aspects of radiological protection. *Annals of the ICRP* **45 (1 Suppl)**, 239-279.

171. Hernán M.A. 2010 The Hazards of Hazard Ratios. *Epidemiology* **21**, 13-15. (doi:10.1097/EDE.0b013e3181c1ea43).

172. Hill A.B. 2015 The environment and disease: association or causation? 1965. *J R Soc Med* **108**, 32-37. (doi:10.1177/0141076814562718).

173. Hlatky L., Sachs R.K., Vazquez M., Cornforth M.N. 2002 Radiation-induced chromosome aberrations: insights gained from biophysical modeling. *Bioessays* **24**, 714-723. (doi:10.1002/bies.10126).

174. Hoffmann M., Aliyev C.S., Feyzullayev A.A., Baghirli R.J., Veliyeva F.F., Pampuri L., Valsangiacomo C., Tollefsen T., Cinelli G. 2016 First Map of Residential Indoor Radon Measurements in Azerbaijan. *Radiat Prot Dosimetry*. (doi:10.1093/rpd/ncw284).

175. Holt E. 2010 Debate over health effects of Chernobyl re-ignited. *The Lancet* **375**, 1424-1425.

176. Howe G.R. 1995 Lung cancer mortality between 1950 and 1987 after exposure to fractionated moderate-dose-rate ionizing radiation in the Canadian fluoroscopy cohort study and a comparison with lung cancer mortality in the Atomic Bomb survivors study. *Radiat Res* **142**, 295-304.

177. Howe G.R., Zablotska L.B., Fix J.J., Egel J., Buchanan J. 2004 Analysis of the mortality experience amongst U.S. nuclear power industry workers after chronic low-dose exposure to ionizing radiation. *Radiat Res* **162**, 517-526.

178. Hsu W.L., Preston D.L., Soda M., Sugiyama H., Funamoto S., Kodama K., Kimura A., Kamada N., Dohy H., Tomonaga M., et al. 2013 The incidence of leukemia, lymphoma and multiple myeloma among atomic bomb survivors: 1950-2001. *Radiat Res* **179**, 361-382. (doi:10.1667/RR2892.1).

179. Hughes J.S., Watson S.J., Jones A.L., Oatway W.B. 2005 Review of the radiation exposure of the UK population. *J Radiol Prot* **25**, 493-496. (doi:10.1088/0952-4746/25/4/010).

180. IAEA. 2002 Safety Report 21: Optimization of Radiation Exposure in the Control of Occupational Exposure. Vienna.

181. IAEA (International Atomic Energy Authority). 2003 Safety Report 34: Radiation and Waste Safety in the Oil and Gas Industry. Vienna.

182. IAEA (International Atomic Energy Authority). 2014 Radiation Protection and Safety of Radiation Sources: International Basic Safety Standards. Vienna.

183. IAEA (International Atomic Energy Authority). 2015 Radiological Consequences of the Fukushima Daiichi Accident. Vienna.

184. Iannuzzi C.M., Atencio D.P., Green S., Stock R.G., Rosenstein B.S. 2002 ATM mutations in female breast cancer patients predict for an increase in radiation-induced late effects. *Int J Radiat Oncol Biol Phys* **52**, 606-613.

185. ICRP 99. 2005 Low-dose Extrapolation of Radiation-related Cancer Risk. *Annals of the ICRP*, 4.

186. ICRP 103. 2007 The 2007 Recommendations of the International Commission on Radiological Protection. *Annals of the ICRP* **37**, 1-332.

187. ICRP. 2003 Relative biological effectiveness (RBE), quality factor (Q), and radiation weighting factor (w(R)). A report of the International Commission on Radiological Protection. *Annals of the ICRP* **33**, 1-117.

188. ICRU (International Commission on Radiation Units and Measurements). 2011 Fundamental Quantities and Units for Ionizing Radiation (Revised).

189. Inkret W.C., Meinhold, C.B., Taschner, J.C. 1995 Radiation and Risk: a hard look at the data - protection standards. *Los Alamos Science* **23**, 117-124.

190. Ivanov V., Ilyin L., Gorski A., Tukov A., Naumenko R. 2004 Radiation and epidemiological analysis for solid cancer incidence among nuclear workers who participated in recovery operations following the accident at the Chernobyl NPP. *J Radiat Res* **45**, 41-44.

191. Ivanov V.K., Maksioutov M.A., Chekin S.Y., Petrov A.V., Biryukov A.P., Kruglova Z.G., Matyash V.A., Tsyb A.F., Manton K.G., Kravchenko J.S. 2006 The risk of radiation-induced cerebrovascular disease in Chernobyl emergency workers. *Health Phys* **90**, 199-207. (doi:10.1097/01.HP.0000175835.31663.ea).

192. Ivanov V.K., Chekin S.Y., Kashcheev V.V., Maksioutov M.A., Tumanov K.A. 2008 Risk of thyroid cancer among Chernobyl emergency workers of Russia. *Radiat Environ Biophys* **47**, 463-467. (doi:10.1007/s00411-008-0177-9).

193. Ivanov V.K., Kashcheev V.V., Chekin S.Y., Maksioutov M.A., Tumanov K.A., Vlasov O.K., Shchukina N.V., Tsyb A.F. 2012 Radiation-epidemiological studies of thyroid cancer incidence in Russia after the Chernobyl accident (estimation of radiation risks, 1991-2008 follow-up period). *Radiation Protection Dosimetry* **151**, 489-499. (doi:10.1093/rpd/ncs019).

194. Izumi S., Suyama A., Koyama K. 2003 Radiation-related mortality among offspring of atomic bomb survivors: a half-century of follow-up. *Int J Cancer* **107**, 292-297. (doi:10.1002/ijc.11400).

195. Jablon S., Kato H. 1970 Childhood cancer in relation to prenatal exposure to atomic-bomb radiation. *Lancet* **2**, 1000-1003.

196. Jablon S., Miller R.W. 1978 Army technologists - 29-year follow up for cause of death. *Radiology* **126**, 677-679.

197. Jacob S., Michel M., Brezin A.P., Laurier D., Bernier M.-O. 2012 Ionizing radiation as a risk factor for cataract: what about low-dose effects? *J Clin Experiment Ophthalmol* **S1**. (doi:10.4172/2155-9570.S1-005).

198. Janiak M.K. 2014 Epidemiological evidence of childhood leukaemia around nuclear power plants. *Dose-Response* **12**, 349-364. (doi:10.2203/dose-response.14-005.Janiak).

199. Jargin S.V. 2010 Overestimation of Chernobyl consequences: poorly substantiated information published. *Radiat Environ Biophys* **49**, 743-745; author reply 747-748. (doi:10.1007/s00411-010-0313-1).

200. Jorgensen T.J. 2016 Re: Thyroid Cancer Among Young People in Fukushima. *Epidemiology* **27**, e17. (doi:10.1097/EDE.0000000000000465).

201. Journy N., Rehel J., Le Pointe H.D., Lee C., Brisse H., Chateil J., Caer-Lorho S., Laurier D., Bernier M. 2015 Are the studies on cancer risk from CT scans biased by indication? Elements of answer from a large-scale cohort study in France. *British Journal of Cancer* **112**, 185-193.

202. Kaatsch P., Spix C., Jung I., Blettner M. 2008a Childhood leukemia in the vicinity of nuclear power plants in Germany. *Dtsch Arztebl Int* **105**, 725-732. (doi:10.3238/arztebl.2008.0725).

203. Kaatsch P., Spix C., Schulze-Rath R., Schmiedel S., Blettner M. 2008b Leukaemia in young children living in the vicinity of German nuclear power plants. *International Journal of Cancer* **122**, 721-726. (doi:10.1002/ijc.23330).

204. Kashcheev V.V., Chekin S.Y., Maksioutov M.A., Tumanov K.A., Kochergina E.V., Kashcheeva P.V., Shchukina N.V., Ivanov V.K. 2015 Incidence and mortality of solid cancer among emergency workers of the Chernobyl accident: assessment of radiation risks for the follow-up period of 1992-2009. *Radiat Environ Biophys* **54**, 13-23. (doi:10.1007/s00411-014-0572-3).

205. Kashcheev V.V., Chekin S.Y., Maksioutov M.A., Tumanov K.A., Menyaylo A.N., Kochergina E.V., Kashcheeva P.V., Gorsky A.I., Shchukina N.V., Karpenko S.V., et al. 2016 Radiation-epidemiological Study of Cerebrovascular Diseases in the Cohort of Russian Recovery Operation Workers of the Chernobyl Accident. *Health Phys* **111**, 192-197. (doi:10.1097/HP.0000000000000523).

206. Kendall G.M., Little M.P., Wakeford R., Bunch K.J., Miles J.C., Vincent T.J., Meara J.R., Murphy M.F. 2013 A record-based case-control study of natural background radiation and the incidence of childhood leukaemia and other cancers in Great Britain during 1980-2006. *Leukemia* **27**, 3-9. (doi:10.1038/leu.2012.151).

207. Kesminiene A., Evrard A.S., Ivanov V.K., Malakhova I.V., Kurtinaitis J., Stengrevics A., Tekkel M., Anspaugh L.R., Bouville A., Chekin S., et al. 2008 Risk of hematological malignancies among Chernobyl liquidators. *Radiat Res* **170**, 721-735. (doi:10.1667/RR1231.1).

208. Kinlen L. 1988 Evidence for an infective cause of childhood leukemia - comparison of a Scottish new town with nuclear reprocessing sites in Britain. *Lancet* **2**, 1323-1327.

209. Kinlen L. 2011 Childhood leukaemia, nuclear sites, and population mixing. *British Journal of Cancer* **104**, 12-18. (doi:10.1038/sj.bjc.6605982).

210. Kinlen L. 2015 Population mixing and childhood leukaemia. *Eur J Epidemiol* **30**, 1331. (doi:10.1007/s10654-015-0107-5).

211. Kinlen L.J. 2012 An examination, with a meta-analysis, of studies of childhood leukaemia in relation to population mixing. *British Journal of Cancer* **107**, 1163-1168. (doi:10.1038/bjc.2012.402).

212. Kinley D. 2006 Chernobyl's Legacy: Health, Environmental and Socio-Economic Impacts. Belarus, The Russian Federation, Ukraine, The Chernobyl Forum/IAEA.

213. Kitahara C., Linet M., Rajaraman P., Ntowe E., Berrington de González A. 2015 A New Era of Low-Dose Radiation Epidemiology. *Current Environmental Health Reports* **2**, 236-249. (doi:10.1007/s40572-015-0055-y).

214. Kiuru A., Auvinen A., Luokkamaki M., Makkonen K., Veidebaum T., Tekkel M., Rahu M., Hakulinen T., Servomaa K., Rytomaa T., et al. 2003 Hereditary minisatellite mutations among the offspring of Estonian Chernobyl cleanup workers. *Radiat Res* **159**, 651-655.

215. Kodaira M., Izumi S., Takahashi N., Nakamura N. 2004 No evidence of radiation effect on mutation rates at hypervariable minisatellite loci in the germ cells of atomic bomb survivors. *Radiat Res* **162**, 350-356.

216. Kodaira M., Ryo H., Kamada N., Furukawa K., Takahashi N., Nakajima H., Nomura T., Nakamura N. 2010 No evidence of increased mutation rates at microsatellite loci in offspring of A-bomb survivors. *Radiat Res* **173**, 205-213. (doi:10.1667/RR1991.1).

217. Korblein A. 2016 Re: Thyroid Cancer Among Young People in Fukushima. *Epidemiology* **27**, e18-19. (doi:10.1097/EDE.0000000000000463).

218. Krestinina L.Y., Epifanova S., Silkin S., Mikryukova L., Degteva M., Shagina N., Akleyev A. 2013a Chronic low-dose exposure in the Techa River Cohort: risk of mortality from circulatory diseases. *Radiat Environ Biophys* **52**, 47-57. (doi:10.1007/s00411-012-0438-5).

219. Krestinina L.Y., Davis F.G., Schonfeld S., Preston D.L., Degteva M., Epifanova S., Akleyev A.V. 2013b Leukaemia incidence in the Techa River Cohort: 1953-2007. *Br J Cancer* **109**, 2886-2893. (doi:10.1038/bjc.2013.614).

220. Kreuzer M., Fenske N., Schnelzer M., Walsh L. 2015a Lung cancer risk at low radon exposure rates in German uranium miners. *Br J Cancer* **113**, 1367-1369. (doi:10.1038/bjc.2015.324).

221. Kreuzer M., Dufey F., Laurier D., Nowak D., Marsh J.W., Schnelzer M., Sogl M., Walsh L. 2015b Mortality from internal and external radiation exposure in a cohort of male German uranium millers, 1946-2008. *Int Arch Occup Environ Health* **88**, 431-441. (doi:10.1007/s00420-014-0973-2).

222. Kreuzer M., Sobotzki C., Fenske N., Marsh J.W., Schnelzer M. 2016 Leukaemia mortality and low-dose ionising radiation in the WISMUT uranium miner cohort (1946-2013). *Occup Environ Med*. (doi:10.1136/oemed-2016-103795).

223. Krewski D., Lubin J.H., Zielinski J.M., Alavanja M., Catalan V.S., William Field R., Klotz J.B., Létourneau E.G., Lynch C.F., Lyon J.L. 2006 A combined analysis of North American case-control studies of residential radon and lung cancer. *Journal of Toxicology and Environmental Health, Part A* **69**, 533-597.

224. Kuznetsova I.S., Labutina E.V., Hunter N. 2016 Radiation Risks of Leukemia, Lymphoma and Multiple Myeloma Incidence in the Mayak Cohort: 1948–2004. *PLoS One* **11**, e0162710. (doi:10.1371/journal.pone.0162710).

225. Land C.E. 1980 Estimating cancer risks from low doses of ionizing radiation. *Science* **209**, 1197-1203.

226. Land C.E., Bouville A., Apostoaei I., Simon S.L. 2010 Projected lifetime cancer risks from exposure to regional radioactive fallout in the Marshall Islands. *Health Phys* **99**, 201-215. (doi:10.1097/HP.0b013e3181dc4e84).

227. Lane R.D., E; Burtt, J; Thompson, P A. 2013 Radiation Exposure and Cancer Incidence (1990 to 2008) around Nuclear Power Plants in Ontario, Canada. *Journal of Environmental Protection* **4**, 888-913. (doi:10.4236/jep.2013.49104).

228. Lane R.S., Frost S.E., Howe G.R., Zablotska L.B. 2010 Mortality (1950-1999) and cancer incidence (1969-1999) in the cohort of Eldorado uranium workers. *Radiat Res* **174**, 773-785. (doi:10.1667/rr2237.1).

229. Laurier D., Jacob S., Bernier M.O., Leuraud K., Metz C., Samson E., Laloi P. 2008 Epidemiological studies of leukaemia in children and young adults around nuclear facilities: a critical review. *Radiat Prot Dosimetry* **132**, 182-190. (doi:10.1093/rpd/ncn262).

230. Laurier D., Grosche B., Auvinen A., Clavel J., Cobaleda C., Dehos A., Hornhardt S., Jacob S., Kaatsch P., Kosti O., et al. 2014 Childhood leukaemia risks: from unexplained findings near nuclear installations to recommendations for future research. *J Radiol Prot* **34**, R53-R68. (doi:10.1088/0952-4746/34/3/r53).

231. Lecomte J.F., Solomon S., Takala J., Jung T., Strand P., Murith C., Kiselev S., Zhuo W., Shannoun F., Janssens A. 2014 ICRP Publication 126: Radiological Protection against Radon Exposure. *Ann ICRP* **43**, 5-73. (doi:10.1177/0146645314542212).

232. Leszczynski D. 2014 Radiation proteomics: a brief overview. *Proteomics* **14**, 481-488. (doi:10.1002/pmic.201300390).

233. Leuraud K., Richardson D.B., Cardis E., Daniels R.D., Gillies M., O'Hagan J.A., Hamra G.B., Haylock R., Laurier D., Moissonnier M. 2015 Ionising radiation and risk of death from leukaemia and lymphoma in radiation-monitored workers (INWORKS): an international cohort study. *The Lancet Haematology* **2**, e276-e281.

234. Lim S.S., Vos T., Flaxman A.D., Danaei G., Shibuya K., Adair-Rohani H., AlMazroa M.A., Amann M., Anderson H.R., Andrews K.G. 2013 A comparative risk assessment of burden of disease and injury attributable to 67 risk factors and risk factor clusters in 21 regions, 1990–2010: a systematic analysis for the Global Burden of Disease Study 2010. *The Lancet* **380**, 2224-2260.

235. Linet M.S., Slovis T.L., Miller D.L., Kleinerman R., Lee C., Rajaraman P., Berrington de Gonzalez A. 2012 Cancer risks associated with external radiation from diagnostic imaging procedures. *CA Cancer J Clin* **62**, 75-100. (doi:10.3322/caac.21132).

236. Little J., McLaughlin J., Miller A. 2008a Leukaemia in young children living in the vicinity of nuclear power plants. *International Journal of Cancer* **122**, X-XI. (doi:10.1002/ijc.23347).

237. Little M.P., Charles M.W. 1990 Bomb survivor selection and consequences for estimates of population cancer risks. *Health Phys* **59**, 765-775.

238. Little M.P., Wakeford R., Charles M.W. 1994 An analysis of leukaemia, lymphoma and other malignancies together with certain categories of non-cancer mortality in the first generation offspring (F1) of the Japanese bomb survivors. *J Radiol Prot* **14**, 203.

239. Little M.P., Boice J.D., Jr. 1999 Comparison of breast cancer incidence in the Massachusetts tuberculosis fluoroscopy cohort and in the Japanese atomic bomb survivors. *Radiat Res* **151**, 218-224.

240. Little M.P. 2001 Comparison of the risks of cancer incidence and mortality following radiation therapy for benign and malignant disease with the cancer risks observed in the Japanese A-bomb survivors. *Int J Radiat Biol* **77**, 431-464. (doi:10.1080/09553000010022634).

241. Little M.P. 2002a Absence of evidence for differences in the dose-response for cancer and non-cancer endpoints by acute injury status in the Japanese atomic-bomb survivors. *Int J Radiat Biol* **78**, 1001-1010. (doi:10.1080/0955300021000013803).

242. Little M.P. 2002b Comments on the paper: Microsatellite instability in acute myelocytic leukaemia developed from A-bomb survivors. *Int J Radiat Biol* **78**, 441-443. (doi:10.1080/09553000110097956).

243. Little M.P. 2003 Risks associated with ionizing radiation *British Medical Bulletin* **68**, 259-275.

244. Little M.P., Tawn E.J., Tzoulaki I., Wakeford R., Hildebrandt G., Paris F., Tapio S., Elliott P. 2008b A systematic review of epidemiological associations between low and moderate doses of ionizing radiation and late cardiovascular effects, and their possible mechanisms. *Radiat Res* **169**, 99-109. (doi:10.1667/rr1070.1).

245. Little M.P., Wakeford R., Tawn E.J., Bouffler S.D., Berrington de Gonzalez A. 2009 Risks associated with low doses and low dose rates of ionizing radiation: why linearity may be (almost) the best we can do. *Radiology* **251**, 6-12. (doi:10.1148/radiol.2511081686).

246. Little M.P. 2010 Do non-targeted effects increase or decrease low dose risk in relation to the linear-non-threshold (LNT) model? *Mutat Res* **687**, 17-27. (doi:10.1016/j.mrfmmm.2010.01.008).

247. Little M.P., Azizova T.V., Bazyka D., Bouffler S.D., Cardis E., Chekin S., Chumak V.V., Cucinotta F.A., de Vathaire F., Hall P., et al. 2012 Systematic review and meta-analysis of circulatory disease from exposure to low-level ionizing radiation and estimates of potential population mortality risks. *Environ Health Perspect* **120**, 1503-1511. (doi:10.1289/ehp.1204982).

248. Little M.P., Goodhead D.T., Bridges B.A., Bouffler S.D. 2013 Evidence relevant to untargeted and transgenerational effects in the offspring of irradiated parents. *Mutat Res* **753**, 50-67. (doi:10.1016/j.mrrev.2013.04.001).

249. Little M.P. 2013 A review of non-cancer effects, especially circulatory and ocular diseases. *Radiat Environ Biophys* **52**, 435-449. (doi:10.1007/s00411-013-0484-7).

250. Little M.P. 2015 Germline minisatellite mutations in the offspring of irradiated parents. *J Radiol Prot* **35**, E1-4. (doi:10.1088/0952-4746/35/1/E1).

251. Little M.P. 2016 Radiation and circulatory disease. *Mutat Res* **770**, 299-318. (doi:10.1016/j.mrrev.2016.07.008).

252. Livshits L.A., Malyarchuk S.G., Kravchenko S.A., Matsuka G.H., Lukyanova E.M., Antipkin Y.G., Arabskaya L.P., Petit E., Giraudeau F., Gourmelon P., et al. 2001 Children of Chernobyl cleanup workers do not show elevated rates of mutations in minisatellite alleles. *Radiat Res* **155**, 74-80.

253. Lloyd D.C., Edwards A.A., Leonard A., Deknudt G.L., Verschaeve L., Natarajan A.T., Darroudi F., Obe G., Palitti F., Tanzarella C., et al. 1992 Chromosomal aberrations in human lymphocytes induced in vitro by very low doses of X-rays. *Int J Radiat Biol* **61**, 335-343.

254. Lobrich M., Jeggo P.A. 2007 The impact of a negligent G2/M checkpoint on genomic instability and cancer induction. *Nat Rev Cancer* **7**, 861-869. (doi:10.1038/nrc2248).

255. Loganovsky K., Havenaar J.M., Tintle N.L., Guey L.T., Kotov R., Bromet E.J. 2008 The mental health of clean-up workers 18 years after the Chernobyl accident. *Psychol Med* **38**, 481-488. (doi:10.1017/s0033291707002371).

256. Lomax M., Folkes L., O'Neill P. 2013 Biological consequences of radiation-induced DNA damage: relevance to radiotherapy. *Clinical Oncology* **25**, 578-585.

257. Lubin J.H. 1994 *Radon and lung cancer risk: a joint analysis of 11 underground miners studies*, U.S. Dept. of Health and Human Services, Public Health Service, National Institute of Health.

258. Lubin J.H., Boice J.D., Jr., Edling C., Hornung R.W., Howe G.R., Kunz E., Kusiak R.A., Morrison H.I., Radford E.P., Samet J.M., et al. 1995 Lung cancer in radon-exposed miners and estimation of risk from indoor exposure. *J Natl Cancer Inst* **87**, 817-827.

259. Lubin J.H., Wang Z.Y., Boice J.D., Xu Z.Y., Blot W.J., De Wang L., Kleinerman R.A. 2004 Risk of lung cancer and residential radon in China: pooled results of two studies. *International Journal of Cancer* **109**, 132-137.

260. Luckey T.D. 2011 Biological Effects of Ionizing Radiation: a Perspective for Japan. *Journal of American Physicians and Surgeons* **16**, 45-46.

261. Lupatsch J.E., Kuehni C.E., Niggli F., Ammann R.A., Egger M., Spycher B.D. 2015 Population mixing and the risk of childhood leukaemia in Switzerland: a census-based cohort study. *Eur J Epidemiol*, 1-12.

262. Lupatsch J.E., Kreis C., Zwahlen M., Niggli F., Ammann R.A., Kuehni C.E., Spycher B.D. 2016 Temporal association between childhood

leukaemia and population growth in Swiss municipalities. *Eur J Epidemiol*. (doi:10.1007/s10654-016-0162-6).

263. Manning G., Rothkamm K. 2013 Deoxyribonucleic acid damage-associated biomarkers of ionising radiation: current status and future relevance for radiology and radiotherapy. *Br J Radiol* **86**, 20130173. (doi:10.1259/bjr.20130173).

264. Marsh J.W., Harrison J.D., Laurier D., Blanchardon E., Paquet F., Tirmarche M. 2010 Dose conversion factors for radon: recent developments. *Health Phys* **99**, 511-516. (doi:10.1097/HP.0b013e3181d6bc19).

265. Martin L.M., Marples B., Lynch T.H., Hollywood D., Marignol L. 2013 Exposure to low dose ionising radiation: molecular and clinical consequences. *Cancer Lett* **338**, 209-218. (doi:10.1016/j.canlet.2013.05.021).

266. Matanoski G.M., Sternberg A., Elliott E.A. 1987 Does radiation exposure produce a protective effect among radiologists? *Health Physics* **52**, 637-643. (doi:10.1097/00004032-198705000-00016).

267. Mathews J.D., Forsythe A.V., Brady Z., Butler M.W., Goergen S.K., Byrnes G.B., Giles G.G., Wallace A.B., Anderson P.R., Guiver T.A., et al. 2013 Cancer risk in 680 000 people exposed to computed tomography scans in childhood or adolescence: data linkage study of 11 million Australians. *Br Med J* **346**, 18. (doi:10.1136/bmj.f2360).

268. Mathias D., Mitchel R.E.J., Barclay M., Wyatt H., Bugden M., Priest N.D., Whitman S.C., Scholz M., Hildebrandt G., Kamprad M., et al. 2015 Low-dose irradiation affects expression of inflammatory markers in the heart of ApoE -/- mice. *PLoS One* **10**, e0119661. (doi:10.1371/journal.pone.0119661).

269. Mattsson S., Nilsson M. 2015 On the estimation of radiation-induced cancer risks from very low doses of radiation and how to communicate these risks. *Radiation Protection Dosimetry*, ncv037.

270. Men T., Brennan P., Boffetta P., Zaridze D. 2003 Russian mortality trends for 1991-2001: analysis by cause and region. *BMJ* **327**, 964. (doi:10.1136/bmj.327.7421.964).

271. Mettler F.A., Upton A.C. 2008 Deterministic effects of ionizing radiation. In *Medical Effects of Radiation 3rd ed* (Philadelphia, Saunders Elsevier

272. Minamoto A., Taniguchi H., Yoshitani N., Mukai S., Yokoyama T., Kumagami T., Tsuda Y., Mishima H.K., Amemiya T., Nakashima E., et al. 2004 Cataract in atomic bomb survivors. *Int J Radiat Biol* **80**, 339-345. (doi:10.1080/09553000410001680332).

273. Mobbs S.F., Muirhead C.R., Harrison J.D. 2011 Risks from ionising radiation: an HPA viewpoint paper for Safegrounds. *J Radiol Prot* **31**, 289-307. (doi:10.1088/0952-4746/31/3/r01).

274. Mohan A.K., Hauptmann M., Freedman D.M., Ron E., Matanoski G.M., Lubin J.H., Alexander B.H., Boice J.D., Jr., Doody M.M., Linet M.S. 2003 Cancer and other causes of mortality among radiologic technologists in the United States. *Int J Cancer* **103**, 259-267. (doi:10.1002/ijc.10811).

275. Mohner M., Gellissen J., Marsh J.W., Gregoratto D. 2010 Occupational and diagnostic exposure to ionizing radiation and leukemia risk among German uranium miners. *Health Phys* **99**, 314-321. (doi:10.1097/HP.0b013e3181cd6536).

276. Monceau V., Meziani L., Strup-Perrot C., Morel E., Schmidt M., Haagen J., Escoubet B., Dörr W., Vozenin M.-C. 2013 Enhanced sensitivity to low dose irradiation of ApoE−/− mice mediated by early pro-inflammatory profile and delayed activation of the TGFβ1 cascade Involved in fibrogenesis. *PLoS One* **8**, e57052. (doi:10.1371/journal.pone.0057052).

277. Moore S., Stanley F.K., Goodarzi A.A. 2014 The repair of environmentally relevant DNA double strand breaks caused by high linear energy transfer irradiation--no simple task. *DNA Repair (Amst)* **17**, 64-73. (doi:10.1016/j.dnarep.2014.01.014).

278. Morgan W.F. 2003a Non-targeted and delayed effects of exposure to ionizing radiation: I. Radiation-induced genomic instability and bystander effects in vitro. *Radiat Res* **159**, 567-580.

279. Morgan W.F. 2003b Non-targeted and delayed effects of exposure to ionizing radiation: II. Radiation-induced genomic instability and bystander effects in vivo, clastogenic factors and transgenerational effects. *Radiat Res* **159**, 581-596.

280. Morgan W.F., Bair W.J. 2013 Issues in low dose radiation biology: the controversy continues. A perspective. *Radiat Res* **179**, 501-510. (doi:10.1667/rr3306.1).

281. Moseeva M.B., Azizova T.V., Grigoryeva E.S., Haylock R. 2014 Risks of circulatory diseases among Mayak PA workers with radiation doses estimated using the improved Mayak Worker Dosimetry System 2008. *Radiat Environ Biophys* **53**, 469-477. (doi:10.1007/s00411-014-0517-x).

282. Muggenburg B.A., Guilmette R.A., Hahn F.F., Diel J.H., Mauderly J.L., Seilkop S.K., Boecker B.B. 2008 Radiotoxicity of inhaled (239)PuO(2) in dogs. *Radiat Res* **170**, 736-757. (doi:10.1667/rr1409.1).

283. Mughal S.K., Myazin A.E., Zhavoronkov L.P., Rubanovich A.V., Dubrova Y.E. 2012 The dose and dose-rate effects of paternal irradiation on transgenerational instability in mice: a radiotherapy connection. *PLoS One* **7**, e41300. (doi:10.1371/journal.pone.0041300).

284. Muirhead C.R., O'Hagan J.A., Haylock R.G.E., Phillipson M.A., Willcock T., Berridge G.L.C., Zhang W. 2009 Mortality and cancer incidence following occupational radiation exposure: third analysis of the National Registry for Radiation Workers. *British Journal of Cancer* **100**, 206-212. (doi:10.1038/sj.bjc.6604825).

285. Muirhead C.R. 2013 Childhood leukaemia near nuclear power plants. *British Journal of Cancer* **109**, 2763-2764. (doi:10.1038/bjc.2013.674).

286. Muirhead C.R. 2015 Response to 'Are the studies on cancer risk from CT scans biased by indication? Elements of answer from a large-scale cohort study in France'. *British Journal of Cancer* **112**, 1841-1842. (doi:10.1038/bjc.2015.106).

287. Mullenders L., Atkinson M., Paretzke H., Sabatier L., Bouffler S. 2009 Assessing cancer risks of low-dose radiation. *Nat Rev Cancer* **9**, 596-604. (doi:10.1038/nrc2677).

288. Muller W.U. 2015 Current discussions of DDREF, cataracts, circulatory diseases and dose limits. *Radiation Protection Dosimetry* **164**, 34-37. (doi:10.1093/rpd/ncu311).

289. Muller W.U., Giussani A., Ruhm W., Lecomte J.F., Harrison J., Kreuzer M., Sobotzki C., Breckow J. 2016 Current knowledge on radon risk: implications for practical radiation protection? Radon workshop, 1/2 December 2015, Bonn, BMUB *Radiat Environ Biophys* **55**, 267-280. (doi:10.1007/s00411-016-0657-2).

290. Mutscheller A. 1925 Physical Standards of Protection Against Roentgen Ray Dangers. *Am Jour Roentgenol and Rad Ther* **XIII**.

291. Nagataki S., Kasagi F. 2015 INWORKS study: risk of leukaemia from protracted radiation exposure. *Lancet Haematol* **2**, e404. (doi:10.1016/S2352-3026(15)00131-3).

292. Nagataki S., Takamura N. 2016 Radioactive Doses - Predicted and Actual - and Likely Health Effects. *Clin Oncol (R Coll Radiol)* **28**, 245-254. (doi:10.1016/j.clon.2015.12.028).

293. Nair R.R.K., Rajan B., Akiba S., Jayalekshmi P., Nair M.K., Gangadharan P., Koga T., Morishima H., Nakamura S., Sugahara T. 2009 Background radiation and cancer incidence in Kerala, India - Karunagappally cohort study. *Health Physics* **96**, 55-66. (doi:10.1097/01.hp.0000327646.54923.11).

294. Nakamura N. 2006 Genetic effects of radiation in atomic-bomb survivors and their children: past, present and future. *J Radiat Res* **47 Suppl B**, B67-73.

295. Nakamura N., Suyama A., Noda A., Kodama Y. 2013 Radiation effects on human heredity. *Annu Rev Genet* **47**, 33-50. (doi:10.1146/annurev-genet-111212-133501).

296. Nakashima E., Neriishi K., Minamoto A. 2006 A reanalysis of atomic-bomb cataract data, 2000-2002: a threshold analysis. *Health Phys* **90**, 154-160.

297. National Research Council. 1995 *Radiation Dose Reconstruction for Epidemiologic Uses*. Washington, DC, The National Academies Press.

298. NCRP (National Council on Radiation Protection and Measurements). 2009 Ionizing radiation exposure of the population of the United States. Bethseda, MD.

299. NCRP (National Council on Radiation Protection and Measurements). 2015 Health effects of low doses of radiation: perspectives on integrating radiation biology and epidemiology. Bethseda, MD.

300. Neel J.V., Schull W.J., Awa A.A., Satoh C., Kato H., Otake M., Yoshimoto Y. 1990 The children of parents exposed to atomic bombs: estimates of the genetic doubling dose of radiation for humans. *Am J Hum Genet* **46**, 1053-1072.

301. Neel J.V., Schull W.J. 1991 *The Children of atomic bomb survivors : a genetic study*. Washington, D.C., National Academy Press.

302. Nekolla E.A., Kreisheimer M., Kellerer A.M., Kuse-Isingschulte M., Gossner W., Spiess H. 2000 Induction of malignant bone tumors in radium-224 patients: risk estimates based on the improved dosimetry. *Radiat Res* **153**, 93-103.

303. Neriishi K., Nakashima E., Minamoto A., Fujiwara S., Akahoshi M., Mishima H.K., Kitaoka T., Shore R.E. 2007 Postoperative cataract cases among atomic bomb survivors: radiation dose response and threshold. *Radiat Res* **168**, 404-408. (doi:10.1667/RR0928.1).

304. Nikkila A., Erme S., Arvela H., Holmgren O., Raitanen J., Lohi O., Auvinen A. 2016 Background radiation and childhood leukemia: A nationwide register-based case-control study. *Int J Cancer* **139**, 1975-1982. (doi:10.1002/ijc.30264).

305. Niles K. 2014 The Hanford cleanup: What's taking so long? *Bulletin of the Atomic Scientists* **70**, 37-48. (doi:10.1177/0096340214539115).

306. Niwa O. 2010 Roles of stem cells in tissue turnover and radiation carcinogenesis. *Radiat Res* **174**, 833-839. (doi:10.1667/rr1970.1).

307. Normile D. 2016 Epidemic of fear. *Science* **351**, 1022-1023. (doi:10.1126/science.351.6277.1022).

308. Noshchenko A.G., Zamostyan P.V., Bondar O.Y., Drozdova V.D. 2002 Radiation - induced leukemia risk among those aged 0–20 at the time of the Chernobyl accident: A case - control study in the Ukraine. *International Journal of Cancer* **99**, 609-618.

309. Noshchenko A.G., Bondar O.Y., Drozdova V.D. 2010 Radiation-induced leukemia among children aged 0-5 years at the time of the Chernobyl accident. *Int J Cancer* **127**, 412-426. (doi:10.1002/ijc.24834).

310. Oatway W.B., Jones A.L., Holmes S., Watson S., Cabianca T. 2016 Ionising Radiation Exposure of the UK Population: 2010 Review. UK, Public Health England).

311. Ohtaki K., Kodama Y., Nakano M., Itoh M., Awa A.A., Cologne J., Nakamura N. 2004 Human fetuses do not register chromosome damage inflicted by radiation exposure in lymphoid precursor cells except for a small but significant effect at low doses. *Radiat Res* **161**, 373-379.

312. Organo C., Ellard A., Fenton D., Synnott H., O'Colmain M., Prenter S., O'Reilly S., Colgan P.A. 2004 High radon concentrations in a house near Castleisland, County Kerry (Ireland)--identification, remediation and post-remediation. *J Radiol Prot* **24**, 107-120.

313. Ostroumova E., Brenner A., Oliynyk V., McConnell R., Robbins J., Terekhova G., Zablotska L., Likhtarev I., Bouville A., Shpak V., et al. 2009 Subclinical hypothyroidism after radioiodine exposure: Ukrainian-American cohort study of thyroid cancer and other thyroid diseases after the Chornobyl accident (1998-2000). *Environ Health Perspect* **117**, 745-750. (doi:10.1289/ehp.0800184).

314. Ostroumova E., Rozhko A., Hatch M., Furukawa K., Polyanskaya O., McConnell R.J., Nadyrov E., Petrenko S., Romanov G., Yauseyenka V., et al. 2013 Measures of thyroid function among Belarusian children and adolescents exposed to iodine-131 from the accident at the Chernobyl nuclear plant. *Environ Health Perspect* **121**, 865-871. (doi:10.1289/ehp.1205783).

315. Ostroumova E., Hatch M., Brenner A., Nadyrov E., Veyalkin I., Polyanskaya O., Yauseyenka V., Polyakov S., Levin L., Zablotska L., et al. 2016 Non-thyroid cancer incidence in Belarusian residents exposed to Chernobyl fallout in childhood and adolescence: Standardized Incidence Ratio analysis, 1997-2011. *Environ Res* **147**, 44-49. (doi:10.1016/j.envres.2016.01.025).

316. Otake M., Schull W.J., Neel J.V. 1990 Congenital malformations, stillbirths, and early mortality among the children of atomic bomb survivors: a reanalysis. *Radiat Res* **122**, 1-11.

317. Otake M., Schull W.J. 1998 Radiation-related brain damage and growth retardation among the prenatally exposed atomic bomb survivors. *Int J Radiat Biol* **74**, 159-171.

318. Ozasa K., Shimizu Y., Suyama A., Kasagi F., Soda M., Grant E.J., Sakata R., Sugiyama H., Kodama K. 2012 Studies of the mortality of atomic bomb survivors, Report 14, 1950-2003: an overview of cancer and noncancer diseases. *Radiat Res* **177**, 229-243. *Errata* in Vol 179, e40-41, 2013.

319. Ozasa K., Takahashi I., Grant E.J. 2016 Radiation-related risks of non-cancer outcomes in the atomic bomb survivors. *Ann ICRP* **45**, 253-261. (doi:10.1177/0146645316629318).

320. Ozasa K., Takahashi I., Grant E.J., Kodama K. 2017 Cardiovascular disease among atomic bomb survivors. *Int J Radiat Biol*, 1-6. (doi:10.1080/09553002.2017.1290849).

321. Parkin D.M., Clayton D., Black R.J., Masuyer E., Friedl H.P., Ivanov E., Sinnaeve J., Tzvetansky C.G., Geryk E., Storm H.H., et al. 1996 Childhood leukaemia in Europe after Chernobyl: 5 year follow-up. *Br J Cancer* **73**, 1006-1012.

322. Paunesku T., Haley B., Brooks A., Woloschak G.E. 2017 Biological basis of radiation protection needs rejuvenation. *Int J Radiat Biol*, 1-8. (doi:10.1080/09553002.2017.1294773).

323. Peacock J., Ashton A., Bliss J., Bush C., Eady J., Jackson C., Owen R., Regan J., Yarnold J. 2000 Cellular radiosensitivity and complication risk after curative radiotherapy. *Radiotherapy and Oncology* **55**, 173-178.

324. Pearce F. 2015 Shocking state of world's riskiest nuclear waste site. *New Scientist* **225**.

325. Pearce M.S., Salotti J.A., Little M.P., McHugh K., Lee C., Kim K.P., Howe N.L., Ronckers C.M., Rajaraman P., Craft A.W., et al. 2012 Radiation exposure from CT scans in childhood and subsequent risk of leukaemia and brain tumours: a retrospective cohort study. *Lancet* **380**, 499-505. (doi:10.1016/s0140-6736(12)60815-0).

326. Pernot E., Hall J., Baatout S., Benotmane M.A., Blanchardon E., Bouffler S., El Saghire H., Gomolka M., Guertler A., Harms-Ringdahl M., et al. 2012 Ionizing radiation biomarkers for potential use in epidemiological studies. *Mutat Res* **751**, 258-286. (doi:10.1016/j.mrrev.2012.05.003).

327. Pernot E., Cardis E., Badie C. 2014 Usefulness of saliva samples for biomarker studies in radiation research. *Cancer Epidemiol Biomarkers Prev* **23**, 2673-2680. (doi:10.1158/1055-9965.epi-14-0588).

328. Petridou E., Trichopoulos D., Dessypris N., Flytzani V., Haidas S., Kalmanti M., Koliouskas D., Kosmidis H., Piperopoulou F., Tzortzatou F. 1996 Infant leukaemia after in utero exposure to radiation from Chernobyl. *Nature* **382**, 352-353. (doi:10.1038/382352a0).

329. Pierce D.A., Shimizu Y., Preston D.L., Vaeth M., Mabuchi K. 1996 Studies of the mortality of atomic bomb survivors. Report 12, Part I. Cancer: 1950-1990. *Radiat Res* **146**, 1-27.

330. Pierce D.A., Sharp G.B., Mabuchi K. 2003 Joint effects of radiation and smoking on lung cancer risk among atomic bomb survivors. *Radiat Res* **159**, 511-520.

331. Pohl-Ruling J., Fischer P., Haas O., Obe G., Natarajan A.T., van Buul P.P., Buckton K.E., Bianchi N.O., Larramendy M., Kucerova M., et al. 1983 Effect of low-dose acute X-irradiation on the frequencies of chromosomal aberrations in human peripheral lymphocytes in vitro. *Mutat Res* **110**, 71-82.

332. Preston D.L., Shimizu Y., Pierce D.A., Suyama A., Mabuchi K. 2003 Studies of mortality of atomic bomb survivors. Report 13: Solid cancer and noncancer disease mortality: 1950-1997. *Radiat Res* **160**, 381-407.

333. Preston D.L., Ron E., Tokuoka S., Funamoto S., Nishi N., Soda M., Mabuchi K., Kodama K. 2007 Solid cancer incidence in atomic bomb survivors: 1958-1998. *Radiat Res* **168**, 1-64. (doi:10.1667/rr0763.1).

334. Preston D.L., Cullings H., Suyama A., Funamoto S., Nishi N., Soda M., Mabuchi K., Kodama K., Kasagi F., Shore R.E. 2008 Solid Cancer Incidence in Atomic Bomb Survivors Exposed In Utero or as Young Children. *Journal of the National Cancer Institute* **100**, 428-436. (doi:10.1093/jnci/djn045).

335. Preston R.J., Boice Jr J.D., Brill A.B., Chakraborty R., Conolly R., Hoffman F.O., Hornung R.W., Kocher D.C., Land C.E., Shore R.E. 2013 Uncertainties in estimating health risks associated with exposure to ionising radiation. *J Radiol Prot* **33**, 573.

336. Preston R.J. 2017 Can radiation research impact the estimation of risk? *Int J Radiat Biol*, (February, online) 1-6. (doi:10.1080/09553002.2017.1290848).

337. Prysyazhnyuk A., Gristchenko V., Fedorenko Z., Gulak L., Fuzik M., Slipenyuk K., Tirmarche M. 2007 Twenty years after the Chernobyl accident: solid cancer incidence in various groups of the Ukrainian population. *Radiat Environ Biophys* **46**, 43-51. (doi:10.1007/s00411-007-0093-4).

338. Pukkala E., Kesminiene A., Poliakov S., Ryzhov A., Drozdovitch V., Kovgan L., Kyyronen P., Malakhova I.V., Gulak L., Cardis E. 2006 Breast cancer in Belarus and Ukraine after the Chernobyl accident. *Int J Cancer* **119**, 651-658. (doi:10.1002/ijc.21885).

339. Raaschou-Nielsen O., Andersen C.E., Andersen H.P., Gravesen P., Lind M., Schuz J., Ulbak K. 2008 Domestic radon and childhood cancer in Denmark. *Epidemiology* **19**, 536-543. (doi:10.1097/EDE.0b013e318176bfcd).

340. Rahu K., Rahu M., Tekkel M., Bromet E. 2006a Suicide risk among Chernobyl cleanup workers in Estonia still increased: an updated cohort study. *Ann Epidemiol* **16**, 917-919. (doi:10.1016/j.annepidem.2006.07.006).

341. Rahu K., Auvinen A., Hakulinen T., Tekkel M., Inskip P.D., Bromet E.J., Boice J.D., Jr., Rahu M. 2013a Chernobyl cleanup workers from Estonia: follow-up for cancer incidence and mortality. *J Radiol Prot* **33**, 395-411. (doi:10.1088/0952-4746/33/2/395).

342. Rahu K., Hakulinen T., Smailyte G., Stengrevics A., Auvinen A., Inskip P.D., Boice J.D., Jr., Rahu M. 2013b Site-specific cancer risk in the Baltic cohort of Chernobyl cleanup workers, 1986-2007. *Eur J Cancer* **49**, 2926-2933. (doi:10.1016/j.ejca.2013.04.014).

343. Rahu M., Rahu K., Auvinen A., Tekkel M., Stengrevics A., Hakulinen T., Boice J.D., Jr., Inskip P.D. 2006b Cancer risk among Chernobyl cleanup workers in Estonia and Latvia, 1986-1998. *Int J Cancer* **119**, 162-168. (doi:10.1002/ijc.21733).

344. Rericha V., Kulich M., Rericha R., Shore D.L., Sandler D.P. 2006 Incidence of leukemia, lymphoma, and multiple myeloma in Czech uranium miners: a case-cohort study. *Environ Health Perspect* **114**, 818-822.

345. Richardson D., Sugiyama H., Nishi N., Sakata R., Shimizu Y., Grant E.J., Soda M., Hsu W.L., Suyama A., Kodama K., et al. 2009 Ionizing radiation and leukemia mortality among Japanese Atomic Bomb Survivors, 1950-2000. *Radiat Res* **172**, 368-382. (doi:10.1667/rr1801.1).

346. Richardson D.B., Wing S., Schroeder J., Schmitz-Feuerhake I., Hoffmann W. 2005 Ionizing radiation and chronic lymphocytic leukemia. *Environ Health Perspect* **113**, 1-5.

347. Richardson D.B., Cardis E., Daniels R.D., Gillies M., O'Hagan J.A., Hamra G.B., Haylock R., Laurier D., Leuraud K., Moissonnier M. 2015 Risk of cancer from occupational exposure to ionising radiation: retrospective cohort study of workers in France, the United Kingdom, and the United States (INWORKS). *Br Med J* **351**, h5359.

348. Roberson J.D., 2nd, Burnett O.L., 3rd, Robin N. 2016 Radiogenomics: towards a personalized radiation oncology. *Curr Opin Pediatr* **28**, 713-717. (doi:10.1097/mop.0000000000000408).

349. Romanenko A.Y., Finch S.C., Hatch M., Lubin J.H., Bebeshko V.G., Bazyka D.A., Gudzenko N., Dyagil I.S., Reiss R.F., Bouville A., et al. 2008 The Ukrainian-American study of leukemia and related disorders among Chornobyl cleanup workers from Ukraine: III. Radiation risks. *Radiat Res* **170**, 711-720. (doi:10.1667/rr1404.1).

350. Rombouts C., Aerts A., Quintens R., Baselet B., El-Saghire H., Harms-Ringdahl M., Haghdoost S., Janssen A., Michaux A., Yentrapalli R., et al. 2014 Transcriptomic profiling suggests a role for IGFBP5 in premature senescence of endothelial cells after chronic low dose rate irradiation. *Int J Radiat Biol* **90**, 560-574. (doi:10.3109/09553002.2014.905724).

351. Ronckers C.M., Land C.E., Miller J.S., Stovall M., Lonstein J.E., Doody M.M. 2010 Cancer mortality among women frequently exposed to radiographic examinations for spinal disorders. *Radiat Res* **174**, 83-90. (doi:10.1667/rr2022.1).

352. Rowland R.E., Stehney A.F., Lucas H.F., Jr. 1978 Dose-Response Relationships for Female Radium Dial Workers. *Radiat Res* **76**, 368-383. (doi:10.2307/3574786).

353. Ruhm W., Woloschak G.E., Shore R.E., Azizova T.V., Grosche B., Niwa O., Akiba S., Ono T., Suzuki K., Iwasaki T., et al. 2015 Dose and dose-rate effects of ionizing radiation: a discussion in the light of radiological protection. *Radiat Environ Biophys* **54**, 379-401. (doi:10.1007/s00411-015-0613-6).

354. Ruhm W., Azizova T.V., Bouffler S.D., Little M.P., Shore R.E., Walsh L., Woloschak G.E. 2016 Dose-rate effects in radiation biology and radiation protection. *Ann ICRP*. (doi:10.1177/0146645316629336).

355. Ruhm W., Eidemuller M., Kaiser J.C. 2017 Biologically-based mechanistic models of radiation-related carcinogenesis applied to epidemiological data. *Int J Radiat Biol*, 1-25. (doi:10.1080/09553002.2017.1310405).

356. Sachs R.K., Chen A.M., Brenner D.J. 1997 Review: proximity effects in the production of chromosome aberrations by ionizing radiation. *Int J Radiat Biol* **71**, 1-19.

357. Sacks B., Meyerson G., Siegel J.A. 2016 Epidemiology without biology: false paradigms, unfounded assumptions, and specious statistics in radiation science (with commentaries by Inge Schmitz-

Feuerhake and Christopher Busby and a reply by the Authors). *Biological Theory* **11**, 69-101. (doi:10.1007/s13752-016-0244-4).

358. Sagne C., Marcel V., Amadou A., Hainaut P., Olivier M., Hall J. 2013 A meta-analysis of cancer risk associated with the TP53 intron 3 duplication polymorphism (rs17878362): geographic and tumor-specific effects. *Cell Death Dis* **4**, e492. (doi:10.1038/cddis.2013.24).

359. Sagne C., Marcel V., Bota M., Martel-Planche G., Nobrega A., Palmero E.I., Perriaud L., Boniol M., Vagner S., Cox D.G., et al. 2014 Age at cancer onset in germline TP53 mutation carriers: association with polymorphisms in predicted G-quadruplex structures. *Carcinogenesis* **35**, 807-815. (doi:10.1093/carcin/bgt381).

360. Sakata R., Grant E.J., Furukawa K., Misumi M., Cullings H., Ozasa K., Shore R.E. 2014 Long-term effects of the rain exposure shortly after the atomic bombings in Hiroshima and Nagasaki. *Radiat Res* **182**, 599-606. (doi:10.1667/rr13822.1).

361. Sallmen M., Bonde J.P., Lindbohm M.L., Kristensen P. 2016 Rejoinder: The Authors Respond. *Epidemiology* **27**, 462-463. (doi:10.1097/EDE.0000000000000458).

362. Salomaa S., Averbeck D., Ottolenghi A., Sabatier L., Bouffler S., Atkinson M., Jourdain J.R. 2015 European low-dose radiation risk research strategy: future of research on biological effects at low doses. *Radiat Prot Dosimetry* **164**, 38-41. (doi:10.1093/rpd/ncu350).

363. Sankaranarayanan K., Chakraborty R. 2000 Ionizing radiation and genetic risks. XIII. Summary and synthesis of papers VI to XII and estimates of genetic risks in the year 2000. *Mutat Res* **453**, 183-197.

364. Sanlorenzo M., Wehner M.R., Linos E., Kornak J., Kainz W., Posch C., Vujic I., Johnston K., Gho D., Monico G., et al. 2015 The risk of melanoma in airline pilots and cabin crew: a meta-analysis. *JAMA Dermatol* **151**, 51-58. (doi:10.1001/jamadermatol.2014.1077).

365. Satoh C., Takahashi N., Asakawa J., Kodaira M., Kuick R., Hanash S.M., Neel J.V. 1996 Genetic analysis of children of atomic bomb survivors. *Environ Health Perspect* **104 Suppl 3**, 511-519.

366. Sawada S. 2007 Cover-up of the effects of internal exposure by residual radiation from the atomic bombing of Hiroshima and Nagasaki. *Med Confl Surviv* **23**, 58-74. (doi:10.1080/13623690601084617).

367. Schanz S., Flockerzi E., Schuberth K., Rube C. 2014 Genetically defined DNA repair capacity determines the extent of DNA damage accumulation in healthy mouse tissues after very low doses of ionizing radiation. *J Carcinog Mutagen* **5**, 1-8.

368. Schmiedel S., Blettner M., Kaatsch P., Schuz J. 2010 Spatial clustering and space-time clusters of leukemia among children in Germany, 1987-2007. *Eur J Epidemiol* **25**, 627-633. (doi:10.1007/s10654-010-9488-7).

369. Schmitz-Feuerhake I., Schroder H., Dannheim B., Grellbuchtmann I., Heimers A., Hoffmann W., Nahrmann A., Tomalik P. 1993 Leukemia near water nuclear reactor. *Lancet* **342**, 1484-1484. (doi:10.1016/0140-6736(93)92959-w).

370. Schonfeld S.J., Krestinina L.Y., Epifanova S., Degteva M.O., Akleyev A.V., Preston D.L. 2013 Solid cancer mortality in the Techa River cohort (1950-2007). *Radiat Res* **179**, 183-189. (doi:10.1667/rr2932.1).

371. Schubauer-Berigan M.K., Daniels R.D., Pinkerton L.E. 2009 Radon exposure and mortality among white and American Indian uranium miners: an update of the Colorado Plateau cohort. *Am J Epidemiol* **169**, 718-730. (doi:10.1093/aje/kwn406).

372. Schull W.J., Otake M. 1999 Cognitive function and prenatal exposure to ionizing radiation. *Teratology* **59**, 222-226. (doi:10.1002/(SICI)1096-9926(199904)59:4<222::AID-TERA6>3.0.CO;2-M).

373. Searle A.G. 1974 Mutation Induction in Mice. In *Advances in radiation biology* (eds. John T. Lett H.A., Max Z.), pp. 131-207, Elsevier.

374. Sermage-Faure C., Laurier D., Goujon-Bellec S., Chartier M., Guyot-Goubin A., Rudant J., Hemon D., Clavel J. 2012 Childhood leukemia around French nuclear power plants - the Geocap study, 2002-2007. *Int J Cancer* **131**, E769-E780. (doi:10.1002/ijc.27425).

375. Shah D.J., Sachs R.K., Wilson D.J. 2012 Radiation-induced cancer: a modern view. *Br J Radiol* **85**, e1166-1173. (doi:10.1259/bjr/25026140).

376. Shantha E., Lewis C., Nghiem P. 2015 Why do airline pilots and flight crews have an increased incidence of melanoma? *JAMA Oncol* **1**, 829-830. (doi:10.1001/jamaoncol.2015.0933).

377. Shellabarger C.J., Chmelevsky D., Kellerer A.M. 1980 Induction of mammary neoplasms in the Sprague-Dawley rat by 430keV neutrons and X-rays. *J Natl Cancer Inst* **64**, 821-833.

378. Sherborne A.L., Davidson P.R., Yu K., Nakamura A.O., Rashid M., Nakamura J.L. 2015 Mutational analysis of ionizing radiation induced neoplasms. *Cell Rep* **12**, 1915-1926. (doi:10.1016/j.celrep.2015.08.015).

379. Shibata A., Jeggo P.A. 2014 DNA double-strand break repair in a cellular context. *Clin Oncol (R Coll Radiol)* **26**, 243-249. (doi:10.1016/j.clon.2014.02.004).

380. Shibata Y. 2016 Re: thyroid cancer among young people in Fukushima. *Epidemiology* **27**, e19-20. (doi:10.1097/EDE.0000000000000461).

381. Shimizu Y., Kodama K., Nishi N., Kasagi F., Suyama A., Soda M., Grant E.J., Sugiyama H., Sakata R., Moriwaki H., et al. 2010 Radiation exposure and circulatory disease risk: Hiroshima and Nagasaki atomic bomb survivor data, 1950-2003. *BMJ* **340**, b5349. (doi:10.1136/bmj.b5349).

382. Shore R., Walsh L., Azizova T., Ruhm W. 2017 Risk of solid cancer in low dose-rate radiation epidemiological studies and the dose-rate effectiveness factor. *Int J Radiat Biol*, 1-42. (doi:10.1080/09553002.2017.1319090).

383. Shore R.E. 2009 Low-dose radiation epidemiology studies: status and issues. *Health Physics* **97**, 481-486.

384. Shore R.E., Neriishi K., Nakashima E. 2010 Epidemiological studies of cataract risk at low to moderate radiation doses: (not) seeing is believing. *Radiat Res* **174**, 889-894. (doi:10.1667/RR1884.1).

385. Shore R.E. 2014 Radiation impacts on human health: certain, fuzzy, and unknown. *Health Physics* **106**, 196-205. (doi:10.1097/hp.0000000000000021).

386. Shore R.E. 2016a Radiation and cataract risk: Impact of recent epidemiologic studies on ICRP judgments. *Mutat Res* **770**, 231-237. (doi:10.1016/j.mrrev.2016.06.006).

387. Shore R.E. 2016b Radiation and cataract risk: Impact of recent epidemiologic studies on ICRP judgments. *Mutation Research/Reviews in Mutation Research* **770, Part B**, 231-237. (doi:http://dx.doi.org/10.1016/j.mrrev.2016.06.006).

388. Sigurdson A.J., Ron E. 2004 Cosmic radiation exposure and cancer risk among flight crew. *Cancer Investigation* **22**, 743-761. (doi:10.1081/cnv-200032767).

389. Sigurdson A.J., Stram D.O. 2012 Genetic predisposition to radiation-related cancer and potential implications for risk assessment. *Ann ICRP* **41**, 108-116. (doi:10.1016/j.icrp.2012.06.030).

390. Simon S., Bouville A., Land C. 2006 Fallout from nuclear weapons tests and cancer risks exposures 50 years ago still have health

implications today that will continue into the future. *American New Scientist* **94**, 48-57.

391. Simon S.L., Bouville A., Land C.E., Beck H.L. 2010 Radiation doses and cancer risks in the Marshall Islands associated with exposure to radioactive fallout from Bikini and Enewetak nuclear weapons tests: summary. *Health Phys* **99**, 105-123. (doi:10.1097/HP.0b013e3181dc523c).

392. Simon S.L., Bouville A. 2015 Health effects of nuclear weapons testing. *The Lancet* **386**, 407-409.

393. Sistrom C.L., Garvan C.W. 2004 Proportions, Odds, and Risk. *Radiology* **230**, 12-19. (doi:doi:10.1148/radiol.2301031028).

394. Slebos R.J., Little R.E., Umbach D.M., Antipkin Y., Zadaorozhnaja T.D., Mendel N.A., Sommer C.A., Conway K., Parrish E., Gulino S., et al. 2004 Mini-and microsatellite mutations in children from Chernobyl accident cleanup workers. *Mutat Res* **559**, 143-151. (doi:10.1016/j.mrgentox.2004.01.003).

395. Smith J.T. 2007 Are passive smoking, air pollution and obesity a greater mortality risk than major radiation incidents? *BMC Public Health* **7**, 49. (doi:10.1186/1471-2458-7-49).

396. Sokolnikov M., Preston D., Gilbert E., Schonfeld S., Koshurnikova N. 2015 Radiation effects on mortality from solid cancers other than lung, liver, and bone cancer in the Mayak worker cohort: 1948-2008. *PLoS One* **10**, e0117784. (doi:10.1371/journal.pone.0117784).

397. Spiers F.W., Lucas H.F., Rundo J., Anast G.A. 1983 Leukemia incidence in the United States dial workers. *Health Physics* **44**, 65-72. (doi:10.1097/00004032-198306001-00004).

398. Spycher B.D., Feller M., Zwahlen M., Roosli M., von der Weid N.X., Hengartner H., Egger M., Kuehni C.E., Swiss Paediat Oncology G., Swiss Natl Cohort Study G. 2011 Childhood cancer and nuclear power plants in Switzerland: a census-based cohort study. *Int J Epidemiol* **40**, 1247-1260. (doi:10.1093/ije/dyr115).

399. SSK (Statement of the Commission on Radiological Protection). 2008 Assessment of the Epidemiological Study on Childhood Cancer in the Vicinity of Nuclear Power Plants (KiKK Study). Bonn, Germany, Federal Ministry for the Environment, Nature Conservation and Nuclear Safety.

400. Stabin M., Xu X.G. 2014 Basic Principles in the Radiation Dosimetry of Nuclear Medicine. *Seminars in Nuclear Medicine* **44**, 162-171. (doi:http://dx.doi.org/10.1053/j.semnuclmed.2014.03.008).

401. Stebbings J.H., Lucas H.F., Stehney A.F. 1984 Mortality from cancers of major sites in female radium dial workers. *Am J Ind Med* **5**, 435-459.

402. Steiner M., Burkart W., Grosche B., Kaletsch U., Michaelis J. 1998 Trends in infant leukaemia in West Germany in relation to *in utero* exposure due to Chernobyl accident. *Radiat Environ Biophys* **37**, 87-93.

403. Stevens W., Thomas D.C., Lyon J.L., Till J.E., Kerber R.A., Simon S.L., Lloyd R.D., Elghany N.A., Preston-Martin S. 1990 Leukemia in Utah and radioactive fallout from the Nevada test site. A case-control study. *JAMA* **264**, 585-591.

404. Stewart A.M., Kneale G.W. 2000 A-bomb survivors: factors that may lead to a re-assessment of the radiation hazard. *Int J Epidemiol* **29**, 708-714.

405. Stewart F.A., Akleyev A.V., Hauer-Jensen M., Hendry J.H., Kleiman N.J., Macvittie T.J., Aleman B.M., Edgar A.B., Mabuchi K., Muirhead C.R., et al. 2012 ICRP publication 118: ICRP statement on tissue reactions and early and late effects of radiation in normal tissues and organs--threshold doses for tissue reactions in a radiation protection context. *Ann ICRP* **41**, 1-322. (doi:10.1016/j.icrp.2012.02.001).

406. Stewart F.A. 2012 Mechanisms and dose-response relationships for radiation-induced cardiovascular disease. *Ann ICRP* **41**, 72-79. (doi:10.1016/j.icrp.2012.06.031).

407. Streffer C., Shore R., Konermann G., Meadows A., Uma Devi P., Preston Withers J., Holm L.E., Stather J., Mabuchi K., H R. 2003 Biological effects after prenatal irradiation (embryo and fetus). A report of the International Commission on Radiological Protection. *Ann ICRP* **33**, 5-206.

408. Sugiyama H., Misumi M., Kishikawa M., Iseki M., Yonehara S., Hayashi T., Soda M., Tokuoka S., Shimizu Y., Sakata R., et al. 2014 Skin cancer incidence among atomic bomb survivors from 1958 to 1996. *Radiat Res* **181**, 531-539. (doi:10.1667/RR13494.1).

409. Suzuki K., Yamashita S. 2012 Low-dose radiation exposure and carcinogenesis. *Jpn J Clin Oncol* **42**, 563-568. (doi:10.1093/jjco/hys078).

410. Suzuki K., Mitsutake N., Saenko V., Yamashita S. 2015 Radiation signatures in childhood thyroid cancers after the Chernobyl accident: possible roles of radiation in carcinogenesis. *Cancer Sci* **106**, 127-133. (doi:10.1111/cas.12583).

411. Suzuki S. 2016a Childhood and adolescent thyroid cancer in Fukushima after the Fukushima Daiichi nuclear power plant accident: 5 years on. *Clinical Oncology* **28**, 263-271.

412. Suzuki S. 2016b Re: thyroid cancer among young people in Fukushima. *Epidemiology* **27**, e19. (doi:10.1097/EDE.0000000000000462).

413. Swift M., Morrell D., Massey R.B., Chase C.L. 1991 Incidence of cancer in 161 families affected by ataxia-telangiectasia. *N Engl J Med* **325**, 1831-1836. (doi:10.1056/nejm199112263252602).

414. Takada J., Hoshi M., Sawada S., Sakanoue M. 1983 Uranium isotopes in Hiroshima "black rain" soil. *J Radiat Res* **24**, 229-236.

415. Takahashi H., Ohira T., Yasumura S., Nollet K.E., Ohtsuru A., Tanigawa K., Abe M., Ohto H. 2016 Re: thyroid cancer among young people in Fukushima. *Epidemiology* **27**, e21. (doi:10.1097/EDE.0000000000000467).

416. Takamura N. 2016 Re: thyroid cancer among young people in Fukushima. *Epidemiology* **27**, e18. (doi:10.1097/EDE.0000000000000464).

417. Tanigawa K., Hosoi Y., Hirohashi N., Iwasaki Y., Kamiya K. 2012 Loss of life after evacuation: lessons learned from the Fukushima accident. *The Lancet* **379**, 889-891.

418. Tanigawa K., Chhem R.K. 2013 *Radiation Disaster Medicine: Perspective from the Fukushima Nuclear Accident*, Springer Science & Business Media.

419. Tao Z., Akiba S., Zha Y., Sun Q., Zou J., Li J., Liu Y., Yuan Y., Tokonami S., Morishoma H., et al. 2012 Cancer and non-cancer mortality among Inhabitants in the high background radiation area of Yangjiang, China (1979-1998). *Health Phys* **102**, 173-181. (doi:10.1097/HP.0b013e31822c7f1e).

420. Tapio S., Jacob V. 2007 Radioadaptive response revisited. *Radiat Environ Biophys* **46**, 1-12. (doi:10.1007/s00411-006-0078-8).

421. Tatsukawa Y., Cologne J.B., Hsu W.L., Yamada M., Ohishi W., Hida A., Furukawa K., Takahashi N., Nakamura N., Suyama A., et al. 2013 Radiation risk of individual multifactorial diseases in offspring of the atomic-bomb survivors: a clinical health study. *J Radiol Prot* **33**, 281-293. (doi:10.1088/0952-4746/33/2/281).

422. Tawn E.J., Curwen G.B., Rees G.S., Jonas P. 2015 Germline minisatellite mutations in workers occupationally exposed to radiation at the Sellafield nuclear facility. *J Radiol Prot* **35**, 21-36. (doi:10.1088/0952-4746/35/1/21).

423. Thacker J. 1992 Radiation-induced mutation in mammalian cells at low doses and dose rates. *Advances in radiation biology* **16**, 77-124.

424. Thierry-Chef I., Marshall M., Fix J.J., Bermann F., Gilbert E.S., Hacker C., Heinmiller B., Murray W., Pearce M.S., Utterback D., et al. 2007 The 15-Country Collaborative Study of Cancer Risk among Radiation Workers in the Nuclear Industry: study of errors in dosimetry. *Radiat Res* **167**, 380-395. (doi:10.1667/rr0552.1).

425. Thomas G.A., Symonds P. 2016 Radiation Exposure and Health Effects - is it Time to Reassess the Real Consequences? *Clin Oncol (R Coll Radiol)* **28**, 231-236. (doi:10.1016/j.clon.2016.01.007).

426. Thomas R.G. 1994 The US radium luminisers: a case for a policy of 'below regulatory concern'. *J Radiol Prot* **14**, 141.

427. Thompson R.C., United S., Pacific Northwest L. 1989 *Life-span effects of ionizing radiation in the beagle dog: a summary account of four decades of research funded by the U.S. Department of Energy and its predecessor agencies*. [S.l. : Columbus, Ohio, s.n.] ; Distributed by Battelle Press; ix, 323 p. p.

428. Tirmarche M., Harrison J.D., Laurier D., Paquet F., Blanchardon E., Marsh J.W. 2010 ICRP Publication 115. Lung cancer risk from radon and progeny and statement on radon. *Ann ICRP* **40**, 1-64. (doi:10.1016/j.icrp.2011.08.011).

429. Tirmarche M., Harrison J., Laurier D., Blanchardon E., Paquet F., Marsh J. 2012 Risk of lung cancer from radon exposure: contribution of recently published studies of uranium miners. *Ann ICRP* **41**, 368-377. (doi:10.1016/j.icrp.2012.06.033).

430. Tokonami S., Hosoda M., Akiba S., Sorimachi A., Kashiwakura I., Balonov M. 2012 Thyroid doses for evacuees from the Fukushima nuclear accident. *Sci Rep* **2**, 507. (doi:10.1038/srep00507).

431. Tollefsen T., Cinelli G., Bossew P., Gruber V., De Cort M. 2014 From the European indoor radon map towards an atlas of natural radiation. *Radiat Prot Dosimetry* **162**, 129-134. (doi:10.1093/rpd/ncu244).

432. Tomasek L. 2012 Lung cancer mortality among Czech uranium miners-60 years since exposure. *J Radiol Prot* **32**, 301-314. (doi:10.1088/0952-4746/32/3/301).

433. Tomasetti C., Li L., Vogelstein B. 2017 Stem cell divisions, somatic mutations, cancer etiology, and cancer prevention. *Science* **355**, 1330-1334. (doi:10.1126/science.aaf9011).

434. Tondel M., Hjalmarsson P., Hardell L., Carlsson G., Axelson O. 2004 Increase of regional total cancer incidence in north Sweden due to the Chernobyl accident? *J Epidemiol Community Health* **58**, 1011-1016. (doi:10.1136/jech.2003.017988).

435. Torres-Duran M., Ruano-Ravina A., Parente-Lamelas I., Leiro-Fernandez V., Abal-Arca J., Montero-Martinez C., Pena-Alvarez C., Gonzalez-Barcala F.J., Castro-Anon O., Golpe-Gomez A., et al. 2014 Lung cancer in never-smokers: a case-control study in a radon-prone area (Galicia, Spain). *European Respiratory Journal* **44**, 994-1001. (doi:10.1183/09031936.00017114).

436. Tran V., Zablotska L.B., Brenner A.V., Little M.P. 2017 Radiation-associated circulatory disease mortality in a pooled analysis of 77,275 patients from the Massachusetts and Canadian tuberculosis fluoroscopy cohorts. *Sci Rep* **7**, 44147. (doi:10.1038/srep44147).

437. Travis L.B., Kathren R.L., Boice J.D., Jr. 1992 Cancer risk following exposure to Thorotrast: overview in relation to a case report. *Health Phys* **63**, 89-97.

438. Travis L.B., Land C.E., Andersson M., Nyberg U., Goldman M.B., Knudson Gaul L., Berger E., Storm H.H., Hall P., Auvinen A., et al. 2001 Mortality after cerebral angiography with or without radioactive Thorotrast: an international cohort of 3,143 two-year survivors. *Radiat Res* **156**, 136-150.

439. Travis L.B., Hill D.A., Dores G.M., Gospodarowicz M., van Leeuwen F.E., Holowaty E., Glimelius B., Andersson M., Wiklund T., Lynch C.F., et al. 2003a Breast cancer following radiotherapy and chemotherapy among young women with Hodgkin disease. *JAMA* **290**, 465-475. (doi:10.1001/jama.290.4.465).

440. Travis L.B., Hauptmann M., Gaul L.K., Storm H.H., Goldman M.B., Nyberg U., Berger E., Janower M.L., Hall P., Monson R.R., et al. 2003b Site-specific cancer incidence and mortality after cerebral angiography with radioactive thorotrast. *Radiat Res* **160**, 691-706.

441. Tronko M.D., Howe G.R., Bogdanova T.I., Bouville A.C., Epstein O.V., Brill A.B., Likhtarev I.A., Fink D.J., Markov V.V., Greenebaum E., et al. 2006 A cohort study of thyroid cancer and other thyroid diseases after the Chornobyl accident: thyroid cancer in Ukraine detected during first screening. *J Natl Cancer Inst* **98**, 897-903. (doi:10.1093/jnci/djj244).

442. Tsuda T., Tokinobu A., Yamamoto E., Suzuki E. 2016a The authors respond. *Epidemiology* **27**, e21-23. (doi:10.1097/EDE.0000000000000468).

443. Tsuda T., Tokinobu A., Yamamoto E., Suzuki E. 2016b Thyroid cancer detection by ultrasound among residents ages 18 years and younger in Fukushima, Japan: 2011 to 2014. *Epidemiology* **27**, 316-322. (doi:10.1097/EDE.0000000000000385).

444. Tubiana M. 2005 Dose-effect relationship and estimation of the carcinogenic effects of low doses of ionizing radiation: The joint report of the Academie des Sciences (Paris) and of the Academie Nationale de Medecine. *International Journal of Radiation Oncology Biology Physics* **63**, 317-319. (doi:10.1016/j.ijrobp.2005.06.013).

445. Tubiana M., Feinendegen L.E., Yang C., Kaminski J.M. 2009 The linear no-threshold relationship is inconsistent with radiation biologic and experimental data. *Radiology* **251**, 13-22. (doi:10.1148/radiol.2511080671).

446. Ullrich R.L., Jernigan M.C., Satterfield L.C., Bowles N.D. 1987 Radiation carcinogenesis: time-dose relationships. *Radiat Res* **111**, 179-184.

447. UNSCEAR (United Nations Scientific Committee on the Effects of Atomic Radiation). 2000a Sources and Effects of Ionizing Radiation, Volume II, Exposures and effects of the Chernobyl accident (Annex J). Geneva.

448. UNSCEAR. 2000b Sources and Effects of Ionizing Radiation. Volume II: Effects. Geneva.

449. UNSCEAR (United Nations Scientific Committee on the Effects of Atomic Radiation). 2001 Hereditary Effects of Radiation (Annex A). Geneva.

450. UNSCEAR (United Nations Scientific Committee on the Effects of Atomic Radiation). 2006a Effects of Ionizing Radiation, Volume I (Annexes A and B). Geneva.

451. UNSCEAR (United Nations Scientific Committee on the Effects of Atomic Radiation). 2006b Effects of Ionizing Radiation, Volume II (Annexes C, D and E). Geneva.

452. UNSCEAR (United Nations Scientific Committee on the Effects of Atomic Radiation). 2008 Sources and Effects of Ionizing Radiation, Volume II (Annexes C, D and E). Geneva.

453. UNSCEAR (United Nations Scientific Committee on the Effects of Atomic Radiation). 2010 Summary of low-dose radiation effects on health. Geneva.

454. UNSCEAR (United Nations Scientific Committee on the Effects of Atomic Radiation). 2012a Biological Mechanisms of Radiation Actions at Low Doses (White Paper). Geneva.

455. UNSCEAR (United Nations Scientific Committee on the Effects of Atomic Radiation). 2012b Sources, Effects and Risks of Ionizing Radiation (Annexes A and B). Geneva.

456. UNSCEAR (United Nations Scientific Committee on the Effects of Atomic Radiation). 2013a Sources, Effects and Risks of Ionizing Radiation, Volume I, Levels and effects of radiation exposure due to the nuclear accident after the 2011 great east-Japan earthquake and tsunami (Annex A). Geneva.

457. UNSCEAR (United Nations Scientific Committee on the Effects of Atomic Radiation). 2013b Sources, Effects and Risks of Ionizing Radiation, Volume II: Effects of radiation exposure on children (Annex B). Geneva.

458. Vaiserman A.M. 2010 Radiation hormesis: historical perspective and implications for low-dose cancer risk assessment. *Dose-Response* **8**, 172-191. (doi:10.2203/dose-response.09-037.Vaiserman).

459. Valentin J. 2005 Low-dose extrapolation of radiation-related cancer risk. *Ann ICRP* **35**, 1-140. (doi:10.1016/j.icrp.2005.11.002).

460. Vasilenko E.K., Khokhryakov V.F., Miller S.C., Fix J.J., Eckerman K., Choe D.O., Gorelov M., Khokhryakov V.V., Knyasev V., Krahenbuhl M.P., et al. 2007 Mayak worker dosimetry study: an overview. *Health Phys* **93**, 190-206. (doi:10.1097/01.HP.0000266071.43137.0e).

461. Veiga L.H., Holmberg E., Anderson H., Pottern L., Sadetzki S., Adams M.J., Sakata R., Schneider A.B., Inskip P., Bhatti P., et al. 2016 Thyroid Cancer after Childhood Exposure to External Radiation: An Updated Pooled Analysis of 12 Studies. *Radiat Res* **185**, 473-484. (doi:10.1667/rr14213.1).

462. Villeneuve P.J., Morrison H.I., Lane R. 2007 Radon and lung cancer risk: An extension of the mortality follow-up of the Newfoundland fluorspar cohort. *Health Physics* **92**, 157-169. (doi:10.1097/01.hp.0000239127.43136.89).

463. Vogelstein B., Fearon E.R., Hamilton S.R., Kern S.E., Preisinger A.C., Leppert M., Nakamura Y., White R., Smits A.M., Bos J.L. 1988 Genetic alterations during colorectal-tumor development. *N Engl J Med* **319**, 525-532. (doi:10.1056/nejm198809013190901).

464. Wahba A., Lehman S.L., Tofilon P.J. 2017 Radiation-induced translational control of gene expression. *Translation* **5**, e1265703. (doi:10.1080/21690731.2016.1265703).

465. Wakeford R. 2004 The cancer epidemiology of radiation. *Oncogene* **23**, 6404-6428. (doi:10.1038/sj.onc.1207896).

466. Wakeford R. 2008 Childhood leukaemia following medical diagnostic exposure to ionizing radiation in utero or after birth. *Radiat Prot Dosimetry* **132**, 166-174. (doi:10.1093/rpd/ncn272).

467. Wakeford R. 2009 Radiation in the workplace-a review of studies of the risks of occupational exposure to ionising radiation. *J Radiol Prot* **29**, A61-79. (doi:10.1088/0952-4746/29/2a/s05).

468. Wakeford R., Darby S.C., Murphy M.F. 2010 Temporal trends in childhood leukaemia incidence following exposure to radioactive fallout from atmospheric nuclear weapons testing. *Radiat Environ Biophys* **49**, 213-227. (doi:10.1007/s00411-010-0266-4).

469. Wakeford R. 2013 The risk of childhood leukaemia following exposure to ionising radiation--a review. *J Radiol Prot* **33**, 1-25. (doi:10.1088/0952-4746/33/1/1).

470. Wakeford R. 2014a Childhood leukaemia and nuclear installations: the long and winding road. *British Journal of Cancer* **111**, 1681-1683. (doi:10.1038/bjc.2014.517).

471. Wakeford R. 2014b Nuclear worker studies: promise and pitfalls. *Br J Cancer* **110**, 1-3. (doi:10.1038/bjc.2013.713).

472. Wakeford R. 2015 Association and causation in epidemiology - half a century since the publication of Bradford Hill's interpretational guidance. *J R Soc Med* **108**, 4-6. (doi:10.1177/0141076814562713).

473. Wakeford R. 2016 Chernobyl and Fukushima-where are we now? *J Radiol Prot* **36**, E1-E5. (doi:10.1088/0952-4746/36/2/E1).

474. Wakeford R., Auvinen A., Gent R.N., Jacob P., Kesminiene A., Laurier D., Schuz J., Shore R., Walsh L., Zhang W. 2016 Re: thyroid cancer among young people in Fukushima. *Epidemiology* **27**, e20-21. (doi:10.1097/EDE.0000000000000466).

475. Walsh L., Shore R., Auvinen A., Jung T., Wakeford R. 2014 Risks from CT scans—what do recent studies tell us? *J Radiol Prot* **34**, E1.

476. Walsh L., Grosche B., Schnelzer M., Tschense A., Sogl M., Kreuzer M. 2015 A review of the results from the German Wismut uranium miners cohort. *Radiat Prot Dosimetry* **164**, 147-153. (doi:10.1093/rpd/ncu281).

477. Wang J.X., Zhang L.A., Li B.X., Zhao Y.C., Wang Z.Q., Zhang J.Y., Aoyama T. 2002 Cancer incidence and risk estimation among medical x-ray workers in China, 1950-1995. *Health Phys* **82**, 455-466.

478. Weinberg H.S., Korol A.B., Kirzhner V.M., Avivi A., Fahima T., Nevo E., Shapiro S., Rennert G., Piatak O., Stepanova E.I., et al. 2001 Very high mutation rate in offspring of Chernobyl accident liquidators. *Proc Biol Sci* **268**, 1001-1005. (doi:10.1098/rspb.2001.1650).

479. Weiss H.A., Darby S.C., Doll R. 1994 Cancer mortality following X-ray treatment for ankylosing spondylitis. *Int J Cancer* **59**, 327-338.

480. Weiss H.A., Darby S.C., Fearn T., Doll R. 1995 Leukemia mortality after X-ray treatment for ankylosing spondylitis. *Radiat Res* **142**, 1-11.

481. Wernli C. 2016 A short history and critical review of individual monitoring. *Radiat Prot Dosimetry*. (doi:10.1093/rpd/ncw025).

482. Wertelecki W. 2010 Malformations in a Chornobyl-impacted region. *Pediatrics* **125**, e836-843. (doi:10.1542/peds.2009-2219).

483. WHO. 2006 Health Effects of the Chernobyl Accident and Special Health Care Programmes. In *Report of the UN Chernobyl Forum Expert Group "Health"* (ed. Bennett B., Repacholi, M., Carr, Z.). Geneva.

484. Wick R.R., Nekolla E.A., Gossner W., Kellerer A.M. 1999 Late effects in ankylosing spondylitis patients treated with 224Ra. *Radiat Res* **152**, S8-s11.

485. Williams D. 2015 Thyroid Growth and Cancer. *Eur Thyroid J* **4**, 164-173. (doi:10.1159/000437263).

486. Wolff S. 1996 Aspects of the adaptive response to very low doses of radiation and other agents. *Mutation Research/Fundamental and Molecular Mechanisms of Mutagenesis* **358**, 135-142. (doi:https://doi.org/10.1016/S0027-5107(96)00114-5).

487. Worgul B.V., Merriam G.R., Jr., Medvedovsky C. 1989 Cortical cataract development--an expression of primary damage to the lens epithelium. *Lens Eye Toxic Res* **6**, 559-571.

488. Worgul B.V., Kundiyev Y.I., Sergiyenko N.M., Chumak V.V., Vitte P.M., Medvedovsky C., Bakhanova E.V., Junk A.K., Kyrychenko O.Y., Musijachenko N.V., et al. 2007 Cataracts among Chernobyl clean-up workers: implications regarding permissible eye exposures. *Radiat Res* **167**, 233-243.

489. Wrixon A.D. 2008 New ICRP recommendations. *J Radiol Prot* **28**, 161-168. (doi:10.1088/0952-4746/28/2/r02).

490. Yablokov A.V., Nesterenko V.B. 2009 Consequences of the Chernobyl catastrophe. Preface. *Ann N Y Acad Sci* **1181**, x-xiii. (doi:10.1111/j.1749-6632.2009.04817.x).

491. Yamada M., Wong F.L., Fujiwara S., Akahoshi M., Suzuki G. 2004 Noncancer disease incidence in atomic bomb survivors, 1958-1998. *Radiat Res* **161**, 622-632.

492. Yentrapalli R., Azimzadeh O., Sriharshan A., Malinowsky K., Merl J., Wojcik A., Harms-Ringdahl M., Atkinson M.J., Becker K.F., Haghdoost S., et al. 2013a The PI3K/Akt/mTOR pathway is implicated in the premature senescence of primary human endothelial cells exposed to chronic radiation. *PLoS One* **8**, e70024. (doi:10.1371/journal.pone.0070024).

493. Yentrapalli R., Azimzadeh O., Barjaktarovic Z., Sarioglu H., Wojcik A., Harms-Ringdahl M., Atkinson M.J., Haghdoost S., Tapio S. 2013b Quantitative proteomic analysis reveals induction of

premature senescence in human umbilical vein endothelial cells exposed to chronic low-dose rate gamma radiation. *Proteomics* **13**, 1096-1107. (doi:10.1002/pmic.201200463).

494. Yong L.C., Pinkerton L.E., Yiin J.H., Anderson J.L., Deddens J.A. 2014 Mortality among a cohort of US commercial airline cockpit crew. *Am J Ind Med* **57**, 906-914. (doi:10.1002/ajim.22318).

495. Yoshinaga S., Aoyama T., Yoshimoto Y., Sugahara T. 1999 Cancer mortality among radiological technologists in Japan: updated analysis of follow-up data from 1969 to 1993. *J Epidemiol* **9**, 61-72.

496. Yoshinaga S., Mabuchi K., Sigurdson A.J., Doody M.M., Ron E. 2004 Cancer risks among radiologists and radiologic technologists: review of epidemiologic studies. *Radiology* **233**, 313-321. (doi:10.1148/radiol.2332031119).

497. Zablotska L.B., Bogdanova T.I., Ron E., Epstein O.V., Robbins J., Likhtarev I.A., Hatch M., Markov V.V., Bouville A.C., Olijnyk V.A., et al. 2008 A cohort study of thyroid cancer and other thyroid diseases after the Chornobyl accident: dose-response analysis of thyroid follicular adenomas detected during first screening in Ukraine (1998-2000). *Am J Epidemiol* **167**, 305-312. (doi:10.1093/aje/kwm301).

498. Zablotska L.B., Ron E., Rozhko A.V., Hatch M., Polyanskaya O.N., Brenner A.V., Lubin J., Romanov G.N., McConnell R.J., O'Kane P., et al. 2011 Thyroid cancer risk in Belarus among children and adolescents exposed to radioiodine after the Chornobyl accident. *Br J Cancer* **104**, 181-187. (doi:10.1038/sj.bjc.6605967).

499. Zablotska L.B., Bazyka D., Lubin J.H., Gudzenko N., Little M.P., Hatch M., Finch S., Dyagil I., Reiss R.F., Chumak V.V., et al. 2013 Radiation and the risk of chronic lymphocytic and other leukemias among chornobyl cleanup workers. *Environ Health Perspect* **121**, 59-65. (doi:10.1289/ehp.1204996).

500. Zablotska L.B., Lane R.S., Thompson P.A. 2014 A reanalysis of cancer mortality in Canadian nuclear workers (1956-1994) based on revised exposure and cohort data. *Br J Cancer* **110**, 214-223. (doi:10.1038/bjc.2013.592).

501. Zablotska L.B., Nadyrov E.A., Polyanskaya O.N., McConnell R.J., O'Kane P., Lubin J., Hatch M., Little M.P., Brenner A.V., Veyalkin I.V., et al. 2015 Risk of thyroid follicular adenoma among children and adolescents in Belarus exposed to iodine-131 after the Chornobyl accident. *Am J Epidemiol* **182**, 781-790. (doi:10.1093/aje/kwv127).

502. Zhao S., Weng Y.C., Yuan S.S., Lin Y.T., Hsu H.C., Lin S.C., Gerbino E., Song M.H., Zdzienicka M.Z., Gatti R.A., et al. 2000 Functional link between ataxia-telangiectasia and Nijmegen breakage syndrome gene products. *Nature* **405**, 473-477. (doi:10.1038/35013083).
